# Plasma proteomic signatures of subclinical atherosclerosis progression for cardiovascular risk stratification: A cohort study

**DOI:** 10.1371/journal.pmed.1005265

**Published:** 2026-10-20

**Authors:** Yani Huang, Ruixin Gao, Jianxin Li, Bin Yang, Keyong Huang, Shufeng Chen, Jie Cao, Xiaoqing Liu, Chong Shen, Ling Yu, Liancheng Zhao, Ying Li, Dongsheng Hu, Jianfeng Huang, Bin Lu, Dongfeng Gu, Fangchao Liu, Xiangfeng Lu

**Affiliations:** 1 Department of Epidemiology, State Key Laboratory of Cardiovascular Disease, Fuwai Hospital, National Center for Cardiovascular Diseases, Chinese Academy of Medical Sciences and Peking Union Medical College, Beijing, China; 2 Division of Epidemiology, Guangdong Provincial People’s Hospital and Cardiovascular Institute, Guangzhou, China; 3 Department of Epidemiology and Biostatistics, Center for Global Health, School of Public Health, Nanjing Medical University, Nanjing, China; 4 Department of Cardiology, Fujian Provincial Hospital, Fuzhou, China; 5 Department of Epidemiology and Health Statistics, College of Public Health, Zhengzhou University, Zhengzhou, China; 6 Department of Prevention Medicine, Shenzhen University School of Medicine, Shenzhen, China; 7 Department of Radiology, Fuwai Hospital, National Center for Cardiovascular Diseases, Chinese Academy of Medical Sciences and Peking Union Medical College, Beijing, China; 8 School of Medicine, Southern University of Science and Technology, Shenzhen, China; Peking University, CHINA

## Abstract

**Background:**

Existing risk stratification tools inadequately identify individuals with subclinical atherosclerosis (SA) who could benefit from early intervention. This study aimed to develop protein risk scores (ProtRSs) for SA progression and assess their incremental value for atherosclerotic cardiovascular disease (ASCVD) risk prediction.

**Methods and findings:**

In a community cohort consisting of 903 individuals undergoing repeated SA assessments (coronary artery calcium [CAC] or carotid plaque) over 6 years (2008–2009 to 2013–2014), protein biomarkers were selected from a panel of 117 proteins using least absolute shrinkage and selection operator (LASSO) regression to construct SA ProtRSs for CAC and plaque progression, respectively, and their predictive abilities for incident ASCVD risk were evaluated based on a guideline-recommended traditional risk score in a case-cohort of 1,835 individuals (2007–2008 to 2018–2021, median follow-up of 10.4 years). The ProtRSs each included 14 proteins for CAC and plaque progression, explaining 17.1% and 18.4% of respective variances. Effect sizes of these proteins on SA progression showed strong concordance with those on incident ASCVD (*r*  > 0.75). Each standard deviation increase in CAC and plaque ProtRSs was associated with hazard ratios of 1.36 (95% confidence interval [CI] [1.20,1.54]) and 1.38 (95% CI [1.23,1.54]) for ASCVD risk, respectively. Addition of the SA ProtRSs to the traditional risk score yielded a net reclassification improvement of 0.127 (*p* < 0.001). Importantly, 18.6% of traditional intermediate-risk individuals were reclassified into the high-risk category (10-year ASCVD event rate: 10.9%), indicating the need for preventive interventions, whereas 32.8% of traditional high-risk individuals were identified as having an even higher rate (13.7%), suggesting the requirement for intensified management. The main limitation is that the predictive value of ProtRSs warrants further large-scale external validation.

**Conclusions:**

SA ProtRSs of coronary calcification and carotid plaque progression may provide additional value over traditional risk assessment, highlighting their potential in refining risk stratification and guiding targeted intervention strategies.

## Author summary

**Why was this study done?:**

- Current risk scores are used to estimate the risk of developing cardiovascular disease, but their predictive performance could be improved, particularly among individuals classified as being at intermediate-risk.
- Plasma proteins may capture early vascular changes involved in cardiovascular disease progression. However, evidence remains limited on whether these protein biomarkers could improve risk prediction or help identify individuals whose future risk may be underestimated by current risk scores.

**What did the researchers do and find?:**

- We conducted a prospective cohort study of 903 participants with 117 blood proteins measured to identify biomarkers of atherosclerosis progression, and an ASCVD case-cohort study of 1,835 participants to evaluate their performance in predicting incident ASCVD.
- We identified two sets of proteins that separately predicted coronary calcium and carotid plaque progression, and developed the corresponding protein risk scores that improved prediction beyond current risk scores.
- To support clinical translation, we showed that combining these protein risk scores with current risk scores could help identify individuals at high-risk of cardiovascular disease, particularly among those with traditional intermediate-risk.

**What do these findings mean?:**

- This study highlights the potential of plasma protein biomarkers to improve prediction of early progression of cardiovascular disease.
- The protein risk scores could improve the classification of future cardiovascular disease risk beyond current risk assessment methods, particularly among people whose risk may be underestimated.
- The key limitation is that the predictive value of ProtRSs for cardiovascular risk warrants further large-scale external validation.

## Abstract 3

Yani Juang, Ruixin Gao and colleagues developed plasma protein-based risk scores linked to the progression of subclinical atherosclerosis. They found that combining these protein-based risk scores with traditional risk factors could improve prediction of cardiovascular disease risk, but further validation is needed.

## Introduction

Atherosclerotic cardiovascular disease (ASCVD) remains a major contributor to global disease burden and mortality [1,2]. While current clinical guidelines advocate for risk assessment tools based on established cardiovascular risk factors in primary prevention, emerging evidence reveals significant limitations in traditional risk stratification [3,4]. Specifically, some individuals deemed at intermediate- or even low-risk by traditional risk scores may still harbor a substantial atherosclerotic burden and be susceptible to disease progression [5]. This gap underscores the importance of detecting subclinical atherosclerosis (SA), an early and pivotal stage of ASCVD in which preventive measures could have a stronger impact [6]. Currently, guidelines recommend imaging-based coronary artery calcium (CAC) scoring as an enhancing factor of ASCVD risk assessment [7,8]. However, the feasibility of widespread CAC scanning is limited by restricted access to computed tomography (CT) equipment, radiation exposure, and cost-effectiveness concerns [7]. Thus, the development of sensitive and widely accessible approaches to assess atherosclerotic burden is urgently needed.

Recent advances in proteomics have enabled large-scale population studies to quantify plasma protein levels [9]. Several European cohort studies have discovered protein biomarkers associated with atherosclerotic lesions, such as growth/differentiation factor 15 (GDF-15) and T-cell immunoglobulin and mucin domain (TIM)-1 [10,11]. However, most of these studies were based on a cross-sectional design, making it difficult to determine whether protein alterations precede the development of atherosclerosis or merely reflect its concurrent pathology. This limitation underscores the need to identify protein biomarkers driving SA progression in prospective cohorts, which would offer predictive value for future ASCVD events [12]. Moreover, the extent to which these protein biomarkers improve risk stratification beyond traditional scoring remains understudied, particularly in non-European populations.

To address these gaps, this study aimed to (1) identify candidate plasma proteins driving the development of SA among 903 Chinese adults who underwent repeated CAC or carotid ultrasound measurements over a 6-year period, (2) incorporate these proteins into protein risk scores (ProtRSs) for SA progression, and (3) evaluate whether these ProtRSs can enhance ASCVD prediction beyond traditional risk scores in a prospective case-cohort of 1,835 participants.

## Methods

### Ethics statement

All studies were approved by the Institutional Review Board at Fuwai Hospital (Approval number 2022-1665) and written informed consent was obtained from each participant prior to data collection.

### Study design and population

The study design is illustrated in Fig 1, and a prospective protocol, including changes made in response to the reviewers’ comments, is presented in S1 Text. Based on a discovery population with repeated SA examinations, protein biomarkers were selected by least absolute shrinkage and selection operator (LASSO) regression and used to construct the ProtRSs for SA progression. The predictive performance of the ProtRSs for incident ASCVD risk was assessed in a case-cohort.

**Fig 1:**
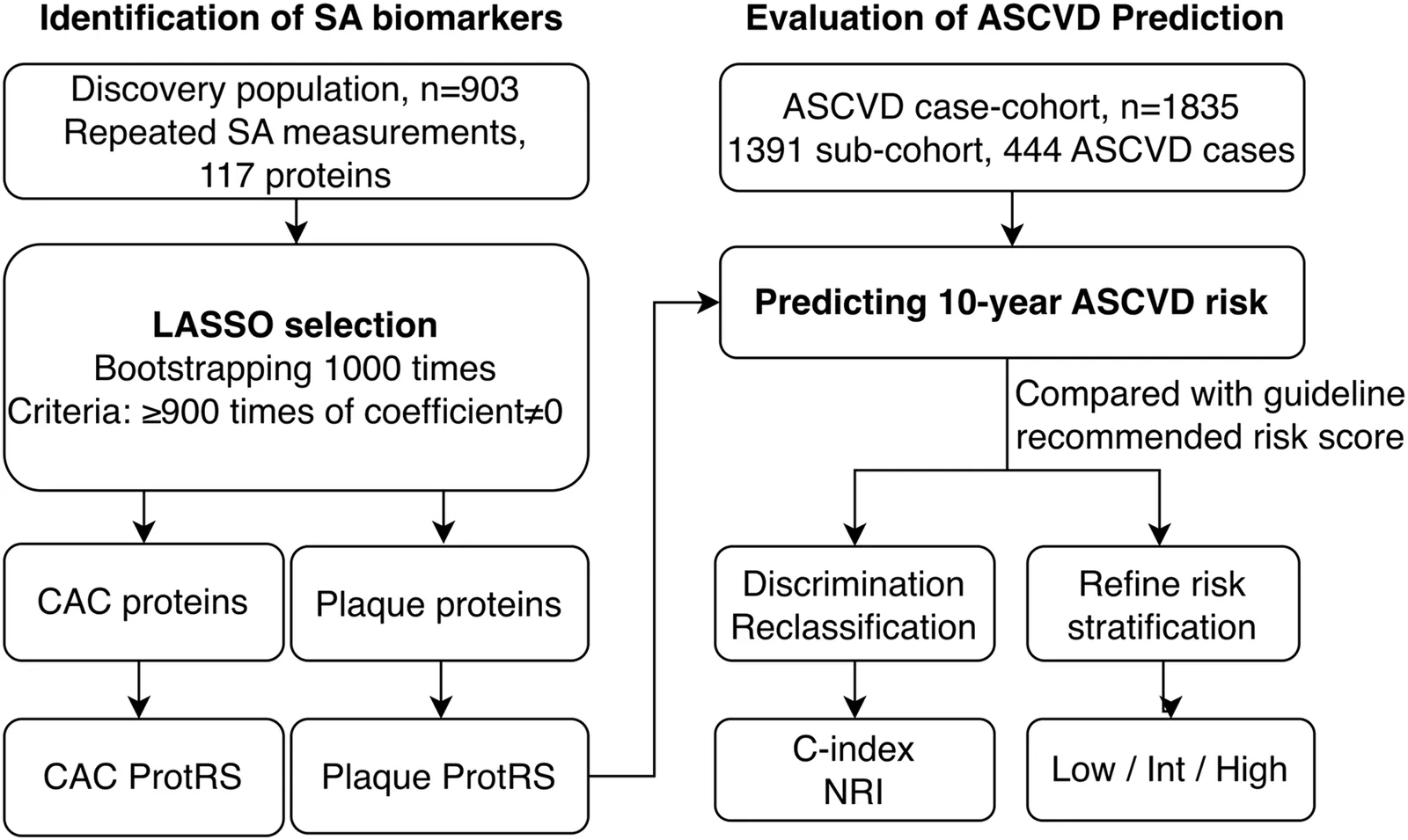
Flowchart of the study. Abbreviations: SA, subclinical atherosclerosis; LASSO, least absolute shrinkage and selection operator; CAC, coronary artery calcium; ASCVD, atherosclerotic cardiovascular disease; ProtRS, protein risk score; NRI, net reclassification improvement; Int, intermediate.

For the discovery cohort, 1,087 participants from the Beijing Community-Based Cohort of Atherosclerosis Study [13] were initially enrolled in 2008–2009 and were followed up at 2013–2014. After excluding 184 participants without protein data, 903 participants were retained for subsequent analysis. Among them, 815 and 781 were available for CAC and plaque measurements, respectively (Fig AA in S1 Appendix).

For the validation cohort, participants were included based on a case-cohort design nested within the International Collaborative Study of Cardiovascular Disease in Asia (InterASIA) [14] and the China Multi-Center Collaborative Study of Cardiovascular Epidemiology (ChinaMUCA) cohorts [15]. At baseline (2007–2008), participants free of ASCVD were enrolled. The case-cohort was constructed by including all incident ASCVD cases that developed during follow-up (up to 2018–2021) and a random 10% sub-cohort of the overall participants. This initial case-cohort comprised 1,449 sub-cohort individuals and 501 additional incident cases after a median follow-up period of 10.4 years. After excluding 115 participants without protein data, 1,835 individuals were retained for the analysis (Fig AB in S1 Appendix). Specifically, the InterASIA cohort included 758 sub-cohort individuals (44 ASCVD cases) and 272 additional cases while the ChinaMUCA cohort included 633 sub-cohort individuals (21 ASCVD cases) and 172 additional cases outside the sub-cohort.

This study is reported as per the Strengthening the Reporting of Observational Studies in Epidemiology (STROBE) guideline (S1 Checklist) and the Transparent Reporting Of a Multivariate Prediction Model (TRIPOD) guideline (S2 Checklist).

### Data collection and outcome definition

Baseline information, including demographics, lifestyle risk factors, medical history, and laboratory variables, was collected by trained staff through physical examinations and questionnaire interviews as previously published [15]. Blood samples were collected after an overnight fast of at least 10 hours, processed for plasma separation, and stored at −80 °C (S1 Text).

CAC scanning was performed at Fuwai Hospital using a LightSpeed VCT 64-slice CT scanner (General Electric Healthcare, U.S.) and CAC was scored using the Agatston approach [16]. Baseline CAC was classified into three categories: CAC = 0 (not detectable), 0 < CAC < 100 (mild), and CAC ≥ 100 (moderate to severe). Based on previously widely used criteria, CAC progression was defined as one of the following conditions: (1) CAC > 0 at follow-up for participants with baseline CAC = 0; (2) an annualized CAC change ≥10 at follow-up for those with baseline 0 < CAC < 100; (3) an annualized percent change in CAC ≥ 10% for those with baseline CAC ≥ 100 [17–19].

Carotid ultrasound was conducted using an iE33 ultrasound station (Philips Healthcare, Netherland) equipped with a 7.5-MHz linear array probe. Carotid plaque was defined as a focal intima-media thickness exceeding 1.5 mm or focal thickening at least 50% greater than the surrounding wall in the common carotid artery, carotid bifurcation, and internal carotid artery. The total number of plaques in bilateral carotid arteries was counted and plaque progression was defined as an increase in the number of plaques at follow-up compared to baseline according to the Multi-Ethnic Study of Atherosclerosis (MESA) study [20].

Incident ASCVD was defined as the first occurrence of nonfatal acute myocardial infarction, coronary heart disease (CHD) death, or fatal or nonfatal ischemic stroke (IS). For both baseline and follow-up, the outcomes were collected via interviews with participants or their proxies, and validated using hospital records or death certificates, as described in the S1 Text. The absence of ASCVD at baseline was defined as no clinical diagnosis of incident ASCVD by a physician, nor self-reported incident ASCVD during interviews with participants or their proxies before 2007–2008. Person-years of follow-up for each participant were calculated as the time interval between the baseline and the earliest occurrence of ASCVD, death, or last follow-up, whichever came first.

### Proteomics measurements

Plasma levels of 117 proteins were quantified in this study, which are involved in ASCVD-related pathways such as inflammation, immune response, and lipid metabolism (Table A in S2 Appendix). Protein measurement was performed using three platforms: the Olink Cardiovascular-III panel using proximity extension assay technology for 92 proteins [21], an automated ELISA-based microfluidic system (ELLA, ProteinSimple) for 14 inflammatory proteins, and clinical biochemistry assays on Beckman Coulter AU680 for 11 metabolic proteins. All measured concentrations underwent Z-score standardization (mean = 0, standard deviation [SD] = 1). Principal component analysis revealed no platform-specific clustering patterns (Fig B in S1 Appendix).

### Protein risk score

LASSO regression with 10-fold cross-validation was performed in 1,000 iterations (randomly selected 90% sample size) to select proteins associated with CAC or plaque progression, adjusting for age and sex. Proteins retaining nonzero coefficients in ≥ 900 iterations for each outcome were subsequently incorporated into two distinct ProtRSs through logistic regression modeling. Final ProtRS values were calculated on the logit scale as the sum of each protein’s regression coefficient multiplied by its standardized plasma level.

### Statistical analysis

Data are presented as mean (SD) or median (quartile 1 [Q1], quartile 3 [Q3]) for continuous variables and as frequency (%) for categorical variables.

Based on percentiles of ProtRSs in the population [22], participants were categorized into: low (bottom 20%), intermediate (middle 60%), and high (top 20%) groups. Log-binomial regressions were used to estimate relative risks (RRs) and 95% confidence intervals (CIs) of ProtRSs for CAC and plaque progression, respectively, adjusting for traditional cardiovascular disease (CVD) risk factors including baseline age, sex, urbanization, smoking status, waist circumference, systolic blood pressure, antihypertensive therapy, total cholesterol, high-density lipoprotein cholesterol, and diabetes. The *p*_trend_ was calculated by taking the medians of each protein risk category (low, intermediate, and high) as continuous numeric variables with the hypothesis that the risk of CAC and plaque progression increases with the higher risk categories in protein risk categories.

To evaluate the predictive accuracy of SA ProtRSs for the risk of SA progression, area under the receiver operating characteristic (ROC) curve (AUC) values were estimated for each ProtRS, and their comparisons with a reference model including the above-mentioned traditional CVD risk factors were assessed by DeLong’s test [23]. Calibration plots were used to assess the agreement between the observed risk of SA progression and the predicted risk by traditional risk factors with the addition of ProtRSs. To further identify proteins with biological relevance, logistic regression models with forward method were used, and Over-Representation Analysis (ORA) was performed to investigate which particular pathways from the Kyoto Encyclopedia of Genes and Genomes (KEGG) were enriched among the biologically related-proteins.

For the risk of incident ASCVD, Prentice Cox regressions were used to estimate hazard ratios (HRs) and 95% CIs of ProtRSs in the case-cohort [24]. We hypothesized that proteins exerting stronger effects on SA progression would confer a higher risk of incident ASCVD, and Pearson correlation analysis was performed to compare the effect sizes of these proteins between SA progression and ASCVD incidence. The incremental value of ProtRSs was assessed over the guideline-recommended Prediction for Atherosclerotic cardiovascular disease Risk in China (China-PAR) risk score, which is a sex-specific risk equation for the 10-year risk of ASCVD incorporating age, geographic region, urbanization, systolic blood pressure, antihypertensive treatment, total cholesterol, high-density lipoprotein cholesterol, waist circumference, current smoking status, diabetes mellitus, family history of ASCVD and available interaction terms for age with risk factors that met predefined statistical criteria [15]. Due to the absence of hemorrhagic stroke in the current study outcome compared with the original China-PAR risk score, when calculating the absolute observed ASCVD event rate, the baseline survival function (S_0_) was re-estimated among the entire cohort (*n* = 14,496) and thresholds of < 5%, 5%–10%, and ≥ 10% were set to define China-PAR low-, intermediate-, and high-risk groups.

The prediction performance of ProtRSs and their added value to the reference risk score were evaluated by Harrell’s C-index through 1,000 bootstrapping iterations [25,26]. In addition, categorical net reclassification improvement (NRI) was estimated based on cut-off values of 5% and 10%, and the *p*-value of categorical NRI was calculated through 1,000 bootstrapping iterations. Cases that developed outside the sub-cohort after 10 years were excluded according to methods applied to case-cohort design [25]. To explore the clinical implications for intermediate-risk individuals, the NRI was further calculated among participants with a China-PAR risk of 5–10% [27].

Furthermore, risk stratification achieved by SA ProtRSs beyond the China-PAR risk score was evaluated within a 10-year timeframe by estimating the group-specific observed risks. The SA protein risk category was combined as high (high CAC and high plaque ProtRS), intermediate (only high CAC or only high plaque ProtRS), and low (low or intermediate CAC and low or intermediate plaque ProtRS). The observed risk of incident ASCVD for each category was estimated using the Kaplan-Meier method and a weighting of 1/(sampling proportion) was applied to the sub-cohort individuals to compensate for the over-representation of cases.

All statistical tests with *p* < 0.05 were considered statistically significant. Multiple testing was adjusted using the false discovery rate (FDR) method. Statistical analyses were performed in R version 4.4.3.

### Artificial intelligence statement

No artificial intelligence tools or technologies were used to generate, edit, or analyze the study, data, or article content. The authors take full responsibility for the final manuscript.

## Results

### Baseline characteristics

Baseline characteristics of the study populations are summarized in Table 1. In the Beijing Community-Based Cohort (*n* = 903), the mean (SD) age was 54.3 (6.4) years, and 54.9% were female. At baseline, 246 (30.2%) participants had detectable CAC (CAC > 0), and 305 (39.1%) participants had at least one carotid plaque. Over a median follow-up of 6.1 years, 272 (33.4%) participants experienced CAC progression, and 172 (22.0%) exhibited plaque progression. In progressors, the baseline age, proportion of smokers, blood pressure, triglycerides, total cholesterol, prevalence of diabetes, and the use of medications were higher than nonprogressors (Table B in S2 Appendix). In the ASCVD case-cohort, the mean (SD) age of the sub-cohort was 55.9 (8.7) years, with 55.0% being female. The baseline characteristics of the sub-cohort were comparable to those of the overall cohort (Table C in S2 Appendix). After a median follow-up period of 10.4 years, an additional 444 individuals developed ASCVD outside the sub-cohort.

**Table 1:** Baseline characteristics of study populations.

Baseline characteristics	Beijing community-Based cohort of atherosclerosis study (*n* = 903)	ASCVD case-cohort (*n* = 1,835)	
		Sub-cohort (*n* = 1,391)	Cases (*n* = 444)
Age, mean (SD), years	54.3 (6.4)	55.9 (8.7)	61.4 (8.8)
Female, *n* (%)	496 (54.9)	765 (55.0)	189 (42.6)
Urban region, *n* (%)	328 (36.3)	470 (33.8)	143 (32.2)
Northern China, *n* (%)	903 (100.0)	644 (46.3)	237 (53.4)
High school education or above, *n* (%)	309 (34.2)	367 (26.4)	82 (18.5)
Smoking status, *n* (%)			
Never	544 (60.2)	933 (67.1)	262 (59.0)
Former	72 (8.0)	72 (5.2)	38 (8.6)
Current	287 (31.8)	386 (27.7)	144 (32.4)
Alcohol drinking, *n* (%)	318 (35.2)	360 (25.9)	125 (28.2)
Systolic blood pressure, mean (SD), mm Hg	134.3 (18.1)	133.9 (20.6)	145.4 (23.9)
Diastolic blood pressure, mean (SD), mm Hg	83.4 (10.5)	81.5 (11.7)	85.6 (13.7)
Treated hypertension, *n* (%)	223 (24.7)	225 (16.2)	139 (31.3)
Waist circumference, mean (SD), cm	89.0 (10.1)	81.5 (10.4)	83.6 (10.5)
LDL-C, mean (SD), mmol/L	3.0 (0.8)	2.7 (0.8)	2.8 (0.9)
HDL-C, mean (SD), mmol/L	1.3 (0.3)	1.4 (0.4)	1.4 (0.3)
Total cholesterol, mean (SD), mmol/L	5.2 (0.9)	4.9 (0.9)	5.0 (1.0)
Triglycerides, median [Q1, Q3], mmol/L	1.4 [1.0, 2.1]	1.3 [0.9,1.8]	1.5 [1.0,2.2]
Treated hyperlipidemia, *n* (%)	32 (3.5)	31 (2.2)	17 (3.8)
Diabetes mellitus, *n* (%)	141 (15.6)	112 (8.1)	92 (20.7)
Treated diabetes, *n* (%)	79 (8.7)	48 (3.5)	40 (9.0)
Family history of ASCVD, *n* (%)	482 (53.4)	391 (28.1)	141 (31.8)
CAC severity, *n* (%)			
CAC = 0	569 (69.8)	–	–
CAC 1–100	168 (20.6)	–	–
CAC > 100	78 (9.6)	–	–
Number of carotid plaques, *n* (%)			
No plaque	476 (61.0)	–	–
1-3	294 (37.6)	–	–
> 3	11 (1.4)	–	–

Values are presented as mean (SD), median [Q1, Q3], or *n* (%). Abbreviations: Q1, quartile 1; Q3, quartile 3; ASCVD, atherosclerotic cardiovascular diseases; SD, standard deviation; HDL-C, high-density lipoprotein cholesterol; LDL-C, low-density lipoprotein cholesterol; CAC, coronary artery calcium; SI conversion factor: to convert total cholesterol, LDL-C, and HDL-C to mg/dL, multiply by 38.6.

### Constructing protein risk scores driving SA progression

Using LASSO regression, 14 proteins were selected to reflect the risk of CAC progression (10 positively and 4 negatively) and 14 proteins for plaque progression (9 positively and 5 negatively). Four proteins, cathepsin D (CTSD), small dense low-density lipoprotein (sdLDL), interleukin-2 receptor subunit alpha (IL-2RA), and paraoxonase 3 (PON3) (Fig 2) were selected for both CAC and plaque progression. CTSD showed the largest positive effect size for CAC progression (median effect size: 1.16; Q1-Q3: 1.13–1.18), while kallikrein-6 (KLK6) showed the largest negative effect (median effect size: 0.89; Q1-Q3: 0.87–0.91). For plaque progression, insulin (median effect size: 1.15; Q1-Q3: 1.13–1.17) and carboxypeptidase B (CPB1) (median effect size: 0.88; Q1-Q3: 0.86–0.90) had the largest positive and negative effect sizes, respectively (Table D in S2 Appendix).

**Fig 2:**
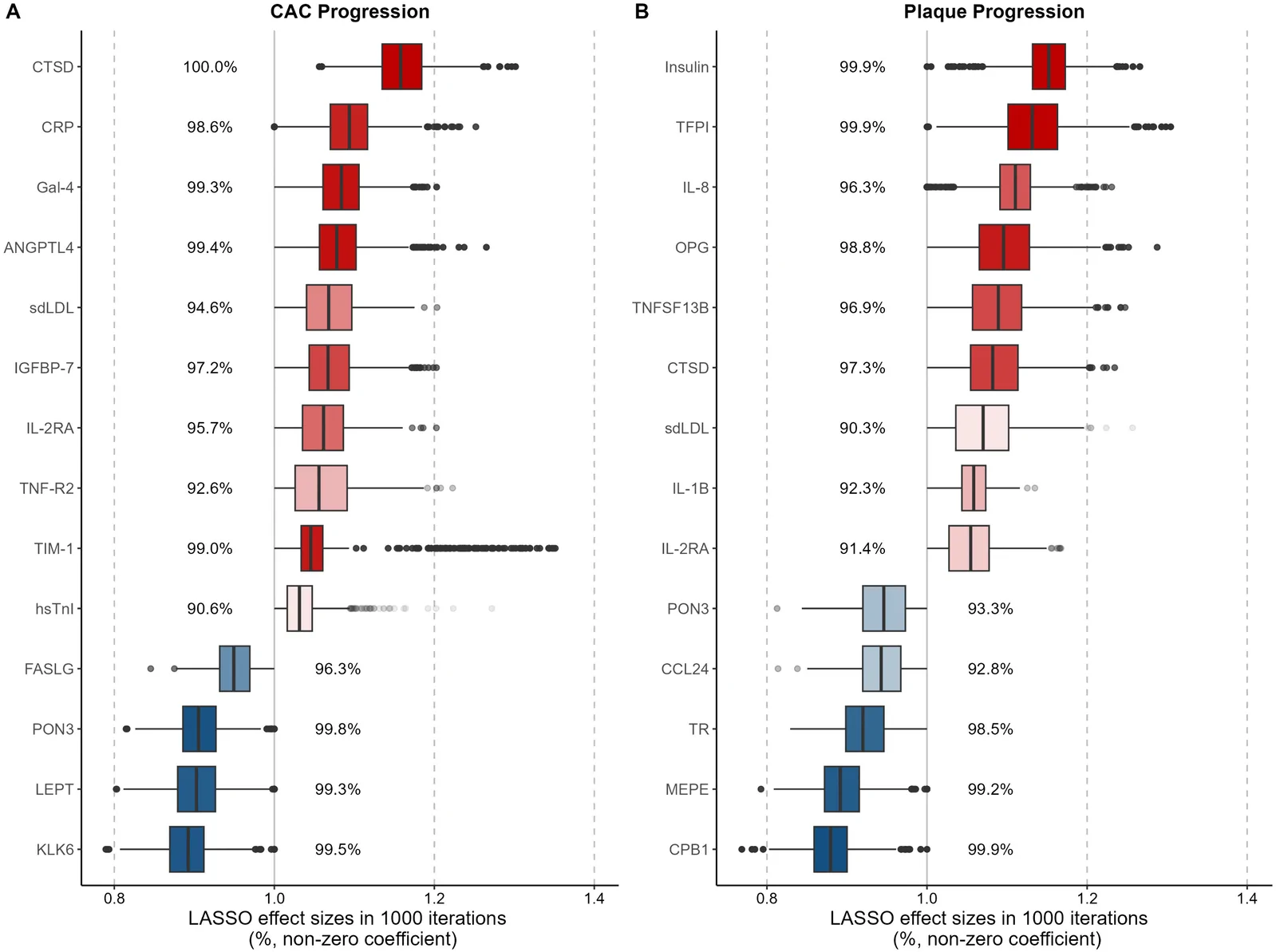
Protein biomarkers of CAC and plaque progression. Boxplots depict the distributions of coefficients from 1,000 times of LASSO regression. The box bounds the IQR divided by the median, and Tukey-style whiskers extend to a maximum of 1.5 × IQR beyond the box. Percents are proportions of nonzero coefficients. **(a)** 14 proteins selected for CAC progression. **(b)** 14 proteins selected for plaque progression. Abbreviations: CAC, coronary artery calcium; IQR, interquartile range; LASSO, least absolute shrinkage and selection operator.

We further conducted forward stepwise logistic regression to identify the proteins with potential biological relevance to atherosclerosis progression for establishing an optimal prediction panel. In the final models, 23 and 19 proteins were retained for CAC and plaque progression, respectively. Notably, most LASSO-derived proteins were consistently included in these forward regression models: 11 out of 14 CAC-related proteins (excluding TIM-1, tumor necrosis factor ligand superfamily member 6 [FASLG], and troponin) and 13 out of 14 plaque-related proteins (excluding IL-2RA) (Table E in S2 Appendix). Furthermore, protein effect sizes estimated from LASSO and forward stepwise logistic regression exhibited extremely strong correlations, with Pearson correlation coefficients of 0.976 (95% CI [0.918,0.993]; *p* < 0.001) for plaque progression and 0.899 (95% CI [0.650,0.974]; *p* < 0.001) for CAC progression. Subsequent KEGG pathway analysis revealed that a set of proteins within the optimal panel for plaque progression (e.g., C-C motif chemokine 24 [CCL-24] and interleukin-8 [IL-8]) and those for CAC progression (e.g., tumor necrosis factor receptor 2 [TNF-R2] and urokinase-type plasminogen activator [uPA]) were linked to inflammatory processes, including cytokine signaling and NF-κB activation. Other proteins, such as low-density lipoprotein receptor within the CAC progression panel and monocyte chemotactic protein 1 (MCP-1) within the plaque progression panel, were involved in lipid metabolism and atherosclerosis (Table F in S2 Appendix).

Two SA ProtRSs for CAC progression and plaque progression were constructed based on LASSO-selected biomarkers (formulas and cut-off values were shown in Table G in S2 Appendix). Collectively, these scores explained 17.1% and 18.4% of the variance in CAC and plaque progression, respectively. As expected, SA progression rates increased across ProtRSs groups, with CAC progression occurring in 14.6%, 30.8%, and 60.6% of individuals in the low, intermediate, and high CAC ProtRS groups, respectively. Notably, even among individuals categorized as having low- and intermediate-traditional risk, those in the high CAC ProtRS group still exhibited substantial CAC progression rates of 22.7% and 58.3%, respectively. A similar dose-response relationship was observed for plaque progression across plaque ProtRS strata, and individuals in the high plaque ProtRS group within the traditional low- and intermediate-risk categories also demonstrated marked plaque progression (Fig 3).

**Fig 3:**
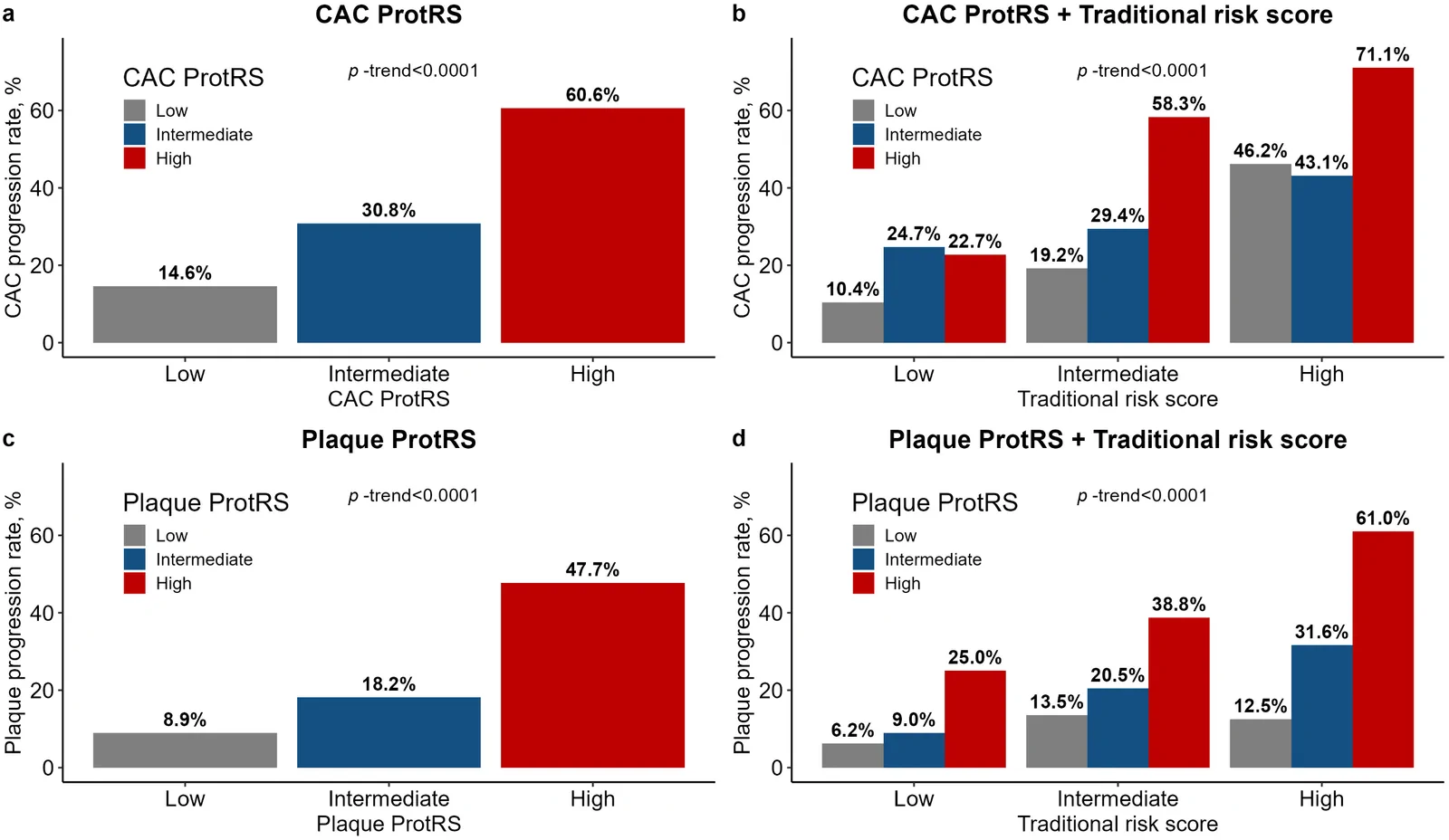
SA progression rates across ProtRS and traditional risk groups. **(a)** CAC progression rates across CAC ProtRS. **(b)** CAC progression rates across CAC ProtRS and traditional risk groups. **(c)** Plaque progression rates across plaque ProtRS. **(d)** Plaque progression rates across plaque ProtRS and traditional risk groups. ProtRS categories were defined based on percentiles: low-risk (bottom 20%), intermediate-risk (middle 60%), and high-risk (top 20%). The *p*_trend_ was calculated via a chi-squared test for trend in proportions. Abbreviations: SA, subclinical atherosclerosis; CAC, coronary artery calcium; ProtRS, protein risk score.

After adjusting for traditional risk factors, each SD increase in the CAC ProtRS was associated with an RR of 1.26 (95% CI [1.16,1.36]; *p* < 0.001) for CAC progression, and each SD increase in the plaque ProtRS yielded an RR of 1.25 (95% CI [1.18,1.33]; *p* < 0.001) for plaque progression. Compared to those in the bottom 20% ProtRSs group, participants in the intermediate and high CAC ProtRS risk group had RRs of 1.75 (95% CI [1.18,2.60]) and 2.44 (95% CI [1.58,3.77]) for CAC progression. Similarly, participants in the intermediate and high plaque ProtRS risk group had RRs of 1.74 (95% CI [1.01,2.97]) and 3.17 (95% CI [1.77,5.68]) for plaque progression (all *p*_trend_<0.001) (Table 2). SA ProtRSs quintiles also demonstrated a significant dose-response relationship with SA progression. Each quintile increase was associated with a 42% higher risk for CAC progression (RR 1.42, *p*_trend_ < 0.001) and 57% elevated risk for plaque progression (RR 1.57, *p*_trend_ < 0.001), respectively (Fig C in S1 Appendix).

**Table 2:** Associations of protein risk scores with SA progression and incident ASCVD.

Protein risk score	Outcome	Per SD increment	*P*-value	By protein risk category			
				Low-risk	Intermediate-risk	High-risk	*P*_trend_
CAC	CAC progression	1.26 (1.16–1.36)	<0.0001	1.00 (ref)	1.75 (1.18–2.60)	2.44 (1.58–3.77)	<0.0001
Plaque	Plaque progression	1.25 (1.18–1.33)	<0.0001	1.00 (ref)	1.74 (1.01–2.97)	3.17 (1.77–5.68)	<0.0001
CAC	ASCVD	1.36 (1.20–1.54)	<0.0001	1.00 (ref)	1.92 (1.25–2.94)	3.25 (2.02–5.24)	<0.0001
Plaque	ASCVD	1.38 (1.23–1.54)	<0.0001	1.00 (ref)	2.11 (1.37–3.25)	3.30 (2.05–5.31)	<0.0001

Data were presented as RR (95% CI) for SA progression, and HR (95% CI) for incident ASCVD. All models were adjusted for baseline age, sex, urbanization, smoking status, waist circumference, systolic blood pressure, antihypertensive therapy, total cholesterol, high-density lipoprotein cholesterol, and diabetes. For analyses in ASCVD case-cohort, adjustments additionally included cohort origin and geographic region. ProtRS categories were defined based on percentiles: low-risk (bottom 20%), intermediate-risk (middle 60%), and high-risk (top 20%). The *p*_trend_ was calculated by taking the medians of each protein risk category (low, intermediate, and high) as continuous numeric variables. *P-*values and *p*_trend_ were calculated using the Wald test. Abbreviations: SA, subclinical atherosclerosis; ASCVD, atherosclerotic cardiovascular disease; SD, standard deviation; RR, relative risk; CI, confidence interval; HR, hazard ratio; CAC, coronary artery calcium.

The ProtRSs had good discrimination of CAC and plaque progression (AUC: 0.711 (95% CI [0.673,0.749]) for CAC progression and 0.725 (95% CI [0.680,0.769]) for plaque progression) (Fig D in S1 Appendix). There was a significant improvement in the AUC after adding ProtRSs into traditional ASCVD risk factors, suggested by ΔAUC of 2.2% with DeLong’s *p* = 0.014 (adding CAC ProtRS) and ΔAUC of 3.6% with DeLong’s *p* < 0.001 (adding plaque ProtRS). The addition of ProtRSs could also significantly improve the model fit beyond traditional ASCVD risk factors for CAC and plaque progression (both *p* of likelihood ratio test < 0.001). Calibration plots demonstrated that models incorporating traditional ASCVD risk factors and ProtRSs exhibited overall acceptable calibration for predicting CAC and plaque progression (Fig E in S1 Appendix).

Sensitivity analyses showed that the improvement of ProtRSs in AUCs for CAC and plaque progression remained significant when adjusting for treated hyperlipidemia and treated diabetes in the base model or excluding participants with baseline ASCVD events (*n* = 34) (DeLong’s *p* < 0.05; Figs F and G in S1 Appendix).

### SA protein risk scores and 10-year ASCVD risk

The SA ProtRSs were significantly associated with ASCVD events, with adjusted HRs of 1.36 (95% CI [1.20,1.54], *p* < 0.001) per SD increase in the CAC ProtRS and 1.38 (95% CI [1.23,1.54], *p* < 0.001) per SD increase in the plaque ProtRS, respectively (Table 2). Compared with the low-risk group, participants in the intermediate and high CAC ProtRS risk group had HRs of 1.92 (95% CI [1.25,2.94]) and 3.25 (95% CI [2.02,5.24]) for ASCVD risk, and those in the intermediate and high plaque ProtRS risk group had HRs of 2.11 (95% CI [1.37,3.25]) and 3.30 (95% CI [2.05,5.31]) (both *p*_trend_ < 0.001). Both CAC and plaque ProtRS also exhibited significant linear associations, with each quintile increase corresponding to a 58% (HR 1.58, *p*_trend_ < 0.001) and a 59% (HR 1.59, *p*_trend_ < 0.001) elevated risk for ASCVD incidence, respectively (Fig H in S1 Appendix). The associations between the ProtRSs and ASCVD events were consistent across strata of age category, sex, hypertension, dyslipidemia, and diabetes (Table H in S2 Appendix). In particular, we observed a strong positive correlation between the strengths of effects on SA progression and ASCVD risk for proteins in SA ProtRSs (Pearson’s *r* 0.77, *p* = 0.001 for CAC; Pearson’s *r* 0.85, *p* < 0.001 for plaque progression, Fig 4).

**Fig 4:**
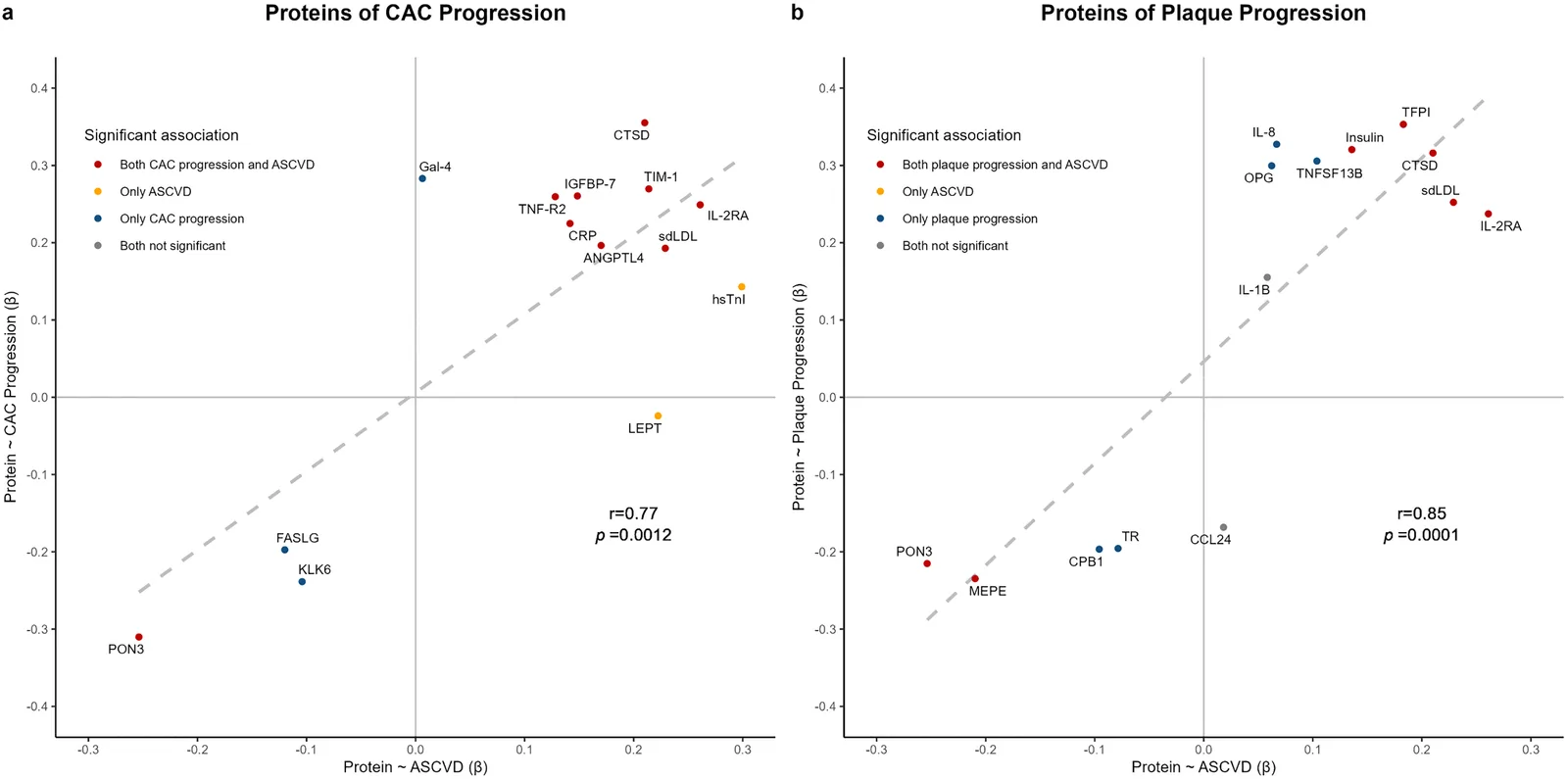
Comparison of effect sizes between SA progression and ASCVD incidence for proteins comprising ProtRSs. **(a)** CAC progression vs. incident ASCVD. **(b)** Plaque progression vs. incident ASCVD. The 14 proteins comprising the CAC ProtRS and the 14 proteins comprising the plaque ProtRS, which were selected by LASSO regression, are presented. Effect sizes for SA progression and ASCVD incidence were estimated using age- and sex-adjusted logistic regressions and Prentice Cox regressions adjusted for age, sex, and cohort origin, respectively. Points are colored by the significance of association with both SA progression and ASCVD incidence. Pearson’s correlation coefficient (r) with two-tailed *p* value and line of best fit are shown. Abbreviations: CAC, coronary artery calcium; SA, subclinical atherosclerosis; ASCVD, atherosclerotic cardiovascular diseases; LASSO, least absolute shrinkage and selection operator.

SA ProtRSs remained significantly associated with both CHD and IS. For CHD, each SD increment in CAC ProtRS and plaque ProtRS yielded HRs of 1.58 (95% CI [1.34,1.86], *p* < 0.001) and 1.59 (95% CI [1.38,1.82], *p* < 0.001), respectively (Table I in S2 Appendix). Of note, compared to those in the bottom 20% ProtRSs group, participants in the high CAC and plaque ProtRS risk group had HRs of 4.52 (95% CI [2.21,9.25], *p*_trend_ < 0.001) and 5.44 (95% CI [2.55,11.57], *p*_trend_ < 0.001). For IS, each SD increment in plaque ProtRS showed a significant association (HR 1.20, 95% CI [1.05,1.38]; *p* = 0.009).

Compared with the China-PAR model alone, the addition of ProtRSs significantly improved discriminative performance, with a ΔC-index of 0.018 (95% CI [0.003,0.035], *p* = 0.011). The overall C-index was elevated from 0.724 (95% CI [0.699,0.747]) to 0.742 (95% CI [0.717,0.766]) (Table 3). Furthermore, the incorporation of ProtRSs yielded an improvement in risk reclassification with NRI of 0.127 (*p* < 0.001) in all participants, consisting of 14.8% (*p* < 0.001) for ASCVD cases and −2.1% (*p* = 0.001) for noncases (Table J in S2 Appendix). Notably, among participants with intermediate-risk, the overall NRI for ProtRSs reached 0.416 (*p* < 0.001), with 16.0% (*p* = 0.009) and 25.6% (*p* = 0.008) of individuals net correctly reclassified for cases and noncases, respectively. Specifically, ProtRSs correctly upclassified 34.9% (37/106) of future ASCVD events from intermediate to high-risk, while 18.9% (20/106) of cases were erroneously downclassified from intermediate to low-risk.

**Table 3:** Prediction performance of protein risk scores in 10-year risk of incident ASCVD.

Models	Harrell’s C-index (95% CI)	Improvement of Harrell’s C-index (95% CI)	*p*-value for improvement of Harrell’s C-index
Reference score: Age + sex	0.693 (0.664–0.718)	Reference	Reference
SA ProtRSs	0.696 (0.668–0.721)	–	–
Age + sex + SA ProtRSs	0.728 (0.701–0.752)	0.035 (0.021–0.050)	<0.001
Reference score: China-PAR	0.724 (0.699–0.747)	Reference	Reference
China-PAR + SA ProtRSs	0.742 (0.717–0.766)	0.018 (0.003–0.035)	0.011

SA ProtRSs include both CAC and plaque ProtRSs. The sex-specific China-PAR risk score includes age, geographic region, urbanization, systolic blood pressure, antihypertensive therapy, total cholesterol, high-density lipoprotein cholesterol, waist circumference, current smoking, diabetes mellitus, and family history of ASCVD, as well as interactions of age with some variables. The 95% CI for Harrell’s C-index and 95% CI for improvement of Harrell’s C-index with one-sided *p*-value were estimated from bootstrapping (1,000 iterations). Abbreviations: ASCVD, atherosclerotic cardiovascular disease; CAC, coronary artery calcium; China-PAR, Prediction for Atherosclerotic cardiovascular disease Risk in China; CI, confidence interval; ProtRSs, protein risk scores; SA, subclinical atherosclerosis.

Integration of SA ProtRSs further revealed clear risk gradients across all conventional risk strata (all *p*_trend_ < 0.05) (Fig 5A). Specifically, 18.6% of individuals classified as traditional intermediate-risk and further identified as high-risk by ProtRSs demonstrated a 10-year ASCVD event rate of 10.9% (over the guideline-recommended high-risk threshold of 10%), supporting the initiation of targeted preventive interventions. In addition, participants simultaneously identified as high-risk by both the conventional risk score and SA ProtRS exhibited an even higher 10-year event rate of 13.7%, highlighting the necessity of intensified risk management for this subgroup (Fig 5B).

**Fig 5:**
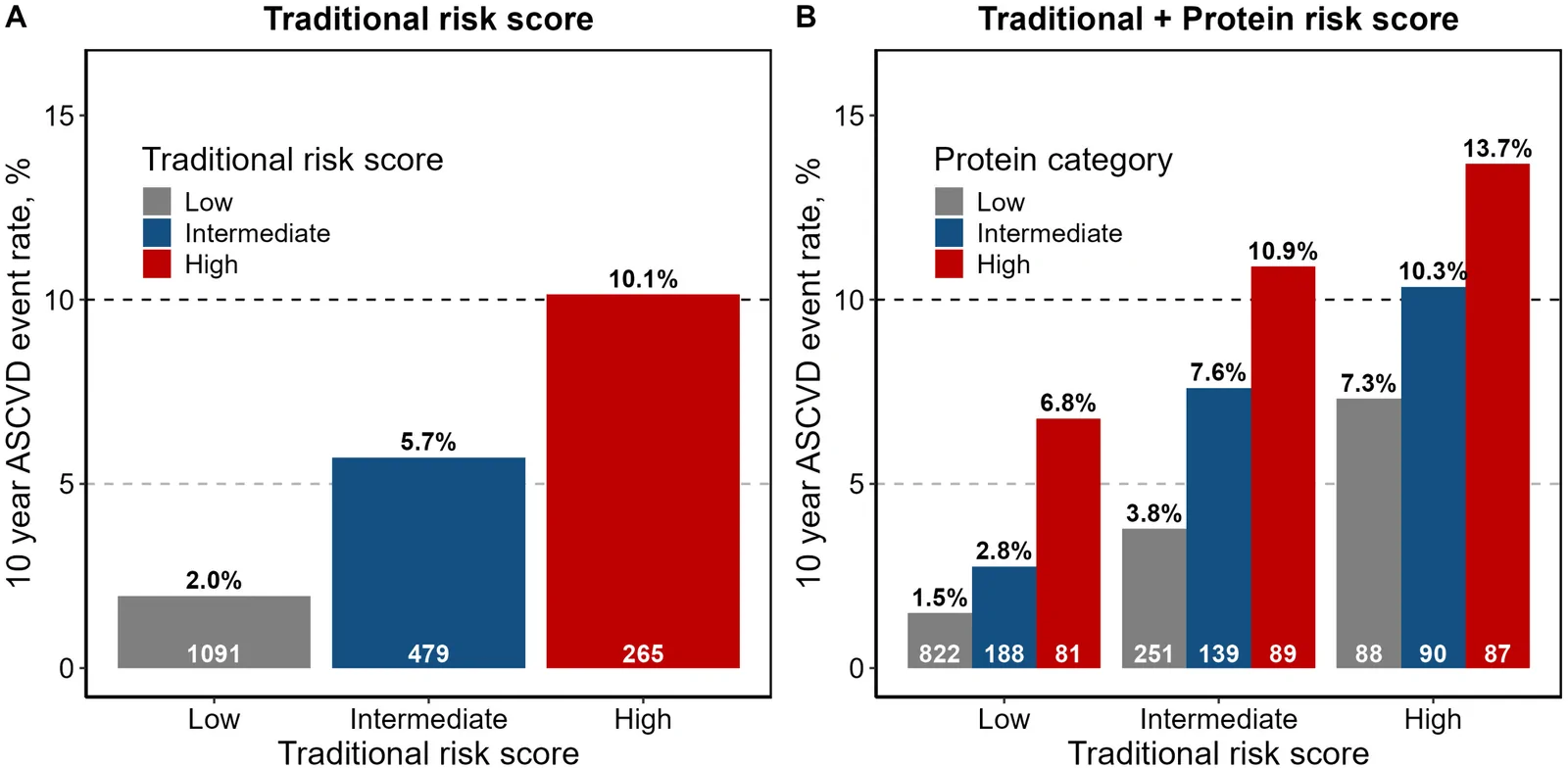
Ten-year risk of ASCVD stratified by traditional and protein risk categories. Bar plots show 10-year ASCVD risk stratification using traditional and protein risk categories. **(a)** Kaplan-Meier event rates by traditional risk categories: low (< 5%), intermediate (5%–10%), high (≥ 10%). **(b)** Kaplan–Meier event rates by combined risk stratification of traditional and protein risk categories. Protein risk scores include both CAC and plaque protein risk scores. ProtRS categories were defined as: high (high CAC and plaque ProtRS), intermediate (high CAC or plaque ProtRS), low (nonhigh ProtRS). Abbreviations: ASCVD, atherosclerotic cardiovascular diseases; ProtRS, protein risk score; CAC, coronary artery calcium.

## Discussion

Leveraging prospective cohorts with repeated measurements of CAC and carotid plaque, we characterized plasma protein profiles underlying subclinical atherosclerotic progression. The derived ProtRSs demonstrated robust linear associations with incident ASCVD and conferred modest incremental gains in risk stratification beyond conventional guideline-based risk assessment. In particular, reclassification improvements were most pronounced in guideline-defined intermediate-risk individuals who may benefit greatly from early intervention. Our findings advance the understanding of plasma proteins for ASCVD primary prevention through three key aspects.

First, we systematically selected protein drivers underlying SA progression, thereby elucidating the biological processes underlying CAC and carotid plaque. Unlike previous cross-sectional investigations [11,28–30], our prospective study with a median 6-year follow-up enabled identification of proteins that precede and potentially mediate atherosclerotic progression. Furthermore, forward stepwise logistic regression analyses verified that most LASSO-selected proteins might be biologically relevant to SA progression. We observed shared proteins implicated in both CAC and plaque progression (CTSD, sdLDL, and PON3), which were involved in well-established atherosclerotic mechanisms, including autophagy-related inflammation, lipid deposition, metabolic regulation, and immune modulation [31–37]. Meanwhile, KEGG pathway analysis further demonstrated that protein signatures for plaque progression and CAC progression converged on inflammation and cholesterol homeostasis regulation pathways that drive atherogenesis, including IL-8, CCL-24, MCP-1, and low-density lipoprotein receptor [38–41].

Moreover, we uncovered multiple previously underrecognized protein biomarkers involved in atherogenesis. KLK6 displayed protective associations with CAC progression, consistent with its established neuroprotective properties [42]. Transferrin receptor protein 1 (TR), a key regulator of ferroptosis, was also identified as an important protein involved in pathologic processes of atherosclerosis [43]. Additionally, osteoprotegerin (OPG) and CPB1 were associated with plaque progression, further highlighting the roles of vascular calcification and proteolytic remodeling in atherosclerotic pathogenesis [44,45]. A previous phenome-wide mendelian randomization study has clarified the causal relationships of these proteins with cardiovascular disease-related traits, including KLK6 with body mass index, CPB1 with myocardial infarction, as well as TR with low-density lipoprotein cholesterol and hemoglobin A1c (Table K in S2 Appendix and S1 Text) [46].

Second, we developed CAC- and plaque-specific ProtRSs that demonstrated robust associations with subclinical SA progression and subsequent incident ASCVD. In our cohort with a mean age of 54 years, CAC progression was observed in 33.4% of participants and carotid plaque progression in 22.0%, underscoring the clinical need for sensitive biomarkers to identify individuals prone to progressive vascular disease. The constructed ProtRSs explained nearly 20% of the phenotypic variance in SA progression and corresponded to a 1.25-fold elevated risk of progressive atherosclerosis.

Most prior protein-based prediction studies exclusively focused on overt cardiovascular events, with insufficient attention paid to early subclinical vascular remodeling [47]. Furthermore, conventional cardiovascular biomarkers, such as N-terminal prohormone brain natriuretic peptide (NT-proBNP), apolipoprotein(a) (LPA) and proprotein convertase subtilisin/kexin type 9 (PCSK9), showed no significant associations with SA progression in our analyses. Accordingly, our study addresses this prominent knowledge gap by establishing atherosclerosis-tailored risk scores, which are uniquely valuable for monitoring early vascular disease progression. Of critical importance, proteins displaying stronger associations with subclinical atherosclerotic progression also exhibited more pronounced links to incident ASCVD, with high phenotypic coherence (CAC progression: *r* 0.77, *p* = 0.001; plaque progression: *r* 0.85, *p* < 0.001). This biological consistency supports a plausible causal chain whereby protein-mediated alterations in early atherosclerosis ultimately translate to future clinical cardiovascular events, in line with analogous mechanistic concordance reported in prior biomarker studies [48,49]. In line with this framework, each SD increase in ProtRSs was associated with over 35% higher ASCVD risk. Individuals with high ProtRS risk presented a 3-fold higher incidence of ASCVD relative to those with low ProtRS risk, and those with high ProtRS risk demonstrated a 4- to 5-fold elevated risk of CHD compared with their low-risk counterparts. Collectively, these findings establish a biologically coherent link between protein-driven subclinical atherosclerotic progression and long-term adverse cardiovascular outcomes.

Third, the ProtRSs could confer incremental value for ASCVD risk prediction and reclassification beyond conventional risk assessment. Previous evidence reported that 10%, 33%, and 55% of individuals categorized as low-, intermediate-, and high-risk by traditional risk algorithms exhibited generalized SA, respectively [50]. This highlights the inherent limitations of conventional risk scores in identifying subclinical atherosclerotic burden, leaving considerable residual cardiovascular risk unaddressed in primary ASCVD prevention [51,52]. Previous proteomic studies have explored the utility of circulating protein biomarkers and found modest improvement when adding plasma proteins to traditional risk scores in primary prevention settings [53,54]. For instance, in a UK cohort comprising 38,380 participants with 2,919 proteins quantified, the addition of 114 selected proteins to Systematic COronary Risk Evaluation 2 (SCORE2) yielded mild improvements in 10-year major cardiovascular events (MACE) risk prediction (AUC 0.771; NRI 0.140) [54]. In comparison, our analysis revealed an overall significant reclassification improvement with a total NRI of 0.127, reflecting appropriate reallocation of participants into clinically meaningful risk strata. Notably, the reclassification benefit was markedly pronounced among guideline-defined intermediate-risk individuals, with an NRI of 0.416. However, adding ProtRSs also incorrectly reclassified 20 of 106 future cases from intermediate to low-risk, potentially leading to missed preventive opportunities.

Absolute risk stratification further revealed that intermediate-risk individuals reclassified to high-risk by the ProtRS exhibited a 10-year ASCVD incidence of 10.9%, surpassing the guideline-recommended threshold for initiating pharmacological intervention. Accordingly, the ProtRS may act as a valuable risk enhancer to guide clinical decision-making among individuals with ambiguous baseline risk under current guidelines. Furthermore, within the conventionally high-risk population, the ProtRS identified a high-risk subgroup with an elevated 10-year event rate of 13.7%, supporting the need for more aggressive preventive strategies, particularly among Chinese populations characterized by relatively poor adherence to cardiovascular interventions [55,56]. Taken together, these findings indicate that integrating ProtRSs into traditional risk scores can refine risk stratification and identify individuals with SA who may benefit from earlier intervention.

Overall, our study adds new insights into the limited evidence regarding proteins contributing to SA progression. The CAC- and plaque-specific protein risk scores demonstrated robust predictive capacity for SA progression, and their associations with incident ASCVD further support the clinical relevance of these proteomic markers in longitudinal follow-up. However, our findings should be interpreted in the context of certain limitations. First, despite the relatively broad 117-protein panel adopted, it may not encompass the full spectrum of the atherosclerotic proteome. Second, although pathway enrichment analysis supported the protein-disease associations, the causal biological mechanisms require functional validation through mechanistic investigations. Third, further large-scale external validation across populations and cost-effectiveness evaluation are needed before these protein risk scores can be implemented in clinical practice.

In conclusion, the atherosclerosis-specific protein risk scores reflecting coronary calcification and carotid plaque progression confer incremental ASCVD predictive value over conventional risk assessment tools. These scores facilitate targeted early intervention for SA, with particularly prominent clinical benefits among guideline-classified intermediate-risk individuals.

## Supporting information

S1 Checklist STROBE statement – checklist of items that should be included in reports of cohort studies. Based on the STROBE Statement (Strengthening the Reporting of Observational Studies in Epidemiology). Available from: https://www.strobe-statement.org/. The STROBE Statement is distributed under the Creative Commons Attribution 4.0 International License (CC BY 4.0).
(DOCX)

S2 Checklist TRIPOD checklist: prediction model development and validation. Based on the TRIPOD Statement (Transparent Reporting of a multivariable prediction model for Individual Prognosis Or Diagnosis). Available from: https://www.tripod-statement.org/. The TRIPOD Statement is distributed under the Creative Commons Attribution 4.0 International License (CC BY 4.0).
(DOCX)

S1 Code Coding for statistical analyses is provided. (R)

S1 Data The minimal data set of the study. (XLSX)

S1 Text Supplementary methods. (DOCX)

S1 Appendix Figs A–H. **Fig A.** Flowchart of study participant selection for analyses. **Fig B.** Principal component analysis of 117 plasma proteins measured across three platforms. **Fig C.** The associations between ProtRS quintile groups and SA progression. **Fig D.** Receiver-operating characteristics curve for the predictive performance of the ProtRS combined with traditional risk factors for SA progression. **Fig E.** Calibration plots for CAC and plaque progression predictive models. **Fig F.** Receiver-operating characteristics curve for the predictive performance of the ProtRS combined with traditional risk factors for SA progression (included medications). **Fig G.** Receiver-operating characteristics curve for the predictive performance of the ProtRS combined with traditional risk factors for SA progression (excluded participants with ASCVD at baseline). **Fig H.** The associations between ProtRS quintile groups and incident ASCVD.
(DOCX)

S2 Appendix Tables A–K. **Table A.** Information of 117 proteins. **Table B.** Baseline characteristics of Beijing Community-Based Cohort of Atherosclerosis Study. **Table C.** Baseline characteristics of the full cohort and randomly sampled sub-cohort in the ASCVD case-cohort. **Table D.** LASSO effect sizes and selected chances of 117 proteins for subclinical atherosclerosis progression. **Table E.** Associations between proteins and subclinical atherosclerosis progression. **Table F.** KEGG pathways for 36 proteins within the optimal panel for subclinical atherosclerosis progression. **Table G.** Formulas for the SA ProtRSs. **Table H.** Subgroup analyses of the associations between ProtRSs and incident ASCVD. **Table I.** Associations of protein risk scores with incident coronary heart disease and ischemic stroke. **Table J.** Reclassification of risk categories of ASCVD comparing traditional risk scores with the addition of protein risk scores. **Table K.** Causal relationships of proteins with cardiovascular disease-related traits by phenome-wide mendelian randomization.
(XLSX)

## Abbreviations

ASCVD: atherosclerotic cardiovascular disease
CAC: coronary artery calcium
CHD: coronary heart disease
ChinaMUCA: China Multi-Center Collaborative Study of Cardiovascular Epidemiology
China-PAR: Prediction for Atherosclerotic Cardiovascular Disease Risk in China
CIs: confidence intervals
CPB1: carboxypeptidase B
CT: computed tomography
CTSD: cathepsin D
CVD: cardiovascular disease
FDR: false discovery rate
HRs: hazard ratios
InterASIA: International Collaborative Study of Cardiovascular Disease in Asia
IS: ischemic stroke
KEGG: Kyoto Encyclopedia of Genes and Genomes
LASSO: least absolute shrinkage and selection operator
MACE: major cardiovascular events
MESA: Multi-Ethnic Study of Atherosclerosis
NRI: net reclassification improvement
ORA: Over-Representation Analysis
PCSK9: proprotein convertase subtilisin/kexin type 9
PON3: paraoxonase 3
ProtRSs: protein risk scores
Q1: quartile 1
Q3: quartile 3
ROC: receiver-operating characteristic
RRs: relative risks
SA: subclinical atherosclerosis
SCORE2: Systematic COronary Risk Evaluation 2
SD: standard deviation
sdLDL: small dense low-density lipoprotein
TR: Transferrin receptor protein 1
uPA: urokinase-type plasminogen activator.
