# Supplementary material for: Plasma proteomic signatures of subclinical atherosclerosis progression for cardiovascular risk stratification: A cohort study
Source: PLoS Med. 2026 Oct 20;23(10):e1005265. doi: 10.1371/journal.pmed.1005265 (PMC13682661; doi:10.1371/journal.pmed.1005265)
Supplement: Supplementary file 3 [file pmed.1005265.s005.docx]

**Supplemental Text**

**Plasma proteomic signatures of subclinical atherosclerosis progression for cardiovascular risk stratification: A cohort study**

**S1 Text**

**Original study analysis plan**

***Study protocol of cohorts***

Participants of the Beijing Community-Based Cohort of Atherosclerosis Study were residents of Beijing and derived from the Prediction for Atherosclerotic Cardiovascular Disease Risk in China (China-PAR) project, and the follow-up visits and subclinical atherosclerosis (SA) examinations were performed in 2007–2008 and 2013–2014. Carotid ultrasound was conducted using an iE33 ultrasonography (Philips Healthcare, Netherland) with 7.5-MHz linear-array transducers by experienced sonographers, according to a standardized scanning protocol. A plaque was defined as any focal protrusion of a carotid intima-media thickness (cIMT) value ≥ 1.5 mm or > 50% thicker than the adjacent cIMT by visual assessment. Carotid plaque score was determined by the total number of plaques detected in each segment of bilateral carotid artery, including common carotid artery, the carotid bifurcation, and the internal carotid artery. CAC was measured with a 64-row LightSpeed VCT scanner (GE Healthcare, Milwaukee, WI, USA). CAC score (CACS) was calculated using the Agatston method for each major epicardial vessel (left main, left anterior descending, left circumflex and right coronary artery), and total CACS was calculated as the sum of CACS of four epicardial vessels.

International Collaborative Study of Cardiovascular Disease in Asia (InterASIA), which was established from 2000 to 2001, selected a nationally representative sample 35 to 74 years of age in China using a 4-stage stratified sampling method. The sampling process was stratified by geographic region (northern/southern China, divided by the Yangtzi River) and urbanization (urban/rural). The China Multi-Center Collaborative Study of Cardiovascular Epidemiology (ChinaMUCA) was established in 1998 with cluster random sampling. Fifteen clusters were selected that also were based on geographic region and urbanization, with ≈500 men and 500 women 35 to 59 years of age in each cluster. The first follow-up survey for both InterASIA and the ChinaMUCA was conducted during 2007 to 2008, and the second and the third was conducted during 2012 to 2015, and 2018 to 2021.

***Blood sample collection***

Blood samples were collected by professionally trained technical staff. Instructions for ≥10 hours of overnight fasting were provided to the participants by the investigators on the day prior to blood collection. Disposable vacuum blood collection tubes were used for sampling, including tubes containing ethylenediaminetetraacetic acid (EDTA) as an anticoagulant and plain serum tubes. Immediately after collection, the EDTA tubes were inverted 5–6 times to ensure adequate mixing with the anticoagulant. All blood samples were centrifuged within 2 hours of collection (2000g, 4℃, 10 minutes). After plasma separation, the samples were aliquoted into 1.5mL EP tubes and stored in a -80℃ freezer. All biospecimens were transported using a cold chain process and were centrally stored in the biobank at Fuwai Hospital, Chinese Academy of Medical Sciences.

***Outcome diagnosis criteria***

During follow-up duration, study participants or their proxies were identified and interviewed to obtain disease status and vital information. Hospital records or death certificates were also collected. A study-wide end-point assessment committee at Fuwai Hospital in Beijing adjudicated the final end-point events by reviewing all incidence and death records. Two committee members independently verified the events, and discrepancies were resolved by discussion involving additional committee members. Acute myocardial infarction was identified as a change in biochemical markers of myocardial necrosis accompanied by ischemic symptoms, pathological Q waves, ST-segment elevation or depression, or coronary intervention. Fatal events resulting from myocardial infarction or other coronary deaths were defined as coronary heart disease death. Ischemic stroke included clinical signs and symptoms of cerebral infarction, which were rapidly developing signs of focal (or global) disturbances in cerebral function lasting >24 hours without an apparent nonvascular cause.

***Measurement of covariates***

Baseline information on social demographic characteristics, lifestyle risk factors, medication, disease condition and family history of ASCVD was collected by trained staff using standard questionnaires according to uniform protocols. Alcohol consumption habit during the past year was assessed and individuals were asked whether they were drinkers (alcohol consumption ≥12 time/year) or non-drinkers (alcohol consumption <12 time/year). Participants underwent physical examinations. Waist circumference (WC) was measured at 1 cm above the navel at minimum breath. BP was measured using a standard electronic BP monitor after at least 5-min rest in a seated position. Three BP measurements with a 30-second interval between each measurement were collected, the average of which were used for analysis. Participants provided fasting blood samples (fasting for at least 10h) for the assessment of glucose and cholesterol concentrations. Missing covariate data were imputed using the ‘mice’ package in R with the classification and regression trees (cart) method.

***Proteomics measurements***

Protein measurement was performed using three platforms: the Olink Cardiovascular-III panel using proximity extension assay technology for 92 proteins, an automated ELISA-based microfluidic system (ELLA, ProteinSimple) for 14 inflammatory proteins, and clinical biochemistry assays on Beckman Coulter AU680 for 11 metabolic proteins. The average proportion of missing values across proteins was less than 1%. Protein values outside the limits of detection were winsorized: values below the lower limit of detection (LOD) were set to one-third of the lower LOD, and values above the upper LOD were set to four-thirds of the upper LOD.

***Protein risk score***

All measured concentrations underwent Z-score standardization (mean=0, standard deviation [SD]=1). LASSO regression with 10-fold cross-validation was performed 1000 times (randomly selected 90% sample size) to select proteins associated with CAC or plaque progression, adjusting for age and sex. Proteins retaining non-zero coefficients in ≥900 iterations for each outcome were subsequently incorporated into two distinct ProtRSs through logistic regression modeling. Final ProtRS values were calculated on the logit scale as the sum of each protein’s regression coefficient multiplied by its standardized plasma level.

***Association analysis***

Log-binomial regressions were used to estimate relative risks (RRs) and 95% confidence intervals (CIs) of ProtRSs for CAC and plaque progression, respectively, adjusting for traditional cardiovascular disease (CVD) risk factors including baseline age, sex, urbanization, smoking status, waist circumference, systolic blood pressure, antihypertensive therapy, total cholesterol, high-density lipoprotein cholesterol, and diabetes. To evaluate the predictive accuracy of SA ProtRSs for the risk of SA progression, area under the receiver operating characteristic (ROC) curve (AUC) values were estimated for each ProtRS, and their comparisons with a reference model including age and sex were assessed. For the risk of incident ASCVD, Prentice Cox regressions were used to estimate hazard ratios (HRs) and 95% CIs of ProtRSs in the case-cohort.

***Prediction evaluation***

The incremental value of ProtRSs was assessed over the guideline-recommended Prediction for Atherosclerotic cardiovascular disease Risk in China (China-PAR) risk score, which is a sex-specific risk equation for the 10-year risk of ASCVD. Due to the absence of hemorrhagic stroke in the current study outcome compared with the original China-PAR risk score, when calculating the absolute observed ASCVD event rate, the baseline survival function (S0) was re-estimated among the entire cohort (n=14,496) and thresholds of <4%, 4-8%, and ≥8% were set to define China-PAR low-, intermediate-, and high-risk groups. The prediction performance of ProtRSs and their added value to the reference risk score were evaluated by Harrell’s C-index through 1000 bootstrapping iterations. In addition, categorical net reclassification improvement (NRI) was estimated based on the cut-off value of 8%. Furthermore, risk stratification achieved by SA ProtRSs beyond the China-PAR risk score was evaluated within a 10-year timeframe by estimating the group-specific observed risks. The SA protein risk category was combined as high (high CAC and high plaque ProtRS), intermediate (only high CAC or only high plaque ProtRS), and low (low or intermediate CAC and low or intermediate plaque ProtRS). The observed risk of incident ASCVD for each category was estimated using the Kaplan-Meier method and a weighting of 1/ (sampling proportion) was applied to the sub-cohort individuals to compensate for the over-representation of cases.

***Phenome-wide PheWAS Browser***

URL available from: https://www.epigraphdb.org/pqtl/ [accessed: 25/06/2025]

Reference: Zheng J, Haberland V, Baird D, Walker V, Haycock PC, Hurle MR, et al. Phenome-wide Mendelian randomization mapping the influence of the plasma proteome on complex diseases. Nature Genetics. 2020;52(10):1122-31. <http://doi.org/10.1038/s41588-020-0682-6>.

**Changes to the** **statistical analysis in response to reviewers**

Following the reviewers’ suggestions, we revised the statistical analysis as follows:

1. **Supplemental analysis:** We added Delong’s *p*, the likelihood ratio test, calibration plots, and several sensitivity analyses to assess improvements in model discrimination, overall fit, and agreement between the observed and predicted risks for predicting SA progression.
2. **Predictive performance of SA ProtRSs:** In the original analysis, we estimated the AUC for each score and compared it with age and sex alone. In the revised analysis, we additionally included a reference model incorporating traditional CVD risk factors to present the additive predictive value of ProtRSs for SA progression.
3. **Detection of troponin:** we newly detected plasma troponin levels in all study participants and further constructed an updated ProtRSs incorporating troponin to enhance the predictive performance in incident ASCVD.
4. **China-PAR risk categories:** Although the China-PAR risk score was re-estimated due to the exclusion of hemorrhagic stroke in the current study, we now retained the original thresholds of <5%, 5-10%, and ≥10% to define low-, intermediate-, and high-risk groups to facilitate interpretation.
5. **Net reclassification improvement:** In accordance with case-cohort design guideline suggested by reviewers, we recalculated NRI in a corrected method: cases that developed outside the sub-cohort after 10 years were excluded from NRI calculations. To further explore the clinical implications, NRI was also calculated among participants with intermediate China-PAR risk (5 to 10%). And we added the *p*-value of categorical NRI which obtained through 1000 bootstrapping iterations.
