# Supplementary material for: Plasma proteomic signatures of subclinical atherosclerosis progression for cardiovascular risk stratification: A cohort study
Source: PLoS Med. 2026 Oct 20;23(10):e1005265. doi: 10.1371/journal.pmed.1005265 (PMC13682661; doi:10.1371/journal.pmed.1005265)
Supplement: Supplementary file 4 [file pmed.1005265.s006.docx]

# Fig A. Flowchart of study participant selection for analyses.

Abbreviation: SA, subclinical atherosclerosis; ASCVD, atherosclerotic cardiovascular diseases; InterASIA, International Collaborative Study of Cardiovascular Disease in Asia; ChinaMUCA, China Multi-Center Collaborative Study of Cardiovascular Epidemiology; CAC, coronary artery calcium.

# Fig B. Principal component analysis of 117 plasma proteins measured across three platforms.

(A) Plasma proteins measured in the Atherosclerosis Cohort. (B) Plasma proteins measured in the ASCVD case-cohort. Abbreviation: PC, principal component.

# Fig C. The associations between ProtRS quintile groups and SA progression.

(A) Association of CAC ProtRS quintile groups with CAC progression. (B) Association of plaque ProtRS quintile groups with plaque progression. The diagonal dashed lines represent linear models fitted based on the relative risk trend of the groups, and the RR values are calculated from the slopes of linear models: RR=exp(slope). The *p*_trend_ was calculated by taking each protein risk categories (by quintiles) as continuous numeric variables using the Wald test. The horizontal dashed lines represent RR=1. Abbreviations: CAC, coronary artery calcium; ProtRS, protein risk score; RR, relative risk; CI, confidence interval; SA, subclinical atherosclerosis.

# Fig D. Receiver-operating characteristics curve for the predictive performance of the ProtRS combined with traditional risk factors for SA progression.

(A) Prediction of CAC progression. (B) Prediction of plaque progression. The risk factors consist of baseline age, sex, urbanization, smoking status, waist circumference, systolic blood pressure, antihypertensive therapy, total cholesterol, high-density lipoprotein cholesterol, and diabetes. The area under the curve (AUC) with its 95% confidence interval is presented in parentheses and incremental AUC improvements of risk factors with addition of protein risk scores to the risk factor model were assessed using DeLong’s test. Abbreviations: CAC, coronary artery calcium; ProtRS, protein risk score; SA, subclinical atherosclerosis.

# Fig E. Calibration plots for CAC and plaque progression predictive models.

(A) Calibration plot for CAC progression. (B) Calibration plot for plaque progression. The predicted risk was calculated by the models combining risk factors (including baseline age, sex, urbanization, smoking status, waist circumference, systolic blood pressure, antihypertensive therapy, total cholesterol, high-density lipoprotein cholesterol, and diabetes) and protein risk scores. The predicted risk was divided into deciles, and 95%CIs of observed risk were presented.

# Fig F. Receiver-operating characteristics curve for the predictive performance of the ProtRS combined with traditional risk factors for SA progression (included medications).

(A) Prediction of CAC progression. (B) Prediction of plaque progression. The risk factors consist of baseline age, sex, urbanization, smoking status, waist circumference, systolic blood pressure, antihypertensive therapy, total cholesterol, high-density lipoprotein cholesterol, diabetes, treated hyperlipidemia, and treated diabetes. The area under the curve (AUC) with its 95% confidence interval is presented in parentheses and incremental AUC improvements of risk factors with addition of protein risk scores to the risk factor model were assessed using DeLong’s test. Abbreviations: CAC, coronary artery calcium; ProtRS, protein risk score; SA, subclinical atherosclerosis.

# Fig G. Receiver-operating characteristics curve for the predictive performance of the ProtRS combined with traditional risk factors for SA progression (excluded participants with ASCVD at baseline).

(A) Prediction of CAC progression. (B) Prediction of plaque progression. The risk factors consist of baseline age, sex, urbanization, smoking status, waist circumference, systolic blood pressure, antihypertensive therapy, total cholesterol, high-density lipoprotein cholesterol, and diabetes. The area under the curve (AUC) with its 95% confidence interval is presented in parentheses and incremental AUC improvements of risk factors with addition of protein risk scores to the risk factor model were assessed using DeLong’s test. Abbreviations: CAC, coronary artery calcium; ProtRS, protein risk score; SA, subclinical atherosclerosis.

# Fig H. The associations between ProtRS quintile groups and incident ASCVD.

(A) Association of CAC ProtRS with incident ASCVD. (B) Association of plaque ProtRS with incident ASCVD. The cut-off points of ProtRS groups are from sub-cohort. The diagonal dashed lines represent linear models fitted based on the hazard risk trend of the groups, and the HR values are calculated from the slopes of linear models: HR=exp(slope). The *p*_trend_ was calculated by taking each protein risk categories (by quintiles) as continuous numeric variables using the Wald test. The horizontal dashed lines represent HR=1. Abbreviations: CAC, coronary artery calcium; ProtRS, protein risk score; ASCVD, atherosclerotic cardiovascular disease; HR, hazard ratio; CI, confidence interval.
