# Supplementary material for: Plasma proteomic signatures of subclinical atherosclerosis progression for cardiovascular risk stratification: A cohort study
Source: PLoS Med. 2026 Oct 20;23(10):e1005265. doi: 10.1371/journal.pmed.1005265 (PMC13682661; doi:10.1371/journal.pmed.1005265)
Supplement: Supplementary file 6 [file pmed.1005265.s009.docx]

**Reviewers' comments:**

**Reviewer #1:**

1. *Thanks for the opportunity to read your manuscript. My role is statistical reviewer, so I have focused on the design, data, and analysis that are presented. I have put general comments first, followed by questions relevant to a specific section of the manuscript (with a page/line reference).*

*This manuscript proposes and tests the addition of proteomic data in predicting risk of subclinical atherosclerosis. The study had two parts, an initial 'discovery' phase which used data from cohort of patients examined with CU and CCT, and with protein data, along with self-reported information (demographic and health). Risk score for CAC and plaque progression was estimated with LASSO regression with CV, with a variable retained if it had a non-zero coefficient in >90% of the CV samples. These scores were then categorised in the low, medium, and high risk, and the discrimination for SA progression was assessed with AUC.*

*The second phase used data from a large ongoing cohort study with protein and outcome data, in a case-control design. All incidence cases of AVCD and a random sample of non-cases (10%) were used from this study. The incremental value of the protein risk scores over a routinely used risk score for ASCVD was assessed with a weighted C-index and NRI.*

**Response:** We appreciate the careful review. We have addressed all concerns in a point-by-point manner, and we believe that the manuscript has been substantially improved after revision.

2. *There were modest improvements in using the protein risk scores in the two phases. In the second phase, there was evidence of more accurate reclassification. How would an NRI value of 0.049 be interpreted? Is this a relatively large, or useful improvement?*

**Response:** The NRI aims to assess whether the addition of variables to the risk prediction model leads to an improvement in classification of participants into clinically relevant risk categories (*JACC Cardiovasc Imaging. 2015 Nov;8(11):1285-93; JAMA. 2026 Jan 20;335(3):245-254*). The previous NRI value was calculated by dividing individuals into two categories (low risk and high risk). In order to clarify the clinical interpretation of NRI, especially for individuals with intermediate risk, which needs special attention in clinical practice (*JAMA. 2012 Aug 22;308(8):788-95*), we recalculated NRI by dividing the risk into three categories (low risk, intermediate risk, and high risk) based on Review 4’s suggestions. Accordingly, we obtained a new NRI value of 12.67%, and a NRI of 41.62% for participants with intermediate risk.

The NRI value of 12.67% means that the addition of protein risk scores to the China-PAR risk prediction model leads to 12.67% participants correctly reclassified into clinically relevant risk categories, which is a useful improvement in risk prediction. Specifically, the NRI in participants with intermediate risk of 41.62% indicated that 41.62% participants with intermediate risk were correctly reclassified into lower or higher risk categories with addition of protein risk scores. Therefore, the implementation of protein risk scores can offer useful improvement on the risk classification of China-PAR risk prediction model and guide the prevention strategies of individuals with intermediate risk.

**Revision:** We provided a Supplementary table 10 to describe the reclassification of NRI values and also interpreted NRI in the Results and Discussion section (Page 13, line 330-337 and Page 15, line 416-421).

**Supplemental Table 10. Reclassification of risk categories of ASCVD comparing traditional risk scores with addition of protein risk scores.**

| Risk categories based on traditional risk score | Risk categories with addition of ProtRSs | | | |
| --- | --- | --- | --- | --- |
|  | Low risk | Intermediate risk | High risk | Total |
| *No. of all participants* |  |  |  |  |
| Low risk | 1298 | 128 | 24 | 1450 |
| Intermediate risk | 63 | 110 | 52 | 235 |
| High risk | 1 | 20 | 80 | 101 |
| Total | 1362 | 258 | 166 | 1786 |
| Increased risk |  |  |  | 204 |
| Decreased risk |  |  |  | 84 |
| NRI |  |  |  | 12.67% (*P*<0.001) |
| NRI in participants with intermediate risk |  |  |  | 41.62% (*P*<0.001) |
| *No. of participants experiencing an event within 10 years* | | | | |
| Low risk | 226 | 56 | 7 | 289 |
| Intermediate risk | 20 | 49 | 37 | 106 |
| High risk | 1 | 12 | 45 | 58 |
| total | 247 | 117 | 89 | 453 |
| Increased risk |  |  |  | 100 |
| Decreased risk |  |  |  | 33 |
| Net correctly reclassified (%) |  |  |  | 14.79% (*P*<0.001) |
| Net correctly reclassified in participants with intermediate risk (%) |  |  |  | 16.04% (*P*=0.009) |
| *No. of participants not experiencing an event when followed up for ≥ 10 years* | | | | |
| Low risk | 741 | 37 | 8 | 786 |
| Intermediate risk | 35 | 38 | 13 | 86 |
| High risk | 0 | 4 | 20 | 24 |
| Total | 776 | 79 | 41 | 896 |
| Increased risk |  |  |  | 58 |
| Decreased risk |  |  |  | 39 |
| Net correctly reclassified (%) |  |  |  | -2.12% (*P*=0.001) |
| Net correctly reclassified in participants with intermediate risk (%) |  |  |  | 25.58% (*P*=0.008) |

Notes: To calculate the net reclassification improvement of ten-year risk of ASCVD in case-cohort design, we excluded the cases that developed ASCVD after 10 years, and participants developed ASCVD after 10 years in sub-cohort were considered as non-cases. The traditional risk score was the sex-specific China-PAR risk score includes age, geographic region, urbanization (only for men), systolic blood pressure, antihypertensive therapy, total cholesterol, high-density lipoprotein cholesterol, waist circumference, current smoking, diabetes mellitus and family history of ASCVD (only for men), as well as interactions of age with some variables. ProtRSs include both CAC and plaque ProtRSs. Risk categories were defined as low risk (<5%), intermediate risk (5% to 10%), and high risk (>=10%). Abbreviations: ASCVD, atherosclerotic cardiovascular disease; CAC, coronary artery calcium; China-PAR, Prediction for Atherosclerotic cardiovascular disease Risk in China; ProtRSs, protein risk scores; NRI, net reclassification improvement.

3. *There are measures of discrimination provided (AUC), is it possible to see calibration plots to confirm consistency in the estimate of absolute risk?*

**Response:** According to your suggestion, we calculated the calibration-in-the-large, calibration slope (defined as the intercept and slope of a logistic regression line of outcome versus predicted risk), and calibration χ^2^ and *P*-value based on Hosmer-Lemeshow goodness of fit method. The calibration slope for both CAC and plaque progression were approximately equal to 1 with calibration-in-the-large close to zero (< 0.0001). We also observed consistent results by deciles of predicted risks (*P*>0.05), which indicated no significant difference between the observed risks and predicted risks of CAC progression and plaque progression by the risk factors with addition of ProtRSs.

**Revision:** We provided a Supplementary Figure 6 of calibration plots, and the methods and results were described in the manuscript (Page 8, line 191-194 and Page 12, line 296-299).

**Supplemental Figure 6. Calibration plots for CAC and plaque progression predictive models.**

Figure legends: (A) Calibration plot for CAC progression. (B) Calibration plot for plaques progression. The predicted risk was calculated by the models combining risk factors (including baseline age, sex, urbanization, smoking status, waist circumference, systolic blood pressure, antihypertensive therapy, total cholesterol, high-density lipoprotein cholesterol, and diabetes) and protein risk scores. The predicted risk was divided into deciles, and 95%CIs of observed risk were presented. The calibration χ2 and *P*-value was calculated based on Hosmer-Lemeshow goodness of fit method.

4. *Page 7, L126. Is this definition of CAC progression commonly used, or was it derived just for this study?*

**Response:** The definition of CAC progression used in our manuscript was commonly used instead of being derived for our study. The definition was originally proposed by Berry et al. in Coronary Artery Risk Development in Young Adults (CARDIA) study (*Circulation. 2009 Jan 27;119(3):382-9*), and was subsequently used in other study (*JACC Cardiovasc Imaging. 2012 Feb;5(2):144-53*). Another method to define CAC progression was "SQRT method", which was used in Progression of Early Subclinical Atherosclerosis (PESA) study: difference between square root of baseline and square root of follow-up CAC score >2.5 (*J Am Coll Cardiol. 2020 Apr 14;75(14):1617-1627*). We found that only 8.1% of the participants showed inconsistencies between the two definitions, indicating that the measurement of CAC progression was generally robust. Furthermore, the predictive performance of protein risk scores for CAC progression defined by the "SQRT method" was consistent with our results (not presented in the manuscript).

| Definition of CARDIA | Definition of PESA | |
| --- | --- | --- |
|  | Progressor | Non-progressor |
| Progressor | 236 | 36 |
| Non-progressor | 30 | 513 |

**Revision:** References of the definition of CAC progression in CARDIA study were cited in the Method section. And we added descriptions to help readers better understand the source of definition (Page 7, line 133-140).

5. *Page 8, L162. How were these categories of risk derived? Are these based on the estimated probability of the outcome, or are the 20/60/20 based on the percentiles of the distribution of the probability?*

**Response:** We would like to clarify that the derivation of the ProtRS categories (low, intermediate, and high) were based on the percentiles (< 20%, 20%~80%, ≥ 80%) of the distribution of the ProtRS within the study population other than the probability of the outcome (*Circulation. 2026 Mar 17;153(11):e953-e970*).

**Revision:** We have clarified that the ProtRS risk categories were derived based on the percentiles of the distribution in the Method section.

6. *Page 9, L165. To clarify, did the multivariable model include both the risk category and underlying score as variables?*

**Response:** The association analyses was performed separately for risk category and risk score, instead of including both of them simultaneously.

**Revision:** We have described the details of the multivariable model in the Method section.

7. *P9, L183. The NRI does not need modification with case-cohort data so long as there is appropriate exclusions made to the data (from: https://bmcmedresmethodol.biomedcentral.com/articles/10.1186/1471-2288-13-113). Was the NRI calculated with these exclusions?*

**Response:** We really appreciate the method you provided. The previous NRI value was calculated without using the appropriate exclusions. As suggested, we recalculated NRI by excluding 49 cases that developed ASCVD after 10 years, and considering 7 participants developed ASCVD after 10 years in sub-cohort as non-cases. Results provided less biased evidence under case-cohort design and therefore supported the robustness of the protein risk scores in risk reclassification.

**Revision:** We recalculated NRI that followed appropriate exclusions and described the exclusion criteria in the Method section.

8. *P11, L233. What was the test completed in 'Ptrend'? Was this the overall effect of including the risk score, or was this a directional hypothesis (i.e. low<medium<high)?*

**Response:** The hypothesis was that the risk of CAC and plaque progression were increased with the higher risk categories in protein risk categories. The *P_trend_* reported was derived from regression analysis where the medians of each protein risk categories (Low, Intermediate, and High) were included as continuous numeric variables.

**Revision:** We have clarified the method for the calculation of ‘*Ptrend*’ in the Method section.

9. *P12. L237. Was there evidence (p-value) that the AUC was changed by including ProtRSs? P12. L241. Was there improved AUC compared to a model with just CVD risk factors alone?*

**Response:** Our previous results did not include the evidence that the AUC was improved by including ProtRSs compared to just CVD risk factors alone. According to your suggestion, we presented AUCs of risk factors alone, ProtRSs alone and risk factors combing with ProtRSs for CAC and plaque progression. The risk factors referred to the variables in China-PAR risk model: baseline age, sex, urbanization, smoking status, waist circumference, systolic blood pressure, antihypertensive therapy, total cholesterol, high-density lipoprotein cholesterol, and diabetes. We calculated the Delong’s *P*-value for comparison between reference model (risk factors alone) and with addition of ProtRSs. We observed that there was significant improvement of AUC after adding ProtRSs, with ΔAUC of 2.2% by adding CAC ProtPS with *P*-value of 0.0141 and ΔAUC of 3.6% by adding Plaque ProtRS with *P*-value of 0.0003.

**Revision:** We have revised the Figure of ROC curves, which was presented in Supplemental Figure 5. We also described the risk factors included in reference model and the calculation of Delong’s *P*-value in the Method section (Page 8, line 188-191).

**Supplemental Figure 5. Receiver-operating characteristics curve for the predictive performance of the ProtRS combined with traditional risk factors for SA progression.**

Figure legends: (A) Prediction of CAC progression. (B) Prediction of plaques progression. The risk factors consist of baseline age, sex, urbanization, smoking status, waist circumference, systolic blood pressure, antihypertensive therapy, total cholesterol, high-density lipoprotein cholesterol, and diabetes. The area under the curve (AUC) with its 95% confidence interval is presented in parentheses and incremental AUC improvements of risk factors with addition of protein risk scores to the risk factor model were assessed using Delong’s test. Abbreviations: CAC, coronary artery calcium; ProtRS, protein risk score; SA, subclinical atherosclerosis.

10. *P13. L262. Given there was overlap in the 95% CI of the AUC between the baseline and the model with the risk score, can you explain why the 95% CI for the NRI doesn't cross 0?*

**Response:** AUC and NRI are two different perspectives for evaluating the improvement of model prediction performance with addition of protein risk scores. The AUC quantifies the model's overall discriminative ability (the capacity to separate cases from non-cases), while the NRI assesses whether the addition of protein risk scores to the risk prediction model leads to improvement in reclassification. In our study, we presented significant ΔAUC (Delong’s *P*-value < 0.05) and significant NRI (*P*-value < 0.05), which showed consistency in discrimination and reclassification. As detailed by DeLong et al. (*Biometrics*. 1988;44(3):837-845), the DeLong's test assesses the difference between two AUCs by accounting for the covariance between the errors of the models tested on the same dataset, which is appropriate to determine significance. Other studies also showed overlap in 95% CI of AUC but still presented significant DeLong's *P*-value and significant NRI (*Eur J Epidemiol. 2024 Nov;39(11):1229-1240;* *Eur J Prev Cardiol. 2024 Oct 10;31(14):1681-1689*).

**Revision:** To ensure methodologically interpretation of these different evaluation metrics, we have supplemented the *P*-values (Supplemental Figure 5 displayed the DeLong's *P*-value for the AUC comparison and Supplemental Table 10 displayed the *P*-value for the NRI) and described the details for the calculation of *P-*values in the Method section.

**Reviewer #2:**

*This study focuses on identifying individuals with progressive subclinical atherosclerosis (SA) who are most likely to benefit from early intervention, effectively addressing a critical and unmet clinical need in preventive cardiology. The authors present a well-conducted investigation developing protein risk scores (ProtRSs) to predict the progression of coronary artery calcium (CAC) and carotid plaque, along with a rigorous assessment of their incremental value for improving atherosclerotic cardiovascular disease (ASCVD) risk prediction. Several aspects require further clarification to strengthen its impact.*

**Response:** We deeply appreciate the reviewer for constructive comments. We have addressed all concerns in a point-by-point manner, and we believe that the manuscript has been substantially improved after revision.

1. *The study is based on a pre-defined panel of 116 proteins. To further contextualize the novelty and rationale of the protein panel, the authors should address the following points: a) What was the specific rationale behind the selection of these 116 proteins? b) Were well-established ASCVD protein biomarkers (e.g., NT-proBNP, Troponin) included in this initial pre-defined panel? If so, please clarify whether these established biomarkers were selected by the LASSO model, and discuss how the performance of the newly identified proteins compares to these known markers. This information will* *help readers better understand the incremental value of the study's discoveries within the context of existing CVD biomarker research.*

**Response:** Our study aimed to construct and evaluate the protein risk scores for CAC and plaque progression, as well as assess their utility in ASCVD prediction. Given the pathogenic roles of lipoprotein oxidation inflammation, immune activation, and other key processes in human atherosclerosis, we prioritized Olink CVD-III panel, which covered proteins involved in these pathways of atherogenesis. To further strengthen the biological coverage of inflammation and lipid metabolism, we supplemented this panel with selected interleukins from the Ella Inflammatory Panel and key apolipoproteins measured by biochemical assays, ultimately establishing a targeted panel of 116 proteins. Although this 116-protein panel does not encompass every candidate protein that potentially associated with atherosclerosis, it represents a focused, biologically informed set of candidate biomarkers.

For the established cardiovascular protein markers, NT-proBNP was included in our pre-defined panel, while troponin was not included previously. As you pointed out, troponin is a well-established biomarker to assess myocardial injury in clinical practice (*Circulation. 2021 Dec 14;144(24):1955-1972*). Following your suggestions, we newly detected hsTnI levels used enzymatic immunoassay method and performed protein selection via the LASSO model among 117 proteins (comprising the original 116 proteins plus hsTnI). Our results demonstrated that hsTnI retained non-zero coefficients in 90.6% of iterations of the LASSO model for CAC progression, and was thus included in the protein risk scores (Fig 1). In contrast, NT-proBNP was not included in protein risk scores, as it retained non-zero coefficients in only 2.3% and 22.9% of iterations of the LASSO models for CAC and plaque progression, respectively (Supplemental Table 4).

As you suggested, we compared the predictive performance of well-established cardiovascular biomarkers (NT-proBNP, LPA and PCSK9) and our LASSO-derived atherosclerosis-specific ProtRSs. As expected, we observed that there was significant improvement of AUC after adding ProtRSs to reference model comprising risk factors, withΔAUC of 2.2% for CAC ProtPS (*P =* 0.0141) andΔAUC of 3.6% for plaque ProtRS (*P*=0.0003). In comparison, NT-proBNP, LPA and PCSK9 were not significantly associated with SA progression (all *P*>0.05), and these biomarkers could not provide incremental value for subclinical atherosclerosis progression (Delong’*P*>0.05). These findings explicitly highlight the indispensable value of atherosclerosis-tailored ProtRSs for tracking early vascular disease progression, whereas conventional cardiovascular biomarkers are inherently insufficient for subclinical atherosclerosis risk stratification. For ASCVD prediction, ProtRSs significantly increased the C‑index from 0.724 (95%CI: 0.699‑0.747) to 0.742 (95%CI: 0.717‑0.766) beyond the guideline-recommended China‑PAR score, with an increment of 0.018 (95%CI: 0.003‑0.035). Although hsTnI significantly improved the C-index (ΔC-index: 0.009, 95%CI: 0.003‑0.015) over the China‑PAR score, NT-proBNP did not contribute additional predictive information. Thus, we constructed an updated ProtRSs incorporating hsTnI to enhance the predictive performance in incident ASCVD. Collectively, our ProtRSs improve risk stratification for both SA progression and ASCVD beyond conventional cardiovascular biomarkers, which are limited to overt event prediction and unable to reflect early vascular pathological changes.

**Revision:** To help readers better understand the specific rationale behind the selection of these 117 proteins, we provided detailed descriptions of these proteins in the Method section. We also discussed the performance of our LASSO-derived atherosclerosis-specific ProtRS compares to these established cardiovascular biomarkers (Page 15, line 389-392).

2. *The development of the ProtRSs for CAC and plaque progression is a central contribution of this work. The scores demonstrate robust performance and have clear potential to refine clinical risk stratification. To facilitate the clinical translation and maximize the impact of these promising scores, please provide the recommended cut-off values used to define the risk strata in the analyses and the calculation formula for the ProtRSs.*

**Response:** In the revised manuscript, the ProtRSs for CAC and plaque progression were constructed by 14 proteins using normalized abundance data. And the ProtRSs were calculated on the logit scale as the sum of each protein’s regression coefficient multiplied by its standardized plasma level. The cut-off values were recommended based on the percentiles (<20%, 20%~80%, ≥80%) of the distribution of the ProtRSs, which was also used by other studies (*Eur Heart J. 2022 May 7;43(18):1702-1711; Eur Heart J. 2023 Jan 14;44(3):221-231;* *Circulation. 2026 Mar 17;153(11):e953-e970*). The ProtRSs could use the absolute cut-off values: CAC ProtRS categories (Low: <0.185, Intermediate: 0.185~0.476, and High: ≥0.476), and plaque ProtRS categories (Low: <0.100, Intermediate: 0.100~0.321, and High: ≥0.321).

**Revision:** As suggested, the specific formulas and corresponding cut-off values are provided in Supplemental Table 7.

**Supplemental Table 7. Formulas for the ProtRSs.**

| Proteins | Coefficients in CAC ProtRS | Coefficients in plaque ProtRS |
| --- | --- | --- |
| Intercept | -0.776 | -1.441 |
| Gal-4 | 0.234 | NA |
| IGFBP-7 | 0.191 | NA |
| IL-1B | NA | 0.162 |
| IL-2RA | 0.097 | 0.243 |
| IL-8 | NA | 0.269 |
| TIM-1 | 0.108 | NA |
| TNF-R2 | 0.173 | NA |
| ANGPTL4 | 0.140 | NA |
| CCL24 | NA | -0.171 |
| CPB1 | NA | -0.253 |
| CRP | 0.178 | NA |
| CTSD | 0.144 | 0.088 |
| FASLG | -0.100 | NA |
| hsTnI | 0.119 | NA |
| Insulin | NA | 0.167 |
| KLK6 | -0.303 | NA |
| LEPT | -0.285 | NA |
| MEPE | NA | -0.226 |
| OPG | NA | 0.285 |
| PON3 | -0.189 | -0.208 |
| sdLDL | 0.243 | 0.178 |
| TFPI | NA | 0.283 |
| TNFSF13B | NA | 0.098 |
| TR | NA | -0.285 |

Notes: The ProtRSs were calculated on the logit scale as the sum of each protein’s coefficient multiplied by its standardized plasma level (mean=0, standard deviation=1). The ProtRSs could use the cut-off values to define protein risk categories in cohorts: CAC ProtRS categories (Low: <0.185, Intermediate: 0.185~0.476, and High: >=0.476), and plaque ProtRS categories (Low: <0.100, Intermediate: 0.100~0.321, and High: >=0.321). Abbreviations: ProtRS, protein risk score; SA, subclinical atherosclerosis; CAC, coronary artery calcium; NA, not applicable.

3. *The separation of the discovery set (Beijing Community-Based Cohort of Atherosclerosis Study) for the development of ProtRSs, and the validation set (the International Collaborative Study of Cardiovascular Disease in Asia and the China Multi-Center Collaborative Study of Cardiovascular Epidemiology cohorts) for performance evaluation, provides a good framework that strengthens the validity of the findings. To allow for a complete assessment of validation robustness, please specify the method used to build the prediction model and the procedure for estimating the C-index (e.g., external validation in validation set vs. cross validation in validation set vs. internal validation in validation set).*

**Response:** Thank you for pointing this out. First, the prediction models for CAC and plaque progression were constructed using logistic regression based on the discovery set (the Beijing Community-Based Cohort of Atherosclerosis Study). Second, the protein risk scores were calculated by the derived beta-coefficients and the plasma protein abundance from this model. Finally, to assess model generalizability, the model formula and its coefficients were directly applied to the external validation set. The model's performance was then evaluated by calculating the C-index for ASCVD within the external validation set (the International Collaborative Study of Cardiovascular Disease in Asia and the China Multi-Center Collaborative Study of Cardiovascular Epidemiology cohorts). We provided the flowchart of the study in Supplemental Figure 1 and clarified the method used to build the prediction model and the procedure for estimating the C-index in the Method section (Page 6, line 103-106).

4. *The overall cohort findings are robust. I would recommend supplementary analyses to test the consistency of the association between the ProtRSs and ASCVD events across key clinical subgroups.*

**Response:** Following your recommendation, we performed subgroup analyses across key clinical subgroups and found that the associations between ProtRSs and ASCVD events were consistent across strata of age category, sex, hypertension, dyslipidemia, and diabetes. There were no significant interactions between key clinical subgroups and ProtRSs (*P*-value > 0.05).

**Revision:** We presented the results of subgroup analyses in Supplemental Table 8 and described it in the Result section (Page 12, line 315-317).

**Supplemental Table 8. Subgroup analyses of the associations between ProtRSs and incident ASCVD.**

| Subgroups | CAC ProtRS | | Plaque ProtRS | |
| --- | --- | --- | --- | --- |
|  | HR (95% CI) | *P*-value | HR (95% CI) | *P*-value |
| Age category |  |  |  |  |
| <60 | 1.38 (1.17 - 1.63) | 0.8121 | 1.39 (1.20 - 1.60) | 0.7448 |
| >=60 | 1.42 (1.19 - 1.70) |  | 1.44 (1.21 - 1.70) |  |
| Sex |  |  |  |  |
| Female | 1.34 (1.12 - 1.59) | 0.9856 | 1.42 (1.21 - 1.67) | 0.5138 |
| Male | 1.34 (1.14 - 1.58) |  | 1.32 (1.12 - 1.54) |  |
| Hypertension |  |  |  |  |
| Yes | 1.40 (1.18 - 1.66) | 0.8324 | 1.46 (1.24 - 1.71) | 0.4176 |
| No | 1.36 (1.12 - 1.65) |  | 1.33 (1.13 - 1.56) |  |
| Dyslipidemia |  |  |  |  |
| Yes | 1.39 (1.13 - 1.70) | 0.7192 | 1.42 (1.17 - 1.73) | 0.6271 |
| No | 1.32 (1.12 - 1.55) |  | 1.34 (1.17 - 1.54) |  |
| Diabetes |  |  |  |  |
| Yes | 1.97 (1.28 - 3.03) | 0.0596 | 1.72 (1.18 - 2.49) | 0.2132 |
| No | 1.28 (1.12 - 1.46) |  | 1.34 (1.19 - 1.51) |  |

Notes: Data were presented as HR (95% CI) for incident ASCVD with each SD increase in ProtRSs. Models were adjusted (when appropriate) for age, sex, cohort origin, geographic region, urbanization, smoking status, waist circumference, systolic blood pressure, antihypertensive therapy, total cholesterol, high-density lipoprotein cholesterol, and diabetes. Abbreviations: ASCVD, atherosclerotic cardiovascular disease; SD, standard deviation; CI, confidence interval; HR, hazard ratio; CAC, coronary artery calcium; ProtRS, protein risk score.

*Minor concern:*

5. *Please add a description of "Multivariable - adjusted Log - binomial regressions were used to estimate relative risks (RRs) and 95% confidence intervals (CIs)" in the Methods section, specify the exact covariates included in the multivariable adjustment, to improve the clarity of the model details for readers.*

**Response:** The models were adjusted for variables referred to China-PAR risk model: baseline age, sex, urbanization, smoking status, waist circumference, systolic blood pressure, antihypertensive therapy, total cholesterol, high-density lipoprotein cholesterol, and diabetes.

**Revision:** We have added a detailed description of the covariates adjusted in the multivariable models in the Method section.

6. *To clarify the composition of the models in Supplemental Table 7, please define what "protein risk score" refers to in the rows "Age + sex + protein risk scores" and "China-PAR + protein risk scores"? (i.e., the CAC ProtRS, Plaque ProtRS, SA ProtRSs, or a combined score).*

**Response:** We had clarified the model composition in the revised manuscript. The term "protein risk scores" used in the rows "Age + sex + protein risk scores" and "China-PAR + protein risk scores" refers specifically to both of CAC ProtRS and plaque ProtRS included in the model.

**Revision:** We defined protein risk scores both in notes of tables and in the Method section.

7. *To enhance the interpretability of the net reclassification improvement (NRI) findings, please provide the p-values for the reported categorical NRI estimates.*

**Response:** Following your suggestion, we estimated the *P*-value of categorical NRI through 1000 bootstrapping. Of note, we emphasized the participants with intermediate risk and recalculated the NRI by dividing the risk into three categories (low risk, intermediate risk, and high risk) according to Reviewer’s suggestions. We think the interpretability of NRI was improved after revision.

**Revision:** We presented the specific results of NRI in Supplementary Table 10, and added the *P*-values of categorical NRI.

**Reviewer #3:**

*In this work Huang et al. analyze 116 plasma proteins in a population of 903 individuals and optimize protein scores to predict presence of atherosclerosis progression (plaque and CACs). Then the authors apply these scores to another population of 1835 individuals and show that the scores are able to predict ASCVD events. The authors claim that these scores improve traditional risk scores.*

*The possibility that biomarkers for AT progression can be used to improve prediction of CVD events is really interesting. However, there are several concerns that must be addressed.*

**Response:** We deeply appreciate the reviewer for constructive comments. We have addressed all concerns in a point-by-point manner, and we believe that the manuscript has been substantially improved after revision.

Major points:

1. *LASSO regression is a method adequate to optimize performance of a logistic regression function by eliminating variables that do not contribute to predict progression; however, this method is not restrictive enough to the point to assume that all LASSO-selected proteins are "associated" with progression. Hence any reference to any kind of association must be eliminated from the study.*

*In relation to this, what is the statistical criteria used by the authors to state whether the proteins are "significant" or not in Fig.3?*

*Similarly, the fact that LASSO-selected proteins for CAC or plaque progression are not the same does not imply that the biological framework is different in the two kinds of progression. Hence relations to metabolic traits or inflammation for plaque/CACs should not be traced from the list of LASSO-selected proteins.*

*Hence, although the ProtRS scores constructed with these LASSO-selected proteins can be accepted as valid, I personally am inclined not to trust the biological implications. Hence, the authors should perform an additional analysis directed to determine which proteins are "independently" and significantly associated with progression in order to reach some biological conclusions. This can be done for instance using logistic regression with backward or forward methods in order to obtain minimum panels of proteins concurrently and significantly associated to progression. These data can help the authors to demonstrate that CAC and plaque progression are associated to different proteins.*

**Response:** We appreciate your insightful and valuable comments on our analytical approaches and biological interpretations. As you correctly noted, LASSO regression is a regularization method that compresses the regression coefficients and effectively filters out proteins with minimal contribution to predict disease progression. We fully agree with your point that proteins selected by LASSO regression may not fully overlap with those significantly associated with progression outcomes. Therefore, to complement our LASSO selection and identify proteins independently associated with CAC progression and plaque progression, we additionally performed forward stepwise logistic regression analyses. This complementary strategy allowed us to better infer the biological framework underlying atherosclerotic progression using proteins robustly linked to CAC and plaque progression.

Our results showed that 19 proteins were retained in logistic regression with forward method for plaque progression, which included 13 (92.86%) of the 14 LASSO-selected proteins (except IL-2RA). Similarly, 23 proteins were retained in logistic regression with forward method for CAC progression, including 11 (78.57%) of the 14 LASSO-selected proteins (except TIM-1, hsTnI and FASLG) (Supplemental Table 5). In addition, we found that the effect sizes of proteins estimated by LASSO and logistic regression with forward method showed an extremely high correlation, with Pearson’s correlation coefficients of 0.976 (95% CI: 0.918, 0.993), and 0.899 (95% CI: 0.650, 0.974) for plaque and CAC progression, respectively (*P*-value < 0.001). Collectively, these findings confirmed that the majority of LASSO-identified proteins were also significantly associated with both CAC and plaque progression.

Consistent with your suggestion, we traced the metabolic and inflammatory relevance of proteins linked to plaque and CAC progression using the panel derived from forward logistic regression, instead of LASSO-selected proteins. This protein set provided minimum panels of proteins concurrently and significantly associated with plaque and CAC progression (Supplemental Table 5). Accordingly, we performed KEGG pathway enrichment analysis based on the proteins associated with CAC and plaque progression via forward regression. The results revealed distinct biological frameworks for the two progression phenotypes: proteins related to plaque progression were strongly implicated in inflammatory processes, including cytokine signaling regulation and NF-kappaB activation, whereas proteins associated with CAC progression were enriched in lipid and atherosclerosis pathways (Supplemental Table 6).

For the significant associations in Fig 3, we hypothesized that proteins exerting stronger effects on CAC and plaque progression would confer a higher risk of incident ASCVD. We therefore examined the associations of individual proteins with CAC progression, plaque progression, and ASCVD events, with each protein analyzed in a separate model. Proteins marked as “significant” in Fig.3 were identified based on their significant associations with CAC, plaque progression, or ASCVD, rather than being selected via LASSO regression.

**Fig 3. Comparison of effect sizes between SA progression and ASCVD incidence for proteins with independent association.**

Figure legends: (a) CAC progression vs incident ASCVD. (b) Plaque progression vs incident ASCVD. The 14 proteins comprising the CAC ProtRS and the 14 proteins comprising the plaque ProtRS are presented. Effect sizes for SA progression and ASCVD incidence were estimated using age- and sex-adjusted logistic regressions and Prentice Cox regressions adjusted for age, sex and cohort origin, respectively. Points are colored by the significance of association with both SA progression and ASCVD incidence. Pearson's correlation coefficient (r) and line of best fit are shown. Abbreviation: CAC, coronary artery calcium; SA, subclinical atherosclerosis; ASCVD, atherosclerosis cardiovascular diseases.

**Revision:** We conducted logistic regression with forward method to identify the proteins that associated with CAC and plaque progression, and the results were presented in Supplemental Table 5. We revised all the statements in our manuscript that any reference to any kind of association were eliminated from LASSO-selected proteins, and the associations of proteins were only interpreted in the results of logistic regression. In addition, the biological framework was analyzed and discussed using proteins that were retained in logistic regression with forward method (Supplemental Table 6).

**Supplemental Table 5. Associations between proteins and subclinical atherosclerosis progression.**

| Protein | Inclusion in CAC ProtRS | Inclusion in plaque ProtRS | CAC progression | | Plaque progression | |
| --- | --- | --- | --- | --- | --- | --- |
|  |  |  | OR (95% CI) | *P*-value | OR (95% CI) | *P*-value |
| ALCAM | 0 | 0 | 0.71 (0.53-0.95) | 0.0199 | NA | NA |
| ANGPTL4 | 1 | 0 | 1.22 (1.02-1.45) | 0.0271 | NA | NA |
| AXL | 0 | 0 | 0.83 (0.65-1.06) | 0.1415 | NA | NA |
| CCL24 | 0 | 1 | NA | NA | 0.80 (0.65-0.98) | 0.0308 |
| CNTN1 | 0 | 0 | 1.27 (1.01-1.58) | 0.0376 | NA | NA |
| CPB1 | 0 | 1 | NA | NA | 0.75 (0.61-0.92) | 0.0063 |
| CRP | 1 | 0 | 1.30 (1.04-1.62) | 0.0190 | NA | NA |
| CTSD | 1 | 1 | 1.34 (1.08-1.65) | 0.0066 | 1.25 (0.98-1.60) | 0.0709 |
| Ep-CAM | 0 | 0 | 1.17 (0.97-1.40) | 0.0924 | NA | NA |
| FAS | 0 | 0 | NA | NA | 0.83 (0.65-1.08) | 0.1662 |
| Gal-4 | 1 | 0 | 1.12 (0.92-1.37) | 0.2462 | NA | NA |
| HP | 0 | 0 | NA | NA | 0.80 (0.66-0.97) | 0.0262 |
| ICAM-2 | 0 | 0 | 1.24 (1.02-1.50) | 0.0334 | NA | NA |
| IGFBP-2 | 0 | 0 | NA | NA | 1.45 (1.11-1.88) | 0.0057 |
| IGFBP-7 | 1 | 0 | 1.24 (0.98-1.57) | 0.0669 | NA | NA |
| IL-18BP | 0 | 0 | 0.80 (0.58-1.09) | 0.1494 | NA | NA |
| IL-1B | 0 | 1 | 0.81 (0.62-1.05) | 0.1116 | 1.14 (0.96-1.35) | 0.1314 |
| IL-2RA | 1 | 1 | 1.31 (1.05-1.63) | 0.0148 | NA | NA |
| IL-8 | 0 | 1 | NA | NA | 1.38 (1.02-1.86) | 0.0392 |
| Insulin | 0 | 1 | NA | NA | 1.46 (1.12-1.90) | 0.0047 |
| KLK6 | 1 | 0 | 0.72 (0.58-0.90) | 0.0035 | NA | NA |
| LDL receptor | 0 | 0 | 1.28 (1.04-1.58) | 0.0188 | NA | NA |
| LEPT | 1 | 0 | 0.74 (0.58-0.95) | 0.0182 | NA | NA |
| MCP-1 | 0 | 0 | NA | NA | 0.72 (0.51-1.02) | 0.0651 |
| MEPE | 0 | 1 | NA | NA | 0.76 (0.62-0.93) | 0.0073 |
| MMP-3 | 0 | 0 | 0.77 (0.60-0.98) | 0.0361 | 1.34 (1.02-1.77) | 0.0359 |
| OPG | 0 | 1 | NA | NA | 1.28 (1.01-1.62) | 0.0432 |
| PON3 | 1 | 1 | 0.86 (0.71-1.05) | 0.1419 | 0.74 (0.58-0.93) | 0.0104 |
| RARRES2 | 0 | 0 | 0.79 (0.62-1.00) | 0.0479 | NA | NA |
| sdLDL | 1 | 1 | 1.25 (1.04-1.49) | 0.0166 | 1.24 (1.01-1.54) | 0.0444 |
| TF | 0 | 0 | 0.86 (0.72-1.02) | 0.0825 | NA | NA |
| TFPI | 0 | 1 | NA | NA | 1.33 (1.03-1.72) | 0.0276 |
| TIMP4 | 0 | 0 | NA | NA | 0.77 (0.61-0.97) | 0.0261 |
| TNF-R2 | 1 | 0 | 1.55 (1.15-2.09) | 0.0038 | NA | NA |
| TNFSF13B | 0 | 1 | NA | NA | 1.34 (1.08-1.67) | 0.0076 |
| TR | 0 | 1 | NA | NA | 0.75 (0.60-0.93) | 0.0098 |
| uPA | 0 | 0 | 0.85 (0.70-1.03) | 0.0947 | NA | NA |

Notes: Data were presented as OR (95% CI) for progression with each SD increase in protein level. Forward logistic regression with Akaike information criterion was adopted for model construction, with adjustment for age and sex. A total of 23 and 19 proteins were retained in the final CAC and plaque progression models, respectively. Abbreviations: SD, standard deviation; CI, confidence interval; OR, odds ratio; CAC, coronary artery calcium; ProtRS, protein risk score; NA, not applicable.

2. *Some of the proteins incorrectly claimed to be associated to progression/incidence have been previously described as associated to atherosclerosis. Thus, if association is finally confirmed, these proteins must be discussed at the light of current bibliography.*

**Response:** In response to your concern, we additionally conducted forward stepwise logistic regression to validate the associations between LASSO-selected proteins and progression of CAC and atherosclerotic plaques. As demonstrated above, the majority of LASSO-derived proteins were concurrently incorporated into the forward regression models and also remained significantly associated with both plaque and CAC progression (Supplemental Table 5). Notably, while not all proteins linked to showed significant associations with atherosclerosis progression, the protein effect sizes derived from LASSO and forward stepwise logistic regression exhibited extremely strong correlations, with Pearson correlation coefficients of 0.976 (95% CI: 0.918–0.993) for plaque progression and 0.899 (95% CI: 0.650–0.974) for CAC progression (all *P* < 0.001). Based on these validated proteins robustly linked to CAC and plaque progression, we conducted KEGG pathway enrichment analyses to explore the underlying biological framework. Our results revealed that proteins associated with plaque progression were mainly involved in inflammatory processes, including the regulation of cytokine signaling and mediation of NF‑kappaB activation, whereas proteins related to CAC progression were enriched in lipid and atherosclerosis pathways (Supplemental Table 6). This refined biological interpretation strengthens the rationality and mechanistic relevance of our findings for both progression phenotypes.

Accordingly, we have thoroughly revised the Discussion section by focusing only on proteins statistically validated to be associated with plaque and CAC progression. We now highlight key proteins that represent distinct and biologically relevant biomarkers for CAC and plaque progression, as well as novel molecules implicated in atherosclerosis. Furthermore, we have rigorously discussed the potential mechanisms by which these proteins may contribute to atherosclerosis and ASCVD pathogenesis, with detailed reference and integration of previous published literature (Page 14, line 360-379).

3. *Four proteins are preselected and included in the protein panels. In spite that these proteins are known to be related with AT, the reason for forcing them is unclear and needs an adequate explanation. Would these proteins have been selected by LASSO if included originally? Are these proteins directly associated to progression/ASCVD incidence after adjusting by risk factors?*

**Response:** We appreciate your constructive comments and careful review of our prespecified protein selection. First, we would like to clarify that the four proteins — NT-proBNP, LPA, PCSK9, and GDF‑15 — were not selected by the original LASSO model. We initially considered including these proteins in our protein risk scores based on strong existing evidence supporting their causal roles in cardiovascular diseases, as documented in previous literature (*Nat Rev Endocrinol. 2021 Oct;17(10):592‑607; Lancet Diabetes Endocrinol. 2016 Oct;4(10):840‑9; Nat Rev Cardiol. 2024 May;21(5):299‑311*).

In our dataset, PCSK9 and GDF‑15 were significantly associated with incident ASCVD (HR: 1.35, 95%CI: 1.18‑1.53 for GDF‑15; HR: 1.14, 95%CI: 1.01‑1.27 for PCSK9), whereas LPA and NT‑proBNP showed marginal associations (HR: 1.11, 95%CI: 0.98‑1.26 for NT‑proBNP; HR: 1.05, 95%CI: 0.94‑1.16 for LPA). Regarding progression phenotypes, only GDF‑15 was significantly associated with both CAC progression (OR: 1.31, 95%CI: 1.10‑1.56) and plaque progression (OR: 1.25, 95%CI: 1.02‑1.52). In contrast, LPA, NT‑proBNP, and PCSK9 showed no significant associations with either CAC progression (OR: 1.07, 0.92‑1.23; OR: 1.09, 0.93‑1.28; OR: 1.12, 0.96‑1.30, respectively) or plaque progression (OR: 1.05, 0.89‑1.25; OR: 1.13, 0.94‑1.35; OR: 1.11, 0.93‑1.32, respectively).

According to another reviewer’s suggestion, we newly detected hsTnI, a well-established biomarker to assess myocardial injury, in our two cohorts. Notably, hsTnI maintained non-zero coefficients in 90.6% of iterations of the LASSO model for CAC progression. Moreover, addition of hsTnI significantly improved the discriminative performance of the guideline-endorsed China-PAR score for incident ASCVD, with a ΔC-index of 0.009 (95% CI: 0.003–0.015). Accordingly, in the revised manuscript, we have constructed an updated ProtRS by integrating hsTnI. To ensure consistency in model construction, we reconstructed ProtRSs only using LASSO‑selected proteins in the revised manuscript, with the four previously preselected proteins removed entirely.

4. *Mendelian randomization analysis to detect causal relations are difficult to interpret when non-genetic data is employed, without a strong justification of assumptions, confounders and so on. Why the authors do not perform a direct association analysis of the proteins with the variables, without trying to detect causality?*

**Response:** According to your suggestion, we performed logistic regressions with forward method to detect the direct associations of proteins with CAC and plaque progressions. Although the Mendelian randomization did not yield direct evidence for these association, it provided supportive evidence for our findings. Therefore, we retained Mendelian randomization in the Discussion section as supplementary evidence on the casual effects of proteins on atherosclerosis (Supplemental Table 11) and removed the contents of Mendelian randomization from the Methods and Results section.

**Revision:** We added Supplemental Table 5 to present direct associations of proteins with CAC and plaque progressions and removed the contents of Mendelian randomization from the Methods and Results section.

5. *the authors state that the ProtRS scores including CV risk factors have predictive performance, but do not compare this performance with that obtained using CV risk factors without the scores. In addition, the increase in performance upon the addition of Prot scores to conventional risk factors or to conventional risk scores must be statistically validated in terms of significance (p-values). It would be highly advisable to perform DeLong's test or similar to determine whether the increase in AUC value after adding the protein score is statistically significant.*

**Response:** According to your suggestion, we presented AUCs of risk factors alone, ProtRSs alone and risk factors combing with ProtRSs for CAC and plaque progression. The risk factors referred to the variables used in China-PAR risk model: baseline age, sex, urbanization, smoking status, waist circumference, systolic blood pressure, antihypertensive therapy, total cholesterol, high-density lipoprotein cholesterol, and diabetes (*Circulation. 2016 Nov 8;134(19):1430-1440*). We calculated the Delong’s *P*-value for comparison between reference model (risk factors alone) and with addition of ProtRSs. We observed that there was significant improvement of AUC for CAC and plaque progression after adding ProtRSs, with ΔAUC of 2.2% by adding CAC ProtPS with *P*-value of 0.0141 and ΔAUC of 3.6% by adding Plaque ProtRS with *P*-value of 0.0003.

**Revision:** We have revised the Figure of ROC curves for CAC and plaque progression, which was presented in Supplemental Figure 5. We also described the risk factors included in reference model and the calculation of Delong’s *P*-value in the Method section (Page 8, line 188-191).

**Supplemental Figure 5. Receiver-operating characteristics curve for the predictive performance of the ProtRS combined with traditional risk factors for SA progression.**

Figure legends: (A) Prediction of CAC progression. (B) Prediction of plaques progression. The risk factors consist of baseline age, sex, urbanization, smoking status, waist circumference, systolic blood pressure, antihypertensive therapy, total cholesterol, high-density lipoprotein cholesterol, and diabetes. The area under the curve (AUC) with its 95% confidence interval is presented in parentheses and incremental AUC improvements of risk factors with addition of protein risk scores to the risk factor model were assessed using Delong’s test. Abbreviations: CAC, coronary artery calcium; ProtRS, protein risk score; SA, subclinical atherosclerosis.

6. *The key achievement of this study is summarized in Table 2 and in Figure 3. In resumed terms, these data show that the ProteinScore is associated to progression in the discovery cohort and to ASCVD incidence in the validation cohort with a similar effect size for the overall ProteinScore (Table) or for each one of the proteins included in the score (Figure). This extraordinarily good coincidence between the absolute values of the coefficients is rather surprising, given not only that they are assessing different parameters (RR in one case and HR in the other; two parameters that may be quite different, even in the same cohort and outcome, depending on the kaplan-meyer type of behaviour along time, since RR measures the total fraction in the total time lapse, while HR measures the instantaneous effect on the hazard rate), but given also that the actual variables (progression and incidence of events) and also the cohorts are different.* *There are difficulties to accept such a good correspondence without a proper discussion that help to explain it or a comparison with other works achieving similar results (in this or other diseases).*

*Besides, such a good correspondence implies that progression is an extraordinary good predictor of the risk of having ASCVD events. While the two outcomes are obviously related, it is also well known that not all progressors develop events and that non-progressors may develop events. Again, this finding must be discussed at the light of other studies obtaining similar results.*

**Response:** The core rationale of our study is that identifying individuals with progressive subclinical atherosclerosis can provide critical value for early ASCVD prevention (*JAMA. 2012;308(8):788–795; JAMA. 2026 Jan 20;335(3):245-254; JACC Cardiovasc Imaging. 2015;8(11):1285–1293*). This approach is further supported by the recent Lancet Commission on Rethinking Coronary Artery Disease (*Lancet. 2025 Apr 12;405(10486):1264-1312*), which emphasizes an urgent shift from late event‑centered care toward early detection and prevention of atherosclerotic plaque progression. Traditional risk models predominantly focus on hard ASCVD events, whereas our study addresses an important gap by identifying protein biomarkers associated with subclinical atherosclerosis progression.

Against this background, we hypothesized that proteins showing stronger effects on CAC and plaque progression would also confer higher risks of incident ASCVD. We therefore evaluated the correlation between effect sizes of individual proteins for subclinical progression and those for ASCVD events. A high correlation would support the notion that these proteins may biologically link subclinical atherosclerosis progression to subsequent clinical events, a concept supported by previous study (*Nat Commun. 2015 Dec 22:6:10206*). Similar analytical strategies have been widely applied across diverse diseases and traits, including asthma, lipid metabolism, and brain aging, using different populations and statistical frameworks (*J Allergy Clin Immunol. 2014;133(6):1564–1571; Nat Genet. 2017;49(12):1722–1730; Nat Aging. 2025;5(10):2086–2103*).

As you appropriately noted, directly comparing ORs and HRs may introduce interpretive challenges. In the revised manuscript, we have therefore recalculated Pearson correlation coefficients using the coefficients (β) for each protein instead of ORs and RRs, which fitted in the normal distribution assumption in Pearson correlation analysis. The results confirmed statistically significant correlations between effect magnitudes for subclinical progression and ASCVD: r = 0.77 (*P* = 0.0012) for CAC progression, and r = 0.85 (*P* = 0.0001) for plaque progression. These findings support the biological coherence between protein‑related effects on early atherosclerotic progression and subsequent clinical cardiovascular events.

We acknowledge your point that not all progressors develop events and vice versa, and that the strong correspondence warrants careful contextualization. Furthermore, although not all proteins associated with SA progression in the logistic regression analysis were significantly associated with ASCVD, the effect estimates of the majority of proteins (92.9%, 13/14) exhibited directional consistency. Accordingly, we have substantially revised the Discussion to interpret the high correlation in the context of the causal continuum linking subclinical plaque progression to ASCVD.

**Revision:** We updated Figure 3 to display the recalculated Pearson correlations based on effect sizes for associations with CAC/plaque progression and ASCVD. We also expanded the Discussion to compare our results with previous literature demonstrating analogous patterns across intermediate biomarkers and clinical outcomes, thereby providing a more thorough interpretation of this key finding (Page 15, line 392-398).

**Figure 3. Comparison of effect sizes between SA progression and ASCVD incidence for proteins with independent association.**

Figure legends: (a) CAC progression vs incident ASCVD. (b) Plaque progression vs incident ASCVD. The 14 proteins comprising the CAC ProtRS and the 14 proteins comprising the plaque ProtRS are presented. Effect sizes for SA progression and ASCVD incidence were estimated using age- and sex-adjusted logistic regressions and Prentice Cox regressions adjusted for age, sex and cohort origin, respectively. Points are colored by the significance of association with both SA progression and ASCVD incidence. Pearson's correlation coefficient (r) and line of best fit are shown. Abbreviation: CAC, coronary artery calcium; SA, subclinical atherosclerosis; ASCVD, atherosclerosis cardiovascular diseases.

*Other points that must be addressed:*

7. *three cohorts were used, BCBCAS, InterASIA and ChinaMUCA. The publications describing each of them must be included, and the references of these cohorts also indicated*

**Response:** Thank you for pointing this out. We have now added the original references for all three cohorts (BCBCAS, InterASIA, and ChinaMUCA) and have indicated them in the main text where each cohort is first introduced.

8. *the validation (ASCVD) cohort was constructed using two separate cohorts, but the details are lacking. How many from each in Table 1? How many cases and controls.. etc*

**Response:** For the InterASIA cohort, the final sample included 758 sub-cohort members and 272 additional incident ASCVD cases identified outside the sub-cohort. For the ChinaMUCA cohort, the final sample included 633 sub-cohort members and 172 additional incident ASCVD cases outside the sub-cohort.

**Revision:** As suggested, we provided the detailed sample size for each cohort in the Method section.

9. *Table 1 must indicate the distribution of parameters in the subpopulation of CAC and of plaque progressors (i.e. the cases) and of* *non progressors (i.e. the controls), to detect potential bias. Moreover, drug/medications should be included and the potential effect on the results observed should be analyzed.*

**Response:** According to your suggestion, we added the drug/medications information in Table 1 and Supplemental Table 3, and provided the distribution of parameters in the subpopulation of CAC and plaque progressors and non-progressors (Supplemental Table 2). To evaluate the potential effect of medications on the predictive models, we additionally considered treated hyperlipidemia and treated diabetes into the risk factors as base model (treated hypertension had been included in the original results) to calculate AUCs for CAC and plaque progression. The results showed that the conclusions on predictive models could not be affected by medications, which were presented in Supplemental Figure 7.

**Supplemental Table 2. Baseline characteristics of Beijing Community-Based Cohort of Atherosclerosis Study**

| Baseline characteristics | Plaque progression | | | CAC progression | | |
| --- | --- | --- | --- | --- | --- | --- |
|  | non-progressor (n=609) | progressor (n=172) | *P*-value | non-progressor (n=543) | progressor (n=272) | *P*-value |
| Age, mean (SD), years | 53.5 (6.2) | 56.6 (6.9) | <0.001 | 53.2 (6.1) | 56.1 (6.5) | <0.001 |
| Female, n (%) | 354 (58.1) | 65 (37.8) | <0.001 | 322 (59.3) | 130 (47.8) | 0.002 |
| Urban region, n (%) | 217 (35.6) | 41 (23.8) | 0.005 | 168 (30.9) | 103 (37.9) | 0.057 |
| High school education and above, n (%) | 211 (34.6) | 41 (23.8) | 0.010 | 181 (33.3) | 85 (31.2) | 0.604 |
| Smoking status, n (%) |  |  | <0.001 |  |  | 0.012 |
| Never | 391 (64.2) | 75 (43.6) |  | 344 (63.4) | 149 (54.8) |  |
| Former | 41 (6.7) | 21 (12.2) |  | 47 (8.7) | 19 (7.0) |  |
| Current | 177 (29.1) | 76 (44.2) |  | 152 (28.0) | 104 (38.2) |  |
| Alcohol drinking, n (%) | 204 (33.5) | 74 (43.0) | 0.027 | 182 (33.5) | 100 (36.8) | 0.400 |
| Systolic blood pressure, mean (SD), mm Hg | 132.3 (17.4) | 141.9 (18.5) | <0.001 | 131.5 (17.4) | 140.4 (19.0) | <0.001 |
| Diastolic blood pressure, mean (SD), mm Hg | 82.8 (10.5) | 85.5 (10.1) | 0.003 | 82.7 (10.5) | 85.1 (10.6) | 0.002 |
| Treated hypertension, n (%) | 122 (20.0) | 72 (41.9) | <0.001 | 94 (17.3) | 101 (37.1) | <0.001 |
| Waist circumference, mean (SD), cm | 88.5 (9.9) | 90.4 (10.8) | 0.033 | 88.0 (9.7) | 91.2 (10.4) | <0.001 |
| LDL-C, mean (SD), mmol/L | 3.0 (0.9) | 3.2 (0.8) | 0.003 | 3.0 (0.8) | 3.1 (0.9) | 0.337 |
| HDL-C, mean (SD), mmol/L | 1.4 (0.3) | 1.3 (0.3) | 0.132 | 1.4 (0.3) | 1.3 (0.3) | 0.001 |
| Total cholesterol, mean (SD), mmol/L | 5.1 (0.9) | 5.4 (1.0) | 0.010 | 5.2 (0.9) | 5.3 (1.0) | 0.013 |
| Triglycerides, median [Q1, Q3], mmol/L | 1.4 [1.0, 2.0] | 1.6 [1.0, 2.3] | 0.050 | 1.4 [1.0, 1.9] | 1.6 [1.1, 2.3] | <0.001 |
| Treated hyperlipidemia, n (%) | 18 (3.0) | 9 (5.2) | 0.227 | 9 (1.7) | 16 (5.9) | 0.002 |
| Diabetes mellitus, n (%) | 76 (12.5) | 43 (25.0) | <0.001 | 52 (9.6) | 73 (26.8) | <0.001 |
| Treated diabetes, n (%) | 42 (6.9) | 25 (14.5) | 0.003 | 29 (5.3) | 37 (13.6) | <0.001 |
| Family history of ASCVD, n (%) | 326 (53.5) | 101 (58.7) | 0.262 | 279 (51.4) | 149 (54.8) | 0.400 |

Notes: Values are presented as mean (SD), median [Q1, Q3], or n (%). Comparisons between progressor and non-progressor were made with Student’s t-test (or Wilcoxon test) and chi-square test for continuous and categorical variables, respectively. Abbreviations: ASCVD, atherosclerosis cardiovascular diseases; Q1, first percentile; Q3, third percentile; SD, standard deviation; HDL-C, high-density lipoprotein cholesterol; LDL-C, low-density lipoprotein cholesterol; CAC, coronary artery calcium. SI conversion factor: To convert total cholesterol, LDL-C, and HDL-C to mg/dL, multiply by 38.6.

**Supplemental Figure 7. Receiver-operating characteristics curve for the predictive performance of the ProtRS combined with traditional risk factors for SA progression (included medications).**

Figure legends: (A) Prediction of CAC progression. (B) Prediction of plaques progression. The risk factors consist of baseline age, sex, urbanization, smoking status, waist circumference, systolic blood pressure, antihypertensive therapy, total cholesterol, high-density lipoprotein cholesterol, diabetes, treated hyperlipidemia, and treated diabetes. The area under the curve (AUC) with its 95% confidence interval is presented in parentheses and incremental AUC improvements of risk factors with addition of protein risk scores to the risk factor model were assessed using Delong’s test. Abbreviations: CAC, coronary artery calcium; ProtRS, protein risk score; SA, subclinical atherosclerosis.

10. *Have any of the individuals belonging to the ASCVD control population (i.e. the 10% taken at random) developed incident ASCVD? The authors only state that they do not have ASCVD at baseline (2007-2008), but this does not preclude the possibility that they develop it during the time lapse considered.*

**Response:** We adopted case-cohort design to validate the performance of ProtRSs in predicting ASCVD based on its cost-effective manner and following the original prospective design (*BMC Med Res Methodol. 2013 Sep 13:13:113*). The finally included participants consisted of two parts from the original cohort, including random sample of the original cohort (the sub-cohort) as well as all additional incident cases. The subjects in sub-cohort could develop events during follow-up, and the cases within sub-cohort and outside sub-cohort were assigned different weights when conducting analyses (*J Clin Epidemiol. 1999 Dec;52(12):1165-72*).

Among 758 participants from the sub-cohort of the InterASIA cohort, 44 developed ASCVD, and among 633 participants from the sub-cohort of the ChinaMUCA cohort, 21 developed ASCVD, we have included this information in the revised manuscript.

**Revision:** We added the number of individuals who developed ASCVD in the sub-cohort in the Method section.

11. *What is the criteria used to state "**absence of ASCVD" in the ASCVD cohort? Lack of ASCVD events, as defined for "incident ASCVD"? Please clarify*

**Response:** We stated that “participants of ASCVD cohort were absence of ASCVD at baseline”, indicating that none of the participants had been clinically diagnosed as ASCVD by a physician, nor self-reported incident ASCVD during interviews with participants or their proxies before 2007-2008 baseline assessment. The incident ASCVD was defined as the first occurrence of nonfatal acute myocardial infarction, coronary heart disease death, fatal or nonfatal ischemic stroke.

**Revision:** We have clarified the criteria of "absence of ASCVD" in the Method section.

12. *please indicate whether the definition of progression is in agreement with the criteria used in other studies and cite corresponding references. Is the criterion used to define CAC progression based on a previously published methodology? It this threshold of >10% clinically relevant? Why plaque progression was only defined as an increase in the number of plaques at follow-up and not also as an increase in plaque thickness?*

**Response:** The definition of CAC progression used in our manuscript was commonly used. It was originally proposed by Berry et al. in Coronary Artery Risk Development in Young Adults (CARDIA) study (*Circulation. 2009 Jan 27;119(3):382-9*). And it was subsequently used in other studies (*JACC Cardiovasc Imaging. 2012 Feb;5(2):144-53.; Br J Sports Med. 2022;56(15): 854-61*). The thresholds, including the annualized percent change in CAC≥10% used for high baseline scores (CAC≥100) was initially developed to address the challenges posed by the large number of zeros and skewed distribution for CAC change data. It was observed that CAC progression was significantly associated with a higher risk of CVD events (*Br J Sports Med. 2022;56(15): 854-61*).

The plaque definition does rely on thickness initially, as plaque presence in each carotid artery is first determined by measuring the focal plaque thickness. However, in order to reflect the overall burden of plaque atherosclerosis across the carotid arteries (the common carotid artery, carotid bifurcation, and internal carotid artery), the numbers of these defined plaques across all segments are then aggregated into a plaque score. Therefore, an increase in this total number of plaques at follow-up is used as the primary metric to define its progression instead of an increase in plaque thickness. And this definition has been used in the previous MESA study (*Stroke. 2014 Nov;45(11):3257-62*). In comparison, there were no commonly used criteria for IMT progression, for the measurements of thickness were varied across carotid segments, type of measurements (mean of the mean or mean of the maximum), and number of imaging angles used, and carotid plaque appears to be a more powerful predictor of CV risk compared with cIMT (*JACC Cardiovasc Imaging. 2014 Oct;7(10):1025-38*).

**Revision:** References of the definition of CAC progression in CARDIA study and definition of plaque progression in MESA were cited in the Method section. And we added descriptions to help readers better understand the source of definition.

13. *It is essential that a "minimal data set" is presented to allow these results to be inspected or reproduced by other authors. Hence a table with the protein measurements in all the invididuals of both cohorts (discovery and validation) of at least the proteins used to construct the scores must be presented (indicating the groups to which individuals belong). Also the proteins coefficients for the two scores (CAC and plaque) must be presented in a Supp. Table.*

**Response**: We fully agree that the minimal dataset and formulas are crucial for reproducibility and clinical application. As suggested, the specific formulas and corresponding cut-off values of ProtRSs were provided in Supplemental Table 7. And the "minimal data set" that we used for this study was presented in Supplemental Table 12 and Supplemental Table 13. The datasets included the protein measurements that used to construct ProtRSs in all individuals of both cohorts (with the outcome data).

**Supplemental Table 7. Formulas for the ProtRSs.**

| Proteins | Coefficients in CAC ProtRS | Coefficients in plaque ProtRS |
| --- | --- | --- |
| Intercept | -0.776 | -1.441 |
| Gal-4 | 0.234 | NA |
| IGFBP-7 | 0.191 | NA |
| IL-1B | NA | 0.162 |
| IL-2RA | 0.097 | 0.243 |
| IL-8 | NA | 0.269 |
| TIM-1 | 0.108 | NA |
| TNF-R2 | 0.173 | NA |
| ANGPTL4 | 0.140 | NA |
| CCL24 | NA | -0.171 |
| CPB1 | NA | -0.253 |
| CRP | 0.178 | NA |
| CTSD | 0.144 | 0.088 |
| FASLG | -0.100 | NA |
| hsTnI | 0.119 | NA |
| Insulin | NA | 0.167 |
| KLK6 | -0.303 | NA |
| LEPT | -0.285 | NA |
| MEPE | NA | -0.226 |
| OPG | NA | 0.285 |
| PON3 | -0.189 | -0.208 |
| sdLDL | 0.243 | 0.178 |
| TFPI | NA | 0.283 |
| TNFSF13B | NA | 0.098 |
| TR | NA | -0.285 |

Notes: The ProtRSs were calculated on the logit scale as the sum of each protein’s coefficient multiplied by its standardized plasma level (mean=0, standard deviation=1). The ProtRSs could use the cut-off values to define protein risk categories in cohorts: CAC ProtRS categories (Low: <0.185, Intermediate: 0.185~0.476, and High: >=0.476), and plaque ProtRS categories (Low: <0.100, Intermediate: 0.100~0.321, and High: >=0.321). Abbreviations: ProtRS, protein risk score; SA, subclinical atherosclerosis; CAC, coronary artery calcium; NA, not applicable.

*Minor comments:*

14. *Page 3, Line 50: 'were implicated' instead of 'implicated'.*

**Response**: We have revised the text as suggested in our manuscript.

15. *In Study design and population section the first paragraph refers to S2A Figure and the second to S2B Figure, please correct it in the text. In Figure 2SA it is described that the population for the study of protein biomarkers and construct risk scores comprises 903 participants, but then only 815 individuals are used to study CAC progression and 781 to study plaque progression. These differences in the number of individuals used for each study should be clarified in the text. Are they due to the lack of plaque and CAC measurements?*

**Response**: We have revised the text to explicitly reference the figure panels (S2A Fig and S2B Fig) that detail the study populations used for the CAC and plaque progression analysis and the ASCVD predictive analysis, respectively.

The difference in sample size between the CAC (n=815) and plaque (n=781) progression analyses is due to the lack of corresponding CAC or plaque measurement data. Not every participant completed both CAC and plaque measurements. To ensure clarity on the actual data sets used, we have indicated the number of plaque and CAC progressor and non-progressor in Supplemental Table 2 and in the Method section.

16. *In Data collection section and outcome definition or in* *supplemental text authors should include more details about blood samples processing, type of tubes. The collection of blood samples and their subsequent processing are crucial to ensure that proteomic studies yield reliable results. Did the authors used plasma or serum samples?*

**Response**: The plasma samples were used in this study. Blood samples were collected by professionally trained technical staff with participants fasting for ≥10 hours of overnight. Disposable vacuum blood collection tubes were used for sampling, including tubes containing ethylenediaminetetraacetic acid (EDTA) as an anticoagulant and plain serum tubes. Both plasma samples and serum samples were then processed at the field center. Immediately after collection, the EDTA tubes were inverted 5–6 times to ensure adequate mixing with the anticoagulant. All blood samples were centrifuged within 2 hours of collection (2000g, 4℃, 10 minutes). After plasma separation, the samples were aliquoted into 1.5mL EP tubes and stored in a -80℃ freezer. All biospecimens were transported using a cold chain process and were centrally stored in the biobank at Fuwai Hospital, Chinese Academy of Medical Sciences. Finally, the proteins were detected using plasma samples.

**Revision:** The information on blood sample collection was added in supplemental text and stated in the Method section.

17. *In Page 11, Line 218 the value reported from the KEGG analysis is not a p-value; rather, it corresponds to an FDR value, as indicated in the supplementary table S5.*

**Response:** According to your suggestion, we perform KEGG analysis using proteins that selected by logistic regressions with forward method to explore biological framework. And we presented both *P*-value and *P_FDR_* in Supplemental Table 6.

**Reviewer #4:**

*This study aimed to identify candidate plasma proteins accelerating development of subclinical atherosclerosis (CACS and carotid plaques) over a 5 - year period. These proteins were built into two different protein risk scores (ProtRSs CACS/Plaque). The predictive performance was then evaluated for ASCVD in a separate case-cohort design. The study mainly confirms previous studies with similar changes in C-statistics and NRI. Difficult to assess the value of individual proteins since no formal Mendelian randomization has been performed and there is no FDR correction for multiple testing. A novelty is the application in an Asian cohort.*

*The study appears well performed using an interesting design to identify candidate proteins that affect atherosclerosis and then testing their predictive capacity in a case-cohort. The study is well written using appropriate methodology and statistics. However, the reporting is not well balanced and tends to exaggerate the results by cherry-picking data. I have some comments/questions/suggestions.*

**Response:** We sincerely appreciate your insightful comments. As you noted, previous protein-related studies, which primarily focused on proteins linked to established clinical ASCVD events, have shown moderate predictive value for ASCVD. In contrast, our study shifts the focus to proteins driving subclinical atherosclerosis progression over 6 years. By identifying proteins accelerating CAC and carotid plaque development (key preclinical atherosclerotic stages), we target biological pathways driving the disease process, not just ASCVD markers, addressing a major unmet need in proteomic understanding of atherosclerosis. Another novelty of our study is the application of this approach in an Asian cohort with distinct genetic and lifestyle characteristics.

We also fully acknowledge the points you raised, including the potential for exaggerated results due to cherry-picking, no formal Mendelian randomization (MR), and the lack of FDR correction for multiple testing. Below, we address each of your concerns point-by-point and detail the revisions we have made to the manuscript.

First, the statements in our manuscript may have misled readers for interpretation of our results. To address this, we have thoroughly revised the manuscript in accordance with the criteria for assessing the predictive and clinical utility of biomarkers for cardiovascular disease risk, as outlined in the American Heart Association guidelines (*Circulation. 2026 Mar 17;153(11):e953-e970*). Specifically, in Results section, we have added a detailed NRI table and described the sample size of cases that were misclassified into the low-risk category and the number of controls incorrectly classified into the high-risk category, allowing for a more objective assessment of the clinical utility of our risk scores. And in the Discussion, we have added a dedicated subsection on potential caveats and limitations of ProtRSs in clinical application, to avoid over-interpreting their predictive value and ensure balanced reporting. We explicitly emphasized that the ProtRSs may serve as risk enhancers to further refine clinical risk assessment.

Second, to better explore the biological framework of CAC and plaque progression, we supplemented logistic regression analyses with a forward method to confirm the associations of proteins with CAC and plaque progression and then conducted KEGG analysis based on these proteins. The Mendelian randomization was just retained in the Discussion section to provide supportive evidence for the potential causal roles of the identified proteins.

Finally, for multiple testing, we have now applied FDR correction to *P*-values, and several sensitivity and subgroup analyses were performed to show the robustness and consistency of our results. We have updated relevant tables and results sections to present the adjusted *P*-values, ensuring the rigor of our statistical analyses.

Once again, we thank you for your valuable suggestions. We hope these revisions address your concerns fully and enhance the overall quality of our study.

1. *Please include information in the main text that both cohorts are free of previous ASCVD at baseline.*

*The definition of plaques includes general IMT thickening (not focal). I do not think this matches criteria in the Mannheim consensus. Please comment whether inflammatory conditions affecting IMT can affect your classification of atherosclerosis.*

**Response:** As you suggested, we have revised the main text to explicitly clarify the baseline ASCVD status of both cohorts. Specifically, the ASCVD case-cohort participants were free of ASCVD at enrollment. The atherosclerosis population was not entirely free of ASCVD at baseline, consistent with our focus on atherosclerosis progression across varying baseline disease degrees. We also performed sensitivity analyses after excluding 34 participants with prevalent ASCVD at baseline. These analyses yielded consistent and robust results, with AUCs of 0.710 vs 0.711 for CAC progression and 0.722 vs 0.725 for plaque progression (Supplementary Figure 8), confirming the reliability of our findings.

Regarding carotid plaque definition, we apologize for the ambiguity in the original manuscript. Our current definition of carotid plaques is based on the identification of discrete, focal structures distinct from the surrounding vascular tissue, rather than general intima-media thickness (IMT) thickening—this is fully consistent with the criteria established in the Mannheim Consensus. To eliminate confusion, we have added the specific term "focal" to the plaque definition in the Method section for clarity.

Regarding whether inflammatory conditions, we acknowledge that certain inflammatory conditions (e.g., vasculitis) could potentially interfere with plaque diagnosis. However, no participant in our cohort reported a history of vasculitis at baseline. Additionally, physicians strictly followed standardized protocols for plaque detection and conducted differential diagnosis to distinguish between vasculitis and true atherosclerotic plaques. Thus, we concluded that inflammatory conditions had limited impact on our classification of atherosclerosis.

**Revision:** We clarified the definition of the carotid plaques and added the information of baseline ASCVD in the Method section. The results of sensitivity analysis were presented in Supplemental Figure 8.

**Supplemental Figure 8. Receiver-operating characteristics curve for the predictive performance of the ProtRS combined with traditional risk factors for SA progression (excluded participants with ASCVD at baseline).**

Figure legends: (A) Prediction of CAC progression. (B) Prediction of plaques progression. The risk factors consist of baseline age, sex, urbanization, smoking status, waist circumference, systolic blood pressure, antihypertensive therapy, total cholesterol, high-density lipoprotein cholesterol, and diabetes. The area under the curve (AUC) with its 95% confidence interval is presented in parentheses and incremental AUC improvements of risk factors with addition of protein risk scores to the risk factor model were assessed using Delong’s test. Abbreviations: CAC, coronary artery calcium; ProtRS, protein risk score; SA, subclinical atherosclerosis.

2. *There were four a priori selected proteins included in the ProtRS. I can not find them in Fig 1. Why? Did these proteins associate with ASCVD?*

**Response:** We apologize for any confusion caused by insufficient explanation in the original manuscript, and we have clarified and revised the relevant content accordingly.

First, we would like to clarify that the four prespecified proteins — NT-proBNP, LPA, PCSK9, and GDF‑15 — were not identified or selected by the LASSO regression procedure used to construct the primary ProtRS. Instead, we initially considered incorporating these proteins based on robust prior evidence supporting their causal roles in cardiovascular and atherosclerotic diseases, as documented in previous reviews (*Nat Rev Endocrinol. 2021 Oct;17(10):592‑607; Lancet Diabetes Endocrinol. 2016 Oct;4(10):840‑9; Nat Rev Cardiol. 2024 May;21(5):299‑311*).

In our dataset, PCSK9 and GDF‑15 were significantly associated with incident ASCVD (HR: 1.35, 95%CI: 1.18‑1.53 for GDF‑15; HR: 1.14, 95%CI: 1.01‑1.27 for PCSK9), and NT‑proBNP and LPA showed marginally significant associations (HR: 1.11, 95%CI: 0.98‑1.26 for NT‑proBNP; HR: 1.05, 95%CI: 0.94‑1.16 for LPA). For subclinical atherosclerosis progression phenotypes, only GDF‑15 was significantly associated with both CAC progression (OR: 1.31, 95%CI: 1.10‑1.56) and plaque progression (OR: 1.25, 95%CI: 1.02‑1.52). LPA, NT‑proBNP, and PCSK9 showed no significant associations with either CAC progression (OR: 1.07, 0.92‑1.23; OR: 1.09, 0.93‑1.28; OR: 1.12, 0.96‑1.30, respectively) or plaque progression (OR: 1.05, 0.89‑1.25; OR: 1.13, 0.94‑1.35; OR: 1.11, 0.93‑1.32, respectively).

According to another reviewer’s suggestion, we newly detected hsTnI, a well-established biomarker to assess myocardial injury, in our two cohorts. Notably, hsTnI maintained non-zero coefficients in 90.6% of iterations of the LASSO model for CAC progression. Moreover, addition of hsTnI significantly improved the discriminative performance of the guideline-endorsed China-PAR score for incident ASCVD, with a ΔC-index of 0.009 (95% CI: 0.003–0.015). Accordingly, in the revised manuscript, we have constructed an updated ProtRS by integrating hsTnI. To ensure consistency in model construction, we reconstructed ProtRSs only using LASSO‑selected proteins in the revised manuscript, with the four previously preselected proteins removed entirely.

3. *In both Methods and Results the text give the impression that you have performed* *Mendelian randomization studies in the current paper. Please rephrase these sentences to make clear that you derive this information from the use of established databases. The databases and the way you use them needs a description in the appendix.*

**Response:** According to your suggestion, we have removed all MR-related content from the Methods and Results sections to eliminate any potential ambiguity, and integrated relevant MR information into the Discussion section (Page 14, line 376-379). We have explicitly clarified that we did not perform MR analyses in the current study; instead, we derived MR-related evidence from established public databases to support the potential causal roles of the identified proteins (*Nat Genet. 2020 Oct;52(10):1122-1131*). As requested, the databases and the way we use them were described in the appendix (Supplemental Table 11 and Supplemental Text).

To further strengthen our primary findings, we utilized logistic regression with a forward method to determine the associations between the proteins and SA progression, and conducted KEGG analysis to ensure a more focused biological interpretation.

4. *What is the relevance of a base-model using only age and sex (Supl Fig 5)? I suggest using a model with only risk factors as the basic model for comparison. If I understand correctly the risk thresholds has been recalibrated to the observed risk in the population and the fact that hemorrhagic stroke was excluded. You have also changed the decision thresholds. I think these changes make it difficult to evaluate the clinical value of the observed reclassification in relation to the original China-PAR score. I would suggest using the original 5% and 10% thresholds to facilitate interpretation.*

**Response:** Thank you for the suggestion. To enhance the interpretability of our results, we have replaced the base model containing only age and sex with a model that includes established risk factors as the reference model for comparison. We now presented AUCs of risk factors alone, ProtRSs alone and risk factors combined with ProtRSs for CAC and plaque progression. The risk factors referred to the variables in China-PAR risk model: baseline age, sex, urbanization, smoking status, waist circumference, systolic blood pressure, antihypertensive therapy, total cholesterol, high-density lipoprotein cholesterol, and diabetes (*Circulation. 2016 Nov 8;134(19):1430-1440*). We used Delong’s test to compare the AUC between the reference model (risk factors alone) and the model with additional ProtRSs. We observed a significant improvement of AUC after incorporating ProtRSs, with ΔAUC of 2.2% for CAC ProtRS with *P*-value of 0.0141 and ΔAUC of 3.6% for plaque ProtRS with *P*-value of 0.0003.

As you correctly noted, in our original manuscript we recalibrated the observed risk and adopted 4% and 8% as decision thresholds. To better evaluate the clinical value of reclassification relative to the original China-PAR score, we have now reanalyzed the prediction improvement by calculating the Net Reclassification Improvement (NRI) using the original 5% and 10% thresholds, as recommended. After revision, the overall NRI was 12.67% when adding ProtRSs to the China-PAR model, indicating that a substantial proportion of participants were correctly reclassified into clinically meaningful risk categories.

**Revision:** We updated the ROC curve analysis in Supplemental Figure 5 using the revised reference model. We added detailed descriptions of the risk factors included in the reference model and the calculation of Delong’s *P*-value in the Methods section. The revised NRI results and risk stratification based on 5% and 10% thresholds, which are now presented in Supplemental Table 10 and Figure 4, respectively.

**Supplemental Figure 5. Receiver-operating characteristics curve for the predictive performance of the ProtRS combined with traditional risk factors for SA progression.**

Figure legends: (A) Prediction of CAC progression. (B) Prediction of plaques progression. The risk factors consist of baseline age, sex, urbanization, smoking status, waist circumference, systolic blood pressure, antihypertensive therapy, total cholesterol, high-density lipoprotein cholesterol, and diabetes. The area under the curve (AUC) with its 95% confidence interval is presented in parentheses and incremental AUC improvements of risk factors with addition of protein risk scores to the risk factor model were assessed using Delong’s test. Abbreviations: CAC, coronary artery calcium; ProtRS, protein risk score; SA, subclinical atherosclerosis.

5. *I have difficulties to understand if you use the CACS or the carotid plaque ProtPRS for studies on reclassification (Fig 4b). Please clarify.*

**Response:** Both the protein risk scores (ProtRSs) for CAC and carotid plaque were employed in the risk stratification analysis presented in Fig 4b. Given that these two ProtRSs reflect distinct biological pathways underlying atherosclerotic progression, we combined them to define a unified protein risk category, following the methodology established in previous relevant literature (*Eur Heart J. 2023 Jan 14;44(3):221-231*). Specifically, the protein risk category was classified into three levels: high (high CAC ProtRS plus high plaque ProtRS), intermediate (either high CAC ProtRS or high plaque ProtRS), and low (low or intermediate CAC ProtRS together with low or intermediate plaque ProtRS). To avoid ambiguity, we have further clarified the definition and application of these protein risk scores in the footnotes of Table 3 and the legend of Figure 4 in the revised manuscript.

6. *The reclassification are selectively reported and need to be changed. The NRI is small 0.05. It is difficult to evaluate the information on reclassification by the text and figures provided. Please include a Table describing reclassification in the whole cohort as well as in cases and non-cases (similar to Tables used in Bergstrom JAMA 2025 or Paixao, JACC CVD Imaging 2015). This table would be a complement to Fig 4a. Important to calculate the net correct reclassification. I the high risk group, 135 individuals are classified to a higher risk category at the same time as 146 are classified into a lower category. This would result in net negative reclassification.*

**Response:** We greatly appreciate your constructive comments. In our original analysis, NRI was calculated using a two‑category risk stratification (low vs. high risk). Following your suggestion, we have now recalculated NRI using the standard three‑category risk stratification (low, intermediate, high risk) with the clinically accepted cut‑offs of 5% and 10%, yielding an updated overall NRI of 12.67%. As recommended, we have added a detailed reclassification table (Supplemental Table 10) that presents cross‑classification results for the entire cohort, as well as separately for cases and non‑cases, following the format and reporting standards used in previous influential studies (*JAMA. 2026 Jan 20;335(3):245-254.; JACC Cardiovasc Imaging. 2015 Nov;8(11):1285-93.*). Stratified by outcome status, the addition of ProtRS yielded an NRI of 14.79% (*P* < 0.001) among cases, but a net negative reclassification of −2.12% (*P* = 0.001) among non‑cases during the ≥10‑year follow‑up.

Notably, the intermediate‑risk group represents a clinically important population with high management uncertainty (*JAMA. 2012 Aug 22;308(8):788-95*). In this subgroup, ProtRS improved risk classification substantially, with an NRI of 41.62%. Specifically, 16.04% of cases and 25.58% of non‑cases in the intermediate‑risk group were correctly reclassified (both *P* = 0.009 and 0.008, respectively), highlighting the clinical value of ProtRSs in refining risk stratification for this key population.

**Revisions:** We described the reclassification in the whole cohort as well as in cases and non-cases in Supplemental Table 10. We have revised the Results and Discussion sections to clearly report net reclassification, including the net negative reclassification among non‑cases and the strong improvement in intermediate‑risk individuals (Page 13, line 330-337 and Page 15, line 416-421).

**Supplemental Table 10. Reclassification of risk categories of ASCVD comparing traditional risk scores with addition of protein risk scores.**

| Risk categories based on traditional risk score | Risk categories with addition of ProtRSs | | | |
| --- | --- | --- | --- | --- |
|  | Low risk | Intermediate risk | High risk | Total |
| *No. of all participants* |  |  |  |  |
| Low risk | 1298 | 128 | 24 | 1450 |
| Intermediate risk | 63 | 110 | 52 | 235 |
| High risk | 1 | 20 | 80 | 101 |
| Total | 1362 | 258 | 166 | 1786 |
| Increased risk |  |  |  | 204 |
| Decreased risk |  |  |  | 84 |
| NRI |  |  |  | 12.67% (*P*<0.001) |
| NRI in participants with intermediate risk |  |  |  | 41.62% (*P*<0.001) |
| *No. of participants experiencing an event within 10 years* | | | | |
| Low risk | 226 | 56 | 7 | 289 |
| Intermediate risk | 20 | 49 | 37 | 106 |
| High risk | 1 | 12 | 45 | 58 |
| total | 247 | 117 | 89 | 453 |
| Increased risk |  |  |  | 100 |
| Decreased risk |  |  |  | 33 |
| Net correctly reclassified (%) |  |  |  | 14.79% (*P*<0.001) |
| Net correctly reclassified in participants with intermediate risk (%) |  |  |  | 16.04% (*P*=0.009) |
| *No. of participants not experiencing an event when followed up for ≥ 10 years* | | | | |
| Low risk | 741 | 37 | 8 | 786 |
| Intermediate risk | 35 | 38 | 13 | 86 |
| High risk | 0 | 4 | 20 | 24 |
| Total | 776 | 79 | 41 | 896 |
| Increased risk |  |  |  | 58 |
| Decreased risk |  |  |  | 39 |
| Net correctly reclassified (%) |  |  |  | -2.12% (*P*=0.001) |
| Net correctly reclassified in participants with intermediate risk (%) |  |  |  | 25.58% (*P*=0.008) |

Notes: To calculate the net reclassification improvement of ten-year risk of ASCVD in case-cohort design, we excluded the cases that developed ASCVD after 10 years, and participants developed ASCVD after 10 years in sub-cohort were considered as non-cases. The traditional risk score was the sex-specific China-PAR risk score includes age, geographic region, urbanization (only for men), systolic blood pressure, antihypertensive therapy, total cholesterol, high-density lipoprotein cholesterol, waist circumference, current smoking, diabetes mellitus and family history of ASCVD (only for men), as well as interactions of age with some variables. ProtRSs include both CAC and plaque ProtRS. Risk categories were defined as low risk (<5%), intermediate risk (5% to 10%), and high risk (>=10%). Abbreviations: ASCVD, atherosclerotic cardiovascular disease; CAC, coronary artery calcium; China-PAR, Prediction for Atherosclerotic cardiovascular disease Risk in China; ProtRS, protein risk scores; NRI, net reclassification improvement.

7. *Results are much exaggerated in the discussion. I do not think this statement has any relation to data "the ProtRSs provided critical clinical utility by reclassifying high-risk subgroups.". As it stand now (without reclassification tables) I would say the ProtRS provide a modest recalibration of risk (AUC 0.02 and NRI 0.05).*

*In my mind, existing research has established a modest value of protein risk scores in primary prevention. The following statement needs to be rephrased. : "existing research has established the predictive superiority of plasma protein biomarkers for cardiovascular risk prediction(49-52)."*

*In addition, refences (49-52) are also mixed between primary and secondary prevention as well as heart failure. Only the reference by Hoogeveen relates to primary prevention. You could use the following reference that is very clos to what you have done: Royer, Eur J Prev Cardiol 2024 Vol. 31 Issue 14 Pages 1681-1689.*

*The following statement must also include how many individuals were incorrectly classified into a lower category and was missed from prevention. "Our findings addressed this knowledge gap by demonstrating that the ProtRSs reclassified 18.9% (93/492) of guideline-defined intermediate-risk individuals into the high-risk category, where nearly half of them (46/93, 49.5%) indeed experienced subsequent ASCVD events."*

**Response:** We greatly appreciate your careful and constructive comments, which have helped us significantly improve the objectivity, clarity, and completeness of our manuscript. We have carefully revised the manuscript to ensure balanced interpretation of our findings, appropriate citation of literature focused on primary prevention, and full reporting of reclassification data as suggested.

First, we have revised all relevant interpretations to avoid overstatement and ensure our conclusions are objectively supported by the data. Consistent with the modest incremental improvement observed for the ProtRSs, we have toned down claims regarding their clinical utility throughout the manuscript. Instead of emphasizing “critical clinical value”, we now describe the ProtRSs as providing modest but meaningful incremental value for risk stratification. As recommended, we recalculated NRI using the 5% and 10% thresholds, resulting in an overall NRI of 12.67% in the total cohort and a marked NRI of 41.62% among intermediate-risk individuals, supporting the potential value of the ProtRSs in refining risk stratification. In line with your comment that previous studies support only modest improvements from protein biomarkers in primary prevention, we revised the relevant statement to: “Previous proteomic studies have explored the utility of circulating protein biomarkers and found modest improvement when adding plasma proteins to traditional risk scores in primary prevention setting.” We have also carefully adjusted the entire Discussion section to ensure all claims are evidence-based and do not overinterpret the clinical implications of the ProtRSs.

Second, we sincerely appreciate the valuable references you recommended. We have revised our reference list to eliminate studies involving mixed populations (including secondary prevention and heart failure) and ensure all citations focus on primary prevention. Specifically, we removed references 49–52 and added the suggested study by Royer et al. (*Eur J Prev Cardiol 2024, 31(14):1681–1689*), which is highly consistent with our study design. Furthermore, we added a brief comparison between our findings and those of Royer et al. in the Discussion section to better contextualize our results within the existing literature on primary prevention.

Third, to address the incomplete reporting of reclassification, we supplemented the description with the number of participants incorrectly reclassified to a lower risk category and thus potentially missed for preventive interventions. The revised sentence in the Results section now states: “Notably, among participants with intermediate risk, the overall NRI for ProtRSs reached 41.62% (P<0.001), with 16.04% (*P*=0.009) for cases, and 25.58% (*P*=0.008) for non-cases. Specifically, ProtRSs correctly unclassified 34.91% (37/106) of future ASCVD events from intermediate to high risk, while 18.9% (20/106) of cases were erroneously downclassified from intermediate to low risk.” We also explicitly discussed this limitation in the Discussion, noting that although the ProtRSs help identify high-risk individuals within the intermediate-risk group, it also incorrectly reclassified some future cases from intermediate to low risk. The ProtRSs may serve as risk enhancers to further refine clinical risk assessment, particularly for individuals for whom traditional calculators may underestimate actual risk.
