# Supplementary material for: Plasma proteomic signatures of subclinical atherosclerosis progression for cardiovascular risk stratification: A cohort study
Source: PLoS Med. 2026 Oct 20;23(10):e1005265. doi: 10.1371/journal.pmed.1005265 (PMC13682661; doi:10.1371/journal.pmed.1005265)
Supplement: Supplementary file 7 [file pmed.1005265.s010.docx]

**Editor’s comments:**

*This study tackles a critical scientific question in cardiovascular risk prediction. Current risk models in many countries have reached a plateau, and the integration of plasma proteomic signatures is a timely and promising frontier. Notably, shifting the endpoint from hard cardiovascular events to measures of subclinical atherosclerosis progression is a thoughtful and clinically relevant approach.*

**Response:** Thank you for your thorough review and valuable comments on our manuscript. We have addressed all concerns of four Reviewers and generally requirements of editors in a point-by-point manner, and we believe that the manuscript has been improved after revision.

**Reviewers' comments:**

**Reviewer #1:**

*The authors have addressed all of my questions, and I have no additional comments.*

**Response:** We sincerely appreciate all your comments.

**Reviewer #2:**

*The manuscript is improved and much better balanced.*

*Note, NRI is the sum of differences in proportions and is thus not expressed as a percentage. This needs to be corrected throughout.*

**Response:** Thank you for pointing this out. We have revised the NRI values (both overall NRI and NRI in participants with intermediate risk) to decimals throughout the manuscript.

**Reviewer #3:**

*I appreciate the effort made by the authors to address every one of the raised points (not only mine but also from the other reviewers). The authors have adequately addressed all my points except points 1 and 5.*

**Response:** We deeply appreciate you for constructive comments. We have further addressed the two points in this version, and we believe that the manuscript has been improved after revision.

1. *Point 1.- the authors performed a stepwise logistic regression to select a subset of proteins independently and significantly associated to atherosclerosis (plaque and CAC), which was the initial idea. However, they used Akaike information criterion for candidate selection. While this procedure is adequate to construct a biomarker panel, again it does not demonstrate significant association of all the retained proteins. I would accept the result provided that throughout the manuscript the authors eliminate the wording "significantly associated with plaque/CAC" and consider the list of proteins as those that "configure an optimal panel for predicting the cases" or similar wording.*

*The authors claim that the proteins associated with CAC progression were "enriched in lipid and atherosclerosis pathways". This is truly incorrect; from inspecting Supp Table 6 there is no evidence for such an affirmation; just the contrary, the majority of proteins in these pathways are associated to plaque and not CAC progression. This must be corrected in the results section to make them more consistent with the much more adequate interpretation made in the Discussion, where each protein is interpreted separately.*

*I have difficulties in understanding new results from Fig. 3. For instance in the case of plaque progression (right panel), why the proteins KFBP-2, MMP3, TMP4, HP, MCP1 are not there? Why are there proteins that are both not significant when the idea is to plot the proteins that are significant in plaque progression or ASCVD? Similar concerns apply to the left panel (CAC progression)*

**Response:** Thank you for pointing this out. As you mentioned, proteins retained in stepwise logistic regression based on Akaike information criterion can improve model fit, but this does not equate to a significant association with SA progression. According to your suggestion, we revised all the relevant description in our manuscript and removed inappropriate statements regarding statistical association. The revised text reads as follows: “We further conducted forward stepwise logistic regression to identify the proteins with potential biological relevance to atherosclerosis progression for establishing an optimal prediction panel. In the final models, 23 and 19 proteins were retained for CAC and plaque progression, respectively.”

We agree that the descriptions of the KEGG pathway analysis results were inappropriate, as in the Supplemental Table 6, a subtly distinct set of proteins in the optimized protein panel for CAC progression and plaque regression are implicated in lipid metabolism and atherogenic pathways. Therefore, we have pointed out some phenotype-specific proteins linked to lipid and atherosclerosis in the Results. The updated Results states that “Subsequent KEGG pathway analysis revealed that a set of proteins within the optimal panel for plaque progression (e.g., C-C motif chemokine 24 [CCL-24] and interleukin-8 [IL-8]) were primarily implicated in inflammatory processes, including cytokine signaling and NF-κB activation. Several proteins within the optimal panel for CAC progression were included in pathway linked to lipid and atherosclerosis (e.g., low-density lipoprotein receptor), whereas a subtly different protein set for plaque progression was mapped to the same pathways (e.g., monocyte chemotactic protein 1 [MCP-1]).” And in the revised Discussion, we provided interpretations for each protein that align with the Results (Page 15, Line 407-410).

To further clarify the rationale supporting the predictive capacity of SA ProtRSs for incident ASCVD, we examined whether proteins comprising ProtRSs exerting stronger effects on SA progression would confer a higher risk of incident ASCVD. Thus, all proteins displayed in Figure 3 were identified via LASSO regression and incorporated into the SA ProtRSs. Proteins selected via forward logistic regression but not incorporated into the ProtRSs (such as HP, ICAM-2, IGFBP-2, TIMP4, MCP-1, and MMP-3) were excluded from the analysis. While the majority of proteins (92.9%) exhibited directional consistency, LASSO-selected proteins are not necessarily significantly associated with SA progression. To avoid vague descriptions, we have emphasized in the figure legend and related text that these proteins were identified by LASSO regression.

2. *Point 5.- the results obtained using the protRS scores for predicting ASCVD are not compared with those obtained with CV risk factors in statistical terms. I mean that there are no p-values in Table 3 reflecting the significance of adding protRS scores to the CV risk scores. Note that these p-values are actually provided for the prediction of plaque and CAC progression; why are they not there for ASCVD?*

**Response:** Thank you for the valuable suggestion. Previously, we reported the improvement of predictive performance for ASCVD with addition to ProtRSs without *P*-values, as the 95% CI for the improvement of Harrell's C-index was widely presented by existing studies (*JAMA. 2023 Aug 22;330(8):725-735; JAMA. 2016 Jun 21;315(23):2532-41*). The *p* value for the C-index improvement after incorporating ProtRSs into CV risk scores was 0.011, which aligned with the 95% CI for the C-index improvement: 0.018 (0.003 - 0.035). Following your suggestion, we included the *p* values in Table 3 and relevant text to help readers interpret the results.

**Reviewer #4:**

*Thanks for the revised manuscript and responses to my original review. The additional material resolved many of my original queries, there are three issues that I think can be resolved with minor revision.*

**Response:** We deeply appreciate your constructive suggestions. We have revised our manuscript according to your suggestions.

1. *I think the calibration plots are a good addition - these demonstrate that the CAC and plaques progression have reasonable levels of calibration overall (with some positive bias around the medium risk zone for plaque progression). The Homser-Lemeshow tests are unnecessary here - the bins are arbitrary and this test often reflects sample size rather than any important miscalibration. The important information is contained in the calibration plot itself.*

**Response:** We appreciate your constructive suggestion. As you noted, we found that the recent *Scientific Statement From the American Heart Association* also stated that the use of the Hosmer-Lemeshow goodness-of-fit test is widely discouraged due to its limited power and poor interpretability (*Circulation. 2026 Mar 17;153(11):e953-e970*). Therefore, according to your suggestion, we removed Homser-Lemeshow tests from Supplemental Figure 6 and our manuscript (Page 13, Line 224-226; Page 12, Line 338-340; Supplemental Figure 5).

2. *In addition to DeLong's test, I would also add a likelihood ratio test (which tests model fit and parsimony)- this is more powerful to DeLong, and there are some circumstances in which the assumptions of the DeLong test are not appropriate (see* [*https://doi.org/10.1002/sim.5328*](https://doi.org/10.1002/sim.5328)*)*

**Response:** We really appreciate the method and literature you provided. As Demler OV et al. pointed out, DeLong’s test would loss of power under certain circumstances (*Stat Med. 2012 Oct 15;31(23):2577-87*). In comparison, the likelihood ratio test for model fit is suitable and robust for comparing the difference between two nested models. Following your suggestion, we conducted likelihood ratio tests of models of risk factors alone and risk factors combing with ProtRSs for CAC and plaque progression. We observed that there were significant improvement of model fit after adding ProtRSs, with *P*-value of 6.7×10^-8^ and 4.9×10^-12^ for CAC and plaque progression, respectively.

**Revision:** We have presented the results of likelihood ratio test in the manuscript (Page 13, Line 335-337).

3. *Supp Table 2. I think that a test for difference in baseline characteristics is not useful in this table - the important information about levels of difference in each variable between non-progressors and progressors can be seen from the summary statistics.*

**Response:** Thank you for the suggestion. We have removed the tests for difference in baseline characteristics in Supplemental Table 2 and in the manuscript (Page 11, Line 273-276).

**General editorial requests:**

1. *Please upload any figures associated with your paper as individual TIF or EPS files with 300dpi resolution at resubmission; please read our figure guidelines for more information on our requirements: http://journals.plos.org/plosmedicine/s/figures. While revising your submission, we strongly recommend that you use PLOS's NAAS tool (https://ngplosjournals.pagemajik.ai/artanalysis) to test your figure files. NAAS can convert your figure files to the TIFF file type and meet basic requirements (such as print size, resolution), or provide you with a report on issues that do not meet our requirements and that NAAS cannot fix.*

*After uploading your figures to PLOS's NAAS tool - https://ngplosjournals.pagemajik.ai/artanalysis, NAAS will process the files provided and display the results in the "Uploaded Files" section of the page as the processing is complete.*

*If the uploaded figures meet our requirements (or NAAS is able to fix the files to meet our requirements), the figure will be marked as "fixed" above. If NAAS is unable to fix the files, a red "failed" label will appear above.*

*When NAAS has confirmed that the figure files meet our requirements, please download the file via the download option, and include these NAAS processed figure files when submitting your revised manuscript.*

**Response:** Thank you for providing the valuable tool. We have uploaded the figures with 300dpi resolution, and included these NAAS processed figure files when submitting our revised manuscript.

2. *Please ensure that the paper adheres to the PLOS Data Availability Policy (see http://journals.plos.org/plosmedicine/s/data-availability), which requires that all data underlying the study's findings be provided in a repository or as Supporting Information. For data residing with a third party, authors are required to provide instructions with contact information (web or email address) for obtaining the data. Please note that a study author cannot be the contact person for the data. PLOS journals do not allow statements supported by "data not shown" or "unpublished results." For such statements, authors must provide supporting data or cite public sources that include it.*

**Response:** We have provided the minimal dataset for this study in Supporting Information - S1 Data.

3. *Data availability statement: Thank you for including the minimal data set. However, there is not sufficient information in your data availability statement. Please include where the original data can be found, and whether these are freely available. If they are not freely available, include reasons why, and contact details (email/URL) where researchers could request access.*

**Response:** The minimal dataset for this study was provided in S1 Data. The original data from the Beijing Community-Based Cohort of Atherosclerosis Study, International Collaborative Study of Cardiovascular Disease in Asia (InterASIA), and the China Multi-Center Collaborative Study of Cardiovascular Epidemiology (ChinaMUCA) cohorts are subject to ethics committee and are not publicly available. The original data will be made available to other researchers upon request to Ethics Committee of Fuwai Hospital (fuwailunli@fuwai.com). The request for data sharing will be reviewed and approved by the research management group and the Ethics Committee of Fuwai Hospital.

4. *We expect all researchers with submissions to PLOS in which author-generated code underpins the findings in the manuscript to make all author-generated code available without restrictions upon publication of the work. In cases where code is central to the manuscript, we require the code to be made available as a condition of publication. Authors are responsible for ensuring that the code is reusable and well documented. Please make any custom code available, either as part of your data deposition or as a supplementary file. Please add a sentence to your data availability statement regarding any code used in the study, e.g. "The code used in the analysis is available from Github [URL] and archived in Zenodo [DOI link]" Please review our guidelines at https://journals.plos.org/plosmedicine/s/materials-software-and-code-sharing and ensure that your code is shared in a way that follows best practice and facilitates reproducibility and reuse. Because Github depositions can be readily changed or deleted, we encourage you to make a permanent DOI'd copy (e.g. in Zenodo) and provide the URL.*

**Response:** We have provided the author-generated code in Supporting information - S1 Code, and the code is reusable and well documented.

5. *Financial statement: Thank you for including your funding and the grant numbers. Please also include the initials of the researchers that were awarded the grant, as well as URLs to the funders' websites.*

**Response:** We have added the initials of the researchers that were awarded the grant, as well as URLs to the funders' websites.

6. *The funding statement should include: specific grant numbers, initials of authors who received each award, URLs to sponsors’ websites. Also, please state whether any sponsors or funders (other than the named authors) played any role in study design, data collection and analysis, the decision to publish, or preparation of the manuscript. If they had no role in the research, include this sentence: “The funders had no role in study design, data collection and analysis, decision to publish, or preparation of the manuscript.”*

**Response:** We have stated that: “The funders had no role in study design, data collection and analysis, decision to publish, or preparation of the manuscript.”

7. *Please disclose any use of AI tools and technologies to the study or article content at the end of the Methods. Please specify the name(s) and version(s) of any tool(s) used and a description of their use, including how the AI-assisted or generated content was evaluated by authors. Please also include a statement specifying who retains responsibility for the final manuscript. Please see our guidelines for more information:* [*https://journals.plos.org/plosmedicine/s/ethical-publishing-practice#loc-artificial-intelligence-tools-and-technologies*](https://journals.plos.org/plosmedicine/s/ethical-publishing-practice#loc-artificial-intelligence-tools-and-technologies)*.*

**Response:** No AI tools were used in this study to generate data, results or contents. All authors take full responsibility for the final version of the manuscript.

8. *Please ensure that all abbreviations are defined at first use throughout the text.*

**Response:** We have carefully checked the revised manuscript and ensured that all abbreviations are defined at first use throughout the text.

9. *Please confirm that all numbers presented in the abstract are present and identical to numbers presented in the main manuscript text.*

**Response:** We have carefully checked all numerical values reported in the Abstract and confirmed that they are presented and identical to the corresponding numbers reported in the main manuscript text.

**FORMATTING - GENERAL:**

1. *Please confirm that your title complies with PLOS Medicine's style. Your title must be nondeclarative and not a question. It should begin with main concept if possible. "Effect of" should be used only if causality can be inferred, i.e., for an RCT. Please place the study design ("A randomized controlled trial," "A retrospective study," "A modelling study," etc.) in the subtitle (ie, after a colon).*

**Response:** We have confirmed that the title to comply with PLOS Medicine’s style requirements.

2. *In the last sentence of the Abstract Methods and Findings section, please describe the main limitation(s) of the study's methodology.*

**Response:** We have revised the final sentence of the Abstract Methods and Findings section to describe the main methodological limitation of the study.

3. *At this stage, we ask that you include a short, non-technical Author Summary of your research to make findings accessible to a wide audience that includes both scientists and non-scientists. The Author Summary should immediately follow the Abstract in your revised manuscript. This text is subject to editorial change and should be distinct from the scientific abstract. Ideally each sub-heading should contain 2-3 single sentence, concise bullet points containing the most salient points from your study. In the final bullet point of 'What Do These Findings Mean?', please include the main limitations of the study in non-technical language. Please see our author guidelines for more information:* [*https://journals.plos.org/plosmedicine/s/revising-your-manuscript#loc-author-summary*](https://journals.plos.org/plosmedicine/s/revising-your-manuscript#loc-author-summary)*.*

**Response:** We have added a short, non-technical Author Summary immediately following the Abstract in the revised manuscript.

4. *Please express the main results with 95% CIs as well as p values. When reporting p values please report as p<0.001 and where higher as the exact p value p=0.002, for example. Throughout, suggest reporting statistical information as follows to improve clarity for the reader "22% (95% CI [13%,28%]; p</=)". Please be sure to define all numerical values at first use.*

**Response:** Thank you for the comment. We have revised the reporting formats of 95% confidence intervals and *p* values where appropriate. We have also checked the manuscript to ensure that all numerical values are defined at first use and that statistical information is presented consistently and clearly.

5. *Please cite the reference numbers in square brackets. Citations should precede punctuation.*

**Response:** Thank you for this comment. We have revised the reference formatting throughout the manuscript.

**FIGURES AND TABLES**

6. *Please consider avoiding the use of red and green in order to make your figure more accessible to those with color blindness. Please also check this for supplemental figures.*

**Response:** As suggested, we have revised all figures and supplementary figures to avoid combining red and green colors. This modification ensures the figures are accessible to individuals with color blindness.

7. *Please define all elements of box plots in the figure caption - center line, box limits and whiskers.*

**Response:** We have supplemented the definitions of all box plot components including the center line, box limits and whiskers in the Figure 2’s caption.

8. *When a p value is given, please specify the statistical test used to determine it in the legend.*

**Response:** We have specified the statistical test used to generate *p* values in the legend of all figures and tables.

9. *Please consider if moving some of the figures shared in the supplementary information into the main text would aid the reader. Specifically, please consider moving Figure S1 into the main text.*

**Response:** Thank you for your suggestion. We have moved Figure S1 into the main text.

**SUPPLEMENTARY MATERIAL**

10. *Please note that supplementary material will be posted as supplied by the authors. Therefore, please amend it according to the relevant comments outlined here.*

**Response:** Thank you for your suggestion. We have carefully reviewed and revised the supplementary material according to the relevant comments.

11. *Please cite your Supporting Information as outlined here: https://journals.plos.org/plosmedicine/s/supporting-information*

**Response:** We have revised the citations to the Supporting Information throughout the manuscript in accordance with PLOS Medicine requirements.

**REFERENCES**

12. *PLOS uses the numbered citation (citation-sequence) method and first six authors, et al.*

**Response:** We have revised the citations’ formats.

13. *Please ensure that journal name abbreviations match those found in the National Center for Biotechnology Information (NCBI) databases (http://www.ncbi.nlm.nih.gov/nlmcatalog/journals), and are appropriately formatted and capitalised.*

**Response:** Thank you for your comment. We have checked and revised journal name abbreviations to ensure that they match those listed in the NCBI databases and are appropriately formatted and capitalized.

14. *Where website addresses are cited, please include the complete URL and specify the date of access (e.g. [accessed: 12/06/2023]).*

**Response:** Thank you for your comment. We have checked website citations and added complete URLs and dates of access where applicable.

15. *Please also see https://journals.plos.org/plosmedicine/s/submission-guidelines#loc-references for further details on reference formatting.*

**Response:** Thank you for your comment. We have reviewed the PLOS Medicine reference formatting guidelines and revised the reference list accordingly.

**OBSERVATIONAL STUDIES**

16. *Abstract: Please include the study design, population and setting, number of participants, years during which the study took place (enrollment and follow up), length of follow up, and main outcome measures.*

**Response:** We have revised the abstract to include the contents above.

17. *Please ensure that the study is reported according to the STROBE (or appropriate STOBE extension) guideline (available from: https://www.equator-network.org/reporting-guidelines/strobe) and include the completed STROBE (or STROBE extension) checklist as Supporting Information. Please add the following statement, or similar, to the Methods: "This study is reported as per the Strengthening the Reporting of Observational Studies in Epidemiology (STROBE) guideline (S1 Checklist)." When completing the checklist, please use section and paragraph numbers, rather than page numbers.*

**Response:** We have included the completed STROBE checklist as Supporting Information – S1 checklist. And we have added the statements in method section in manuscript: "This study is reported as per the Strengthening the Reporting of Observational Studies in Epidemiology (STROBE) guideline (S1 Checklist)."

18. *Please also ensure that the study is reported according to the TRIPOD guideline (https://www.tripod-statement.org/) and include the completed TRIPOD checklist as Supporting Information. Please add the following statement, or similar, to the Methods: "This study is reported as per the Transparent Reporting Of a Multivariate Prediction Model (TRIPOD) guideline (S1 Checklist)." When completing the checklist, please use section and paragraph numbers, rather than page numbers.*

**Response:** We have included the completed TRIPOD checklist as Supporting Information – S2 checklist. And we have added the statements in method section in manuscript: " This study is reported as per the Transparent Reporting Of a Multivariate Prediction Model (TRIPOD) guideline (S2 Checklist)."

19. *For all observational studies, in the manuscript text, please indicate: (1) the specific hypotheses you intended to test, (2) the analytical methods by which you planned to test them, (3) the analyses you actually performed, and (4) when reported analyses differ from those that were planned, transparent explanations for differences that affect the reliability of the study's results. If a reported analysis was performed based on an interesting but unanticipated pattern in the data, please be clear that the analysis was data driven.*

**Response:** We have indicated the hypotheses of the study, and performed statistical analysis based on a prospective protocol of cohorts.

20. *Please state in the Methods section whether the study had a prospective protocol or analysis plan. If a prospective analysis plan (from your funding proposal, IRB or other ethics committee submission, study protocol, or other planning document written before analyzing the data) was used in designing the study, please include the relevant document(s) with your revised manuscript as a Supporting Information file to be published alongside your study and cite it in the Methods section. A legend for this file should be included at the end of your manuscript. If no such document exists, please make sure that the Methods section transparently describes when analyses were planned, and when/why any data-driven changes to analyses took place. Changes in the analysis, including those made in response to peer review comments, should be identified as such in the Methods section of the paper, with rationale.*

**Response:** We have provided a prospective protocol of cohorts in S1 Text and cited it in the Methods section.
