# Supplementary material for: Plasma proteomic signatures of subclinical atherosclerosis progression for cardiovascular risk stratification: A cohort study
Source: PLoS Med. 2026 Oct 20;23(10):e1005265. doi: 10.1371/journal.pmed.1005265 (PMC13682661; doi:10.1371/journal.pmed.1005265)
Supplement: Supplementary file 8 [file pmed.1005265.s011.docx]

**Reviewers' comments:**

**Reviewer #3:**

*The authors have addressed my concerns about the wording (eliminate association) and the proteins in Fig. 3 and also in point 5. However, they have not resolved my concern about the interpretation of the KEGG enrichment analysis. There are several sentences that are incorrect:*

**Response:** We sincerely appreciate your constructive comments. We have further clarified the interpretation of the KEGG enrichment analysis in the revised manuscript and believe it has been improved.

1. *"Subsequent KEGG pathway analysis revealed that a set of proteins within the optimal panel for plaque progression (e.g., C-C motif chemokine 24 [CCL-24] and interleukin-8 [IL-8]) were primarily implicated in inflammatory processes, including cytokine signaling and NF-κB activation. Several proteins within the optimal panel for CAC progression were included in pathway linked to lipid and atherosclerosis (e.g., low-density lipoprotein receptor), whereas a subtly different protein set for plaque progression was mapped to the same pathways (e.g., monocyte chemotactic protein 1 [MCP-1])."*

*These sentences are completely misleading, since although there are some proteins for plaque progression implicated in inflammatory processes there are also many proteins for CAC progression also implicated in the same processes (namely four out of 10 in the cytokine category and two out of 4 in the NF-KB category come from CAC progression). And in the lipid and atherosclerosis category the majority are from plaque progression (five out of six!) and not from CAC progression. This is not a subtly different set, the truth is that both kind of proteins (for plaque and for CAC) are implicated in all these categories. Highlighting that plaque proteins are mostly associated to some categories and CAC proteins to others is completely misleading the reader towards a biased interpretation not supported by the data.*

*"Meanwhile, phenotype-specific protein signatures were also identified: plaque progression was predominantly associated with inflammatory mediators including IL-8 and CCL-24 [38, 39], whereas CAC progression was linked to atherosclerosis through low-density lipoprotein receptor, a key regulator of cholesterol homeostasis that drives atherogenesis [40]"*

*This sentence is not only misleading but completely false, since the authors claim that the "specificity" of the protein signatures is vinculated to inflammation in the case of plaque and atherosclerosis in the case of CAC. As commented above, both kind of proteins are associated to both inflammation and atherosclerosis, hence this assertion on specificity is false.*

**Response:** We sincerely appreciate your constructive comments, and we have therefore removed all statements suggesting such specificity and used the KEGG findings to demonstrate that protein signatures for CAC and plaque progression converge on these shared biological processes.

In the revised manuscript, the Results section now reads: " Subsequent KEGG pathway analysis revealed that a set of proteins within the optimal panel for plaque progression (e.g., C-C motif chemokine 24 [CCL-24] and interleukin-8 [IL-8]) and those for CAC progression (e.g., tumor necrosis factor receptor 2 [TNF-R2] and urokinase-type plasminogen activator [uPA]) were linked to inflammatory processes, including cytokine signaling and NF-κB activation. Other proteins, such as low-density lipoprotein receptor within the CAC progression panel and monocyte chemotactic protein 1 (MCP-1) within plaque progression panel, were involved in lipid metabolism and atherosclerosis (S6 Table)." (Page 12, Line 305-311)

In the Discussion, we have revised the text to: "Meanwhile, KEGG pathway analysis further demonstrated that protein signatures for plaque progression and CAC progression converged on inflammation and cholesterol homeostasis regulation pathways that drive atherogenesis, including IL-8, CCL-24, MCP-1, and low-density lipoprotein receptor [38-41]." (Page 15, Line 410-413)

We deeply appreciate your suggestion, which has significantly improved the accuracy and rigor of our interpretation.

**Reviewer #4:**

*Thanks for the revised manuscript and responses to my original review. The revised manuscript and responses to my questions have resolved my original questions.*

**Response:** We sincerely appreciate all your comments.

**General editorial requests:**

1. *Thank you for including the limitation in your abstract and Author summary. However, I don't agree that the lack of cost-effectiveness is the main limitation. Please change the Abstract and the Author summary to include the main limitation of this study (e.g. no external validation).*

**Response:** Thank you for the suggestion. We have revised the main limitation statement in both the Abstract and the Author summary to read: “The main limitation is that the predictive value of ProtRSs warrants further large-scale external validation.”

2. *When a p value is given, please specify the statistical test used to determine it in the legend.*

**Response:** We have added the statistical test used to determine *p* values to all tables, figures, and appendix legends.

3. *Thank you for stating your study had a prospective* *analysis plan and including it. Please also ensure all changes to the analysis-- including those made in response to peer review comments-- are identified as such in the Methods section of the paper, with rationale.*

**Response:** Thank you for your suggestion. In the revised manuscript, we have incorporated supplemental analyses of model fit, calibration, and several sensitivity analyses according to the reviewers’ comments. We have also newly measured plasma troponin levels for inclusion in the protein panel and corrected several statistical issues in the NRI and AUC calculations. All these changes, along with their rationale, are documented in the newly added section “Changes to the statistical analysis in response to reviewers” in S1 Text.

4. *Please confirm that your title complies with PLOS Medicine's style. Your title must be nondeclarative and not a question. It should begin with main concept if possible. "Effect of" should be used only if causality can be inferred, i.e., for an RCT. Please place the study design ("A randomized controlled trial," "A retrospective study," "A modelling study," etc.) in the subtitle (ie, after a colon).*

**Response:** Thank you for pointing this out. We revised our title to be nondeclarative: “Plasma proteomic signatures of subclinical atherosclerosis progression for cardiovascular risk stratification: A prospective cohort study”.

5. *Please include a statement regarding use or non-use of AI tools and technologies at the end of the Methods. Please specify the name(s) and version(s) of any tool(s) used and a description of their use, including how the AI-assisted or generated content was evaluated by authors, and a clear statement of which aspects of the study, article contents, data, or supporting files were affected or generated by AI tool usage. Please also include a statement specifying who retains responsibility for the final manuscript. See our guidelines for more information: https://journals.plos.org/plosmedicine/s/ethical-publishing-practice#loc-artificial-intelligence-tools-and-technologies.*

**Response:** We have included an Artificial intelligence statement at the end of the Methods section: “No artificial intelligence tools or technologies were used to generate, edit, or analyze the study, data, or article content. The authors take full responsibility for the final manuscript.”

6. *The Data Availability Statement (DAS) requires revision. I appreciate your statement that the data are not freely available. Please describe the restrictions for data access in more detail.*

*C) If the data are not freely available, please describe briefly the ethical, legal, or contractual restriction that prevents you from sharing it. Please also include an appropriate institutional contact (institutional web or email address) for inquiries (again, this cannot be a study author).*

**Response:** Thank you for your suggestion. We have revised the relevant text to: “The minimal dataset for this study was provided in S1 Data. The original data from the Beijing Community-Based Cohort of Atherosclerosis Study, International Collaborative Study of Cardiovascular Disease in Asia (InterASIA), and the China Multi-Center Collaborative Study of Cardiovascular Epidemiology (ChinaMUCA) cohorts cannot be publicly shared due to a need to maintain the confidentiality of patient data. The original data will be made available to other researchers upon request to Ethics Committee of Fuwai Hospital (fuwailunli@fuwai.com). The request for data sharing will be reviewed and approved by the research management group and the Ethics Committee of Fuwai Hospital. We have also provided the author-generated code in S1 Code.”
