# Supplementary material for: Plasma proteomic signatures of subclinical atherosclerosis progression for cardiovascular risk stratification: A cohort study
Source: PLoS Med. 2026 Oct 20;23(10):e1005265. doi: 10.1371/journal.pmed.1005265 (PMC13682661; doi:10.1371/journal.pmed.1005265)
Supplement: Peer review history

# Author comment: Author response to Decision Letter 0

**DOI:** 10.1371/journal.pmed.1005265.r001

22 Apr 2026

Attachment Submitted filename: Response Letter.docx

# Editor report: Decision Letter 0

**DOI:** 10.1371/journal.pmed.1005265.r002

**Authors:** Suzanne De Bruijn

30 Apr 2026

Dear Dr Lu,

Thank you for submitting your manuscript entitled "Plasma Proteomic Signatures Driving Subclinical Atherosclerosis Progression and Their Potential for Cardiovascular Risk Stratification" for consideration by PLOS Medicine.

Your manuscript has now been evaluated by the PLOS Medicine editorial staff, and I am writing to let you know that we would like to send your submission out for external peer review. We will attempt to recruit the original reviewers.

However, before we can send your manuscript to reviewers, we need you to complete your submission by providing the metadata that is required for full assessment. To this end, please login to Editorial Manager where you will find the paper in the 'Submissions Needing Revisions' folder on your homepage. Please click 'Revise Submission' from the Action Links and complete all additional questions in the submission questionnaire.

For clinical studies, please upload a copy of your trial study protocol as a supporting information file. The study protocol should be the version submitted for approval to the institutional review board or ethics committee, should include any amendments to the study protocol, as well as the date of their approval by the institutional review or ethics committee. Please also detail any deviations from the study protocol in the Methods section of your manuscript. The editors will consider the protocol and study conduct prior to a final decision for external review.

Please re-submit your manuscript within two working days, i.e. by May 04 2026 11:59PM.

Login to Editorial Manager here: https://www.editorialmanager.com/pmedicine

Once your full submission is complete, your paper will undergo a series of checks in preparation for peer review. Once your manuscript has passed all checks it will be sent out for review.

Feel free to email us at plosmedicine@plos.org if you have any queries relating to your submission.

Kind regards,

Suzanne De Bruijn, PhD

Associate Editor

PLOS Medicine

# Author comment: Author response to Decision Letter 1

**DOI:** 10.1371/journal.pmed.1005265.r003

1 May 2026

Attachment Submitted filename: Response_Letter_auresp_1.docx

# Peer review: Decision Letter 1

**DOI:** 10.1371/journal.pmed.1005265.r004

**Authors:** Suzanne De Bruijn

8 Jun 2026

Dear Dr Lu,

Many thanks for submitting a revised version of your manuscript "Plasma Proteomic Signatures Driving Subclinical Atherosclerosis Progression and Their Potential for Cardiovascular Risk Stratification" (PMEDICINE-D-26-01373R1) to PLOS Medicine. The paper has been reviewed by all of the original reviewers; their comments are included below and can also be accessed here: [LINK]

As you will see, the reviewers thought the manuscript has much improved, but have a few remaining concerns. After discussing the paper with the editorial team and an academic editor with relevant expertise, I'm pleased to invite you to revise the paper in response to the reviewers' comments. We plan to send the revised paper to some or all of the original reviewers, and we cannot provide any guarantees at this stage regarding publication.

In addition to these revisions, please address the editorial requests which you can find at the bottom of this email, and provide a point-by-point response to these concerns when submitting your revision. Furthermore, you may need to complete some formatting changes, which you will receive in a follow up email. A member of our team will be in touch with a set of requests shortly. If you do not receive a separate email within a few days, please assume that checks have been completed, and no additional changes are required.

When you upload your revision, please include a point-by-point response that addresses all of the reviewer and editorial points, indicating the changes made in the manuscript and either an excerpt of the revised text or the location (eg: page and line number) where each change can be found. Please also be sure to check the general editorial comments at the end of this letter and include these in your point-by-point response. When you resubmit your paper, please include a clean version of the paper as the main article file and a version with changes tracked as a marked-up manuscript. It may also be helpful to check the guidelines for revised papers at http://journals.plos.org/plosmedicine/s/revising-your-manuscript for any that apply to your paper.

We ask that you submit your revision by Jun 29 2026 11:59PM. However, if this deadline is not feasible, please contact me by email, and we can discuss a suitable alternative.

Don't hesitate to contact me directly with any questions (sbruijn@plos.org).

Best regards,

Suzanne

Suzanne De Bruijn, PhD

Associate Editor

PLOS Medicine

sbruijn@plos.org

-----------------------------------------------------------

Comments from the academic editor:

This study tackles a critical scientific question in cardiovascular risk prediction. Current risk models in many countries have reached a plateau, and the integration of plasma proteomic signatures is a timely and promising frontier. Notably, shifting the endpoint from hard cardiovascular events to measures of subclinical atherosclerosis progression is a thoughtful and clinically relevant approach.

-----------------------------------------------------------

Comments from the reviewers:

Reviewer #1: The authors have addressed all of my questions, and I have no additional comments.

Reviewer #2: The manuscript is improved and much better balanced.

Note, NRI is the sum of differences in proportions and is thus not expressed as a percentage.

This needs to be corrected throughout.

Reviewer #3: I appreciate the effort made by the authors to address every one of the raised points (not only mine but also from the other reviewers). The authors have adequately addressed all my points except points 1 and 5:

Point 1.- the authors performed a stepwise logistic regression to select a subset of proteins independently and significantly associated to atherosclerosis (plaque and CAC), which was the initial idea. However, they used Akaike information criterion for candidate selection. While this procedure is adequate to construct a biomarker panel, again it does not demonstrate significant association of all the retained proteins. I would accept the result provided that throughout the manuscript the authors eliminate the wording "significantly associated with plaque/CAC" and consider the list of proteins as those that "configure an optimal panel for predicting the cases" or similar wording.

The authors claim that the proteins associated with CAC progression were "enriched in lipid and atherosclerosis pathways". This is truly incorrect; from inspecting Supp Table 6 there is no evidence for such an affirmation; just the contrary, the majority of proteins in these pathways are associated to plaque and not CAC progression. This must be corrected in the rsults secton to make them more consistent with the much more adequate interpretation made in the Discussion, where each protein is interpreted separately.

I have difficulties in understanding new results from Fig. 3. For instance in the case of plaque progression (right panel), why the proteins KFBP-2, MMP3, TMP4, HP, MCP1 are not there? Why are there proteins that are both not significant when the idea is to plot the proteins that are significant in plaque progression or ASCVD? Similar concerns apply to the left panel (CAC progression)

Point 5.- the results obtained using the protRS scores for predicting ASCVD are not compared with those obtained with CV risk factors in statistical terms. I mean that there are no p-values in Table 3 reflecting the significance of adding protRS scores to the CV risk scores. Note that these p-values are actually provided for the prediction of plaque and CAC progression; why are they not there for ASCVD?

Reviewer #4: Thanks for the revised manuscript and responses to my original review. The additional material resolved many of my original queries, there are three issues that I think can be resolved with minor revision.

I think the calibration plots are a good addition - these demonstrate that the CAC and plaques progression have reasonable levels of calibration overall (with some positive bias around the medium risk zone for plaque progression). The Homser-Lemeshow tests are unnecessary here - the bins are arbitrary and this test often reflects sample size rather than any important miscalibration. The important information is contained in the calibration plot itself.

In addition to DeLong's test, I would also add a likelihood ratio test (which tests model fit and parsimony)- this is more powerful to DeLong, and there are some circumstances in which the assumptions of the DeLong test are not appropriate (see https://doi.org/10.1002/sim.5328)

Supp Table 2. I think that a test for difference in baseline characteristics is not useful in this table - the important information about levels of difference in each variable between non-progressors and progressors can be seen from the summary statistics.

Any attachments provided with reviews can be seen via the following link: [LINK]

--------------------------------------------------------- ---

General editorial requests:

*Please upload any figures associated with your paper as individual TIF or EPS files with 300dpi resolution at resubmission; please read our figure guidelines for more information on our requirements: http://journals.plos.org/plosmedicine/s/figures. While revising your submission, we strongly recommend that you use PLOS's NAAS tool (https://ngplosjournals.pagemajik.ai/artanalysis) to test your figure files. NAAS can convert your figure files to the TIFF file type and meet basic requirements (such as print size, resolution), or provide you with a report on issues that do not meet our requirements and that NAAS cannot fix.

After uploading your figures to PLOS's NAAS tool - https://ngplosjournals.pagemajik.ai/artanalysis, NAAS will process the files provided and display the results in the "Uploaded Files" section of the page as the processing is complete.

If the uploaded figures meet our requirements (or NAAS is able to fix the files to meet our requirements), the figure will be marked as "fixed" above. If NAAS is unable to fix the files, a red "failed" label will appear above.

When NAAS has confirmed that the figure files meet our requirements, please download the file via the download option, and include these NAAS processed figure files when submitting your revised manuscript.

*Please ensure that the paper adheres to the PLOS Data Availability Policy (see http://journals.plos.org/plosmedicine/s/data-availability), which requires that all data underlying the study's findings be provided in a repository or as Supporting Information. For data residing with a third party, authors are required to provide instructions with contact information (web or email address) for obtaining the data. Please note that a study author cannot be the contact person for the data. PLOS journals do not allow statements supported by "data not shown" or "unpublished results." For such statements, authors must provide supporting data or cite public sources that include it.

* Data availability statement: Thank you for including the minimal data set. However, there is not sufficient information in your data availability statement. Please include where the original data can be found, and whether these are freely available. If they are not freely available, include reasons why, and contact details (email/URL) where researchers could request access.

*We expect all researchers with submissions to PLOS in which author-generated code underpins the findings in the manuscript to make all author-generated code available without restrictions upon publication of the work. In cases where code is central to the manuscript, we require the code to be made available as a condition of publication. Authors are responsible for ensuring that the code is reusable and well documented. Please make any custom code available, either as part of your data deposition or as a supplementary file. Please add a sentence to your data availability statement regarding any code used in the study, e.g. "The code used in the analysis is available from Github [URL] and archived in Zenodo [DOI link]" Please review our guidelines at https://journals.plos.org/plosmedicine/s/materials-software-and-code-sharing and ensure that your code is shared in a way that follows best practice and facilitates reproducibility and reuse. Because Github depositions can be readily changed or deleted, we encourage you to make a permanent DOI'd copy (e.g. in Zenodo) and provide the URL.

* Financial statement: Thank you for including your funding and the grant numbers. Please also include the initials of the researchers that were awarded the grant, as well as URLs to the funders' websites.

* The funding statement should include: specific grant numbers, initials of authors who received each award, URLs to sponsors’ websites. Also, please state whether any sponsors or funders (other than the named authors) played any role in study design, data collection and analysis, the decision to publish, or preparation of the manuscript. If they had no role in the research, include this sentence: “The funders had no role in study design, data collection and analysis, decision to publish, or preparation of the manuscript.”

* Please disclose any use of AI tools and technologies to the study or article content at the end of the Methods. Please specify the name(s) and version(s) of any tool(s) used and a description of their use, including how the AI-assisted or generated content was evaluated by authors. Please also include a statement specifying who retains responsibility for the final manuscript. Please see our guidelines for more information: https://journals.plos.org/plosmedicine/s/ethical-publishing-practice#loc-artificial-intelligence-tools-and-technologies.

* Please ensure that all abbreviations are defined at first use throughout the text.

* Please confirm that all numbers presented in the abstract are present and identical to numbers presented in the main manuscript text.

FORMATTING - GENERAL

* Please confirm that your title complies with PLOS Medicine's style. Your title must be nondeclarative and not a question. It should begin with main concept if possible. "Effect of" should be used only if causality can be inferred, i.e., for an RCT. Please place the study design ("A randomized controlled trial," "A retrospective study," "A modelling study," etc.) in the subtitle (ie, after a colon).

* In the last sentence of the Abstract Methods and Findings section, please describe the main limitation(s) of the study's methodology.

*At this stage, we ask that you include a short, non-technical Author Summary of your research to make findings accessible to a wide audience that includes both scientists and non-scientists. The Author Summary should immediately follow the Abstract in your revised manuscript. This text is subject to editorial change and should be distinct from the scientific abstract. Ideally each sub-heading should contain 2-3 single sentence, concise bullet points containing the most salient points from your study. In the final bullet point of 'What Do These Findings Mean?', please include the main limitations of the study in non-technical language. Please see our author guidelines for more information: https://journals.plos.org/plosmedicine/s/revising-your-manuscript#loc-author-summary.

*Please express the main results with 95% CIs as well as p values. When reporting p values please report as p<0.001 and where higher as the exact p value p=0.002, for example. Throughout, suggest reporting statistical information as follows to improve clarity for the reader "22% (95% CI [13%,28%]; p</=)". Please be sure to define all numerical values at first use.

*Please cite the reference numbers in square brackets. Citations should precede punctuation.

FIGURES AND TABLES

*Please consider avoiding the use of red and green in order to make your figure more accessible to those with color blindness. Please also check this for supplemental figures.

* Please define all elements of box plots in the figure caption - center line, box limits and whiskers.

* When a p value is given, please specify the statistical test used to determine it in the legend.

* Please consider if moving some of the figures shared in the supplementary information into the main text would aid the reader. Specifically, please consider moving Figure S1 into the main text.

SUPPLEMENTARY MATERIAL

*Please note that supplementary material will be posted as supplied by the authors. Therefore, please amend it according to the relevant comments outlined here.

*Please cite your Supporting Information as outlined here: https://journals.plos.org/plosmedicine/s/supporting-information

REFERENCES

*PLOS uses the numbered citation (citation-sequence) method and first six authors, et al.

*Please ensure that journal name abbreviations match those found in the National Center for Biotechnology Information (NCBI) databases (http://www.ncbi.nlm.nih.gov/nlmcatalog/journals), and are appropriately formatted and capitalised.

*Where website addresses are cited, please include the complete URL and specify the date of access (e.g. [accessed: 12/06/2023]).

*Please also see https://journals.plos.org/plosmedicine/s/submission-guidelines#loc-references for further details on reference formatting.

OBSERVATIONAL STUDIES

*Abstract: Please include the study design, population and setting, number of participants, years during which the study took place (enrollment and follow up), length of follow up, and main outcome measures.

*Please ensure that the study is reported according to the STROBE (or appropriate STOBE extension) guideline (available from: https://www.equator-network.org/reporting-guidelines/strobe) and include the completed STROBE (or STROBE extension) checklist as Supporting Information. Please add the following statement, or similar, to the Methods: "This study is reported as per the Strengthening the Reporting of Observational Studies in Epidemiology (STROBE) guideline (S1 Checklist)." When completing the checklist, please use section and paragraph numbers, rather than page numbers.

*Please also ensure that the study is reported according to the TRIPOD guideline (https://www.tripod-statement.org/) and include the completed TRIPOD checklist as Supporting Information. Please add the following statement, or similar, to the Methods: "This study is reported as per the Transparent Reporting Of a Multivariate Prediction Model (TRIPOD) guideline (S1 Checklist)." When completing the checklist, please use section and paragraph numbers, rather than page numbers.

*For all observational studies, in the manuscript text, please indicate: (1) the specific hypotheses you intended to test, (2) the analytical methods by which you planned to test them, (3) the analyses you actually performed, and (4) when reported analyses differ from those that were planned, transparent explanations for differences that affect the reliability of the study's results. If a reported analysis was performed based on an interesting but unanticipated pattern in the data, please be clear that the analysis was data driven.

*Please state in the Methods section whether the study had a prospective protocol or analysis plan. If a prospective analysis plan (from your funding proposal, IRB or other ethics committee submission, study protocol, or other planning document written before analyzing the data) was used in designing the study, please include the relevant document(s) with your revised manuscript as a Supporting Information file to be published alongside your study and cite it in the Methods section. A legend for this file should be included at the end of your manuscript. If no such document exists, please make sure that the Methods section transparently describes when analyses were planned, and when/why any data-driven changes to analyses took place. Changes in the analysis, including those made in response to peer review comments, should be identified as such in the Methods section of the paper, with rationale.

# Author comment: Author response to Decision Letter 2

**DOI:** 10.1371/journal.pmed.1005265.r005

22 Jun 2026

Attachment Submitted filename: Response_Letter_auresp_2.docx

# Peer review: Decision Letter 2

**DOI:** 10.1371/journal.pmed.1005265.r006

**Authors:** Suzanne De Bruijn

29 Jul 2026

Dear Dr Lu,

Many thanks for submitting a revised version of your manuscript "Plasma proteomic signatures driving subclinical atherosclerosis progression and their potential for cardiovascular risk stratification: A prospective cohort study" (PMEDICINE-D-26-01373R2) to PLOS Medicine.

The paper has been seen again by two of the reviewers. As you will see, the reviewers thought the manuscript has improved, but reviewer #3 has remaining concerns. After discussing the paper with the editorial team and an academic editor with relevant expertise, I'm pleased to invite you to revise the paper to address these remaining comments. We plan to send the revised paper to some or all of the original reviewers, and we cannot provide any guarantees at this stage regarding publication.

In addition to these comments, we also ask you to address some editorial requests, which you can find at the bottom of this email. Please include these in your point-by-point response.

In addition to these revisions, you may need to complete some formatting changes, which you will receive in a follow up email. A member of our team will be in touch with a set of requests shortly. If you do not receive a separate email within a few days, please assume that checks have been completed, and no additional changes are required.

When you upload your revision, please include a point-by-point response that addresses all of the reviewer and editorial points, indicating the changes made in the manuscript and either an excerpt of the revised text or the location (eg: page and line number) where each change can be found. Please also be sure to check the general editorial comments at the end of this letter and include these in your point-by-point response. When you resubmit your paper, please include a clean version of the paper as the main article file and a version with changes tracked as a marked-up manuscript. It may also be helpful to check the guidelines for revised papers at http://journals.plos.org/plosmedicine/s/revising-your-manuscript for any that apply to your paper.

We ask that you submit your revision by Aug 19 2026 11:59PM. However, if this deadline is not feasible, please contact me by email, and we can discuss a suitable alternative.

Don't hesitate to contact me directly with any questions (sbruijn@plos.org).

Best regards,

Suzanne

Suzanne De Bruijn, PhD

Associate Editor

PLOS Medicine

sbruijn@plos.org

-----------------------------------------------------------

Comments from the reviewers:

Reviewer #3: The authors have addressed my concerns about the wording (eliminate association) and the proteins in Fig. 3 and also in point 5. However, they have not resolved my concern about the interpretation of the KEGG enrichment analysis. There are several sentences that are incorrect:

"Subsequent KEGG pathway analysis revealed that a set of proteins within the

optimal panel for plaque progression (e.g., C-C motif chemokine 24 [CCL-24] and

interleukin-8 [IL-8]) were primarily implicated in inflammatory processes, including

cytokine signaling and NF-κB activation. Several proteins within the optimal panel for

CAC progression were included in pathway linked to lipid and atherosclerosis (e.g.,

low-density lipoprotein receptor), whereas a subtly different protein set for plaque

progression was mapped to the same pathways (e.g., monocyte chemotactic protein 1

[MCP-1])."

These sentences are completely misleading, since although there are some proteins for plaque progression implicated in inflammatory processes there are also many proteins for CAC progression also implicated in the same processes (namely four out of 10 in the cytokine category and two out of 4 in the NF-KB category come from CAC progression). And in the lipid and atherosclerosis category the majority are from plaque progression (five out of six!) and not from CAC progression. This is not a subtly different set, the truth is that both kind of proteins (for plaque and for CAC) are implicated in all these categories. Highlighting that plaque proteins are mostly associated to some categories and CAC proteins to others is completely misleading the reader towards a biased interpretation not supported by the data.

"Meanwhile, phenotype-specific protein signatures were also identified: plaque progression was

predominantly associated with inflammatory mediators including IL-8 and CCL-24 [38, 39],

whereas CAC progression was linked to atherosclerosis through low-density lipoprotein

receptor, a key regulator of cholesterol homeostasis that drives atherogenesis [40]"

This sentence is not only misleading but completely false, since the authors claim that the "specificity" of the protein signatures is vinculated to inflammation in the case of plaque and atherosclerosis in the case of CAC. As commented above, both kind of proteins are associated to both inflammation and atherosclerosis, hence this assertion on specificity is false.

Reviewer #4: Thanks for the revised manuscript and responses to my original review. The revised manuscript and responses to my questions have resolved my original questions.

Any attachments provided with reviews can be seen via the following link: [LINK]

--------------------------------------------------------- ---

General editorial requests:

* Thank you for including the limitation in your abstract and Author summary. However, I don't agree that the lack of cost-effectiveness is the main limitation. Please change the Abstract and the Author summary to include the main limitation of this study (e.g. no external validation).

* When a p value is given, please specify the statistical test used to determine it in the legend.

*Thank you for stating your study had a prospective analysis plan and including it. Please also ensure all changes to the analysis-- including those made in response to peer review comments-- are identified as such in the Methods section of the paper, with rationale.

* Please confirm that your title complies with PLOS Medicine's style. Your title must be nondeclarative and not a question. It should begin with main concept if possible. "Effect of" should be used only if causality can be inferred, i.e., for an RCT. Please place the study design ("A randomized controlled trial," "A retrospective study," "A modelling study," etc.) in the subtitle (ie, after a colon).

* Please include a statement regarding use or non-use of AI tools and technologies at the end of the Methods. Please specify the name(s) and version(s) of any tool(s) used and a description of their use, including how the AI-assisted or generated content was evaluated by authors, and a clear statement of which aspects of the study, article contents, data, or supporting files were affected or generated by AI tool usage. Please also include a statement specifying who retains responsibility for the final manuscript. See our guidelines for more information: https://journals.plos.org/plosmedicine/s/ethical-publishing-practice#loc-artificial-intelligence-tools-and-technologies.

* The Data Availability Statement (DAS) requires revision. I appreciate your statement that the data are not freely available. Please describe the restrictions for data access in more detail.

C) If the data are not freely available, please describe briefly the ethical, legal, or contractual restriction that prevents you from sharing it. Please also include an appropriate institutional contact (institutional web or email address) for inquiries (again, this cannot be a study author).

# Author comment: Author response to Decision Letter 3

**DOI:** 10.1371/journal.pmed.1005265.r007

14 Aug 2026

Attachment Submitted filename: Response_Letter_auresp_3.docx

# Peer review: Decision Letter 3

**DOI:** 10.1371/journal.pmed.1005265.r008

**Authors:** Suzanne De Bruijn

27 Aug 2026

Dear Dr Lu,

On behalf of my colleagues and the Academic Editor, Pei Gao, I am pleased to inform you that we have agreed to publish your manuscript "Plasma proteomic signatures of subclinical atherosclerosis progression for cardiovascular risk stratification: A prospective cohort study" (PMEDICINE-D-26-01373R3) in PLOS Medicine.

Before your manuscript can be formally accepted I have one remaining request:

*Thank you for including the changes made in response to the reviewers in S1 Text. However, we would need this included in the methods. I would suggest the following change:

Line 133: “and a prospective protocol, including changes made in response to the reviewer comments, is presented in S1 Text.

Furthermore, you will need to complete some formatting changes, which you will receive in a follow up email. Please be aware that it may take several days for you to receive this email; during this time no action is required by you. Once you have received these formatting requests, please note that your manuscript will not be scheduled for publication until you have made the required changes.

In the meantime, please log into Editorial Manager at http://www.editorialmanager.com/pmedicine/, click the "Update My Information" link at the top of the page, and update your user information to ensure an efficient production process.

PRESS

We frequently collaborate with press offices. If your institution or institutions have a press office, please notify them about your upcoming paper at this point, to enable them to help maximise its impact. If the press office is planning to promote your findings, we would be grateful if they could coordinate with medicinepress@plos.org. If you have not yet opted out of the early version process, we ask that you notify us immediately of any press plans so that we may do so on your behalf.

We also ask that you take this opportunity to read our Embargo Policy regarding the discussion, promotion and media coverage of work that is yet to be published by PLOS. As your manuscript is not yet published, it is bound by the conditions of our Embargo Policy. Please be aware that this policy is in place both to ensure that any press coverage of your article is fully substantiated and to provide a direct link between such coverage and the published work. For full details of our Embargo Policy, please visit http://www.plos.org/about/media-inquiries/embargo-policy/.

Thank you again for submitting to PLOS Medicine. We look forward to publishing your paper.

Sincerely,

Suzanne De Bruijn, PhD

Associate Editor

PLOS Medicine
